# Computational modeling of pancreatic cancer patients receiving FOLFIRINOX and gemcitabine-based therapies identifies optimum intervention strategies

**DOI:** 10.1371/journal.pone.0215409

**Published:** 2019-04-26

**Authors:** Kimiyo N. Yamamoto, Akira Nakamura, Lin L. Liu, Shayna Stein, Angela C. Tramontano, Uri Kartoun, Tetsunosuke Shimizu, Yoshihiro Inoue, Mitsuhiro Asakuma, Hiroshi Haeno, Chung Yin Kong, Kazuhisa Uchiyama, Mithat Gonen, Chin Hur, Franziska Michor

**Affiliations:** 1 Department of Data Science, Dana-Farber Cancer Institute, Boston, MA, United States of America; 2 Department of Biostatistics, Harvard T.H. Chan School of Public Health, Boston, MA, United States of America; 3 Department of Stem Cell and Regenerative Biology, Harvard University, Cambridge, MA, United States of America; 4 Departments of General and Gastroenterological Surgery, Osaka Medical College Hospital, Osaka, Japan; 5 Department of Radiation Oncology, Massachusetts General Hospital, Boston, MA, United States of America; 6 Institute for Technology Assessment, Massachusetts General Hospital, Boston, MA, United States of America; 7 Center for Systems Biology, Center for Assessment Technology & Continuous Health (CATCH), Massachusetts General Hospital, Boston, MA, United States of America; 8 Mathematical Biology Laboratory, Department of Biology, Faculty of Sciences, Kyushu University, Fukuoka, Japan; 9 Department of Epidemiology and Biostatistics, Memorial Sloan-Kettering Cancer Center, New York, New York, United States of America; 10 Center for Cancer Evolution, Dana-Farber Cancer Institute, Boston, MA, United States of America; 11 The Broad Institute of Harvard and MIT, Cambridge, MA, United States of America; University of South Alabama Mitchell Cancer Institute, UNITED STATES

## Abstract

Pancreatic ductal adenocarcinoma (PDAC) exhibits a variety of phenotypes with regard to disease progression and treatment response. This variability complicates clinical decision-making despite the improvement of survival due to the recent introduction of FOLFIRINOX (FFX) and nab-paclitaxel. Questions remain as to the timing and sequence of therapies and the role of radiotherapy for unresectable PDAC. Here we developed a computational analysis platform to investigate the dynamics of growth, metastasis and treatment response to FFX, gemcitabine (GEM), and GEM+nab-paclitaxel. Our approach was informed using data of 1,089 patients treated at the Massachusetts General Hospital and validated using an independent cohort from Osaka Medical College. Our framework establishes a logistic growth pattern of PDAC and defines the Local Advancement Index (LAI), which determines the eventual primary tumor size and predicts the number of metastases. We found that a smaller LAI leads to a larger metastatic burden. Furthermore, our analyses ascertain that i) radiotherapy after induction chemotherapy improves survival in cases receiving induction FFX or with larger LAI, ii) neoadjuvant chemotherapy improves survival in cases with resectable PDAC, and iii) temporary cessations of chemotherapies do not impact overall survival, which supports the feasibility of treatment holidays for patients with FFX-associated adverse effects. Our findings inform clinical decision-making for PDAC patients and allow for the rational design of clinical strategies using FFX, GEM, GEM+nab-paclitaxel, neoadjuvant chemotherapy, and radiation.

## Introduction

**Fig 1:**
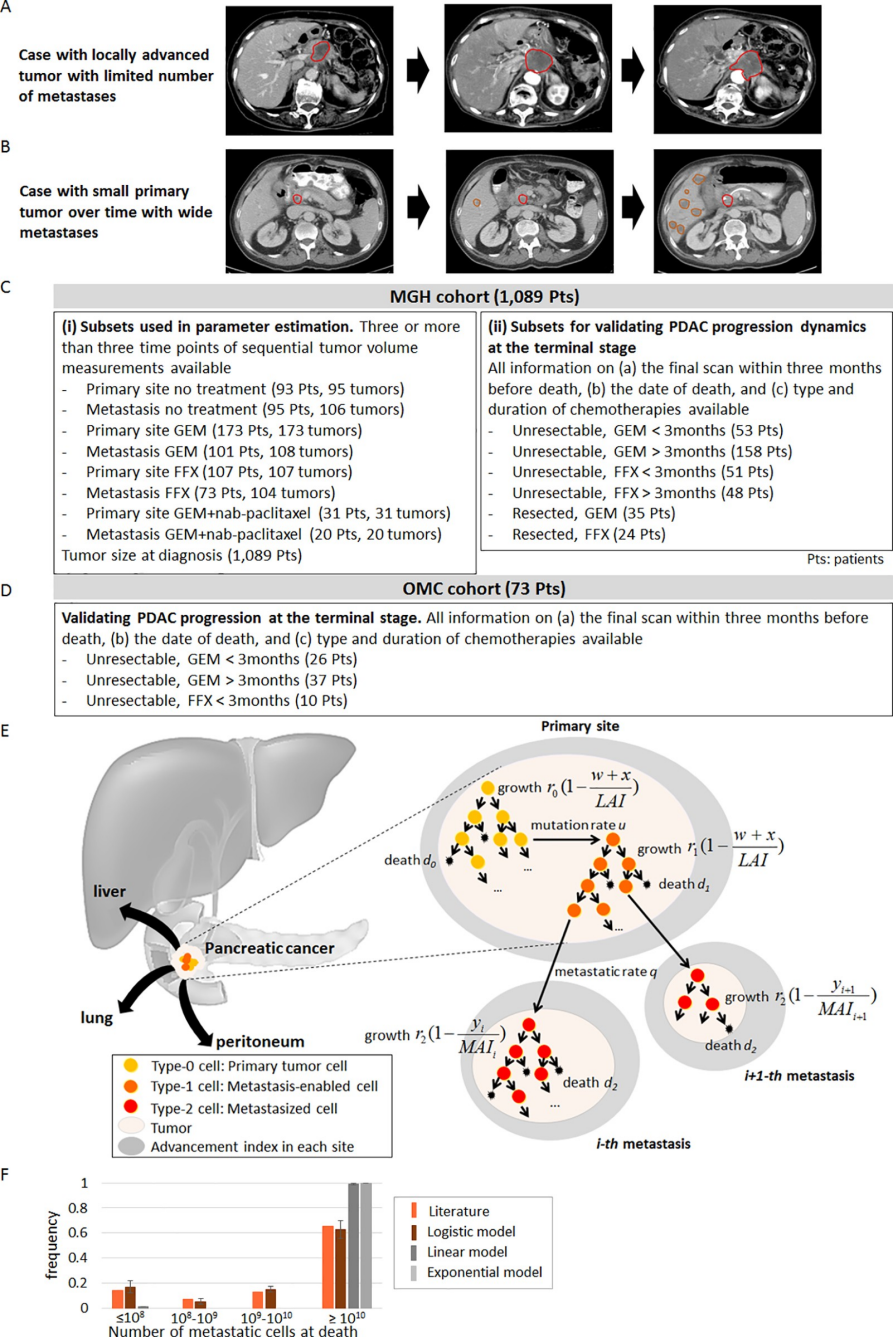
Disease progression phenotypes, clinical cohorts, and our computational framework together with the reproducibility of disease progression pattern. (A and B) Disease progression phenotypes of (A) patients with local progression without demonstrating features of metastases and (B) patients with widespread metastatic disease with a primary site confined to the pancreas. (C) The MGH cohort consisting of 1,089 patients was used for (i) model selection and parameter estimation and (ii) validation of the computational model of PDAC progression at the terminal stage. For model selection and parameter estimations, 742 tumors in 693 patients with sequential volume measurements during homogeneous treatments were analyzed using Bayesian inference. For validations of PDAC progression at the terminal stage, we utilized data of 369 patients who had all necessary information: (a) the final scan within two months before death; (b) the date of death; and (c) type and duration of CTx. The OMC cohort consisting of 73 patients who had all necessary information of (a)–(c) was used for validation of PDAC progression at the terminal stage. (E) Designed probabilistic model considering the situation in which the growth rates of tumor cells in the primary and metastatic sites decrease with increasing tumor size to reach a maximum volume (termed Local or Metastatic Advancement Index depending on whether the tumor is primary or metastatic). (F) Comparison of distribution of the number of metastatic cells at death from a rapid autopsy program of PDAC in a literature [4, 25] and three growth models: logistic, linear, and exponential models. Number of simulations at each growth model was 100 per each; and simulations were performed in quadruplicate. Mean and standard error for simulation results are indicated. See **S1(E) Doc** for details.

Pancreatic ductal adenocarcinoma (PDAC) remains one of the most devastating malignancies with a 5-year survival rate of 8% and is predicted to become the 2^nd^ leading cause of cancer-related death around 2020 [1, 2]. PDAC is a complex disorder composed of distinct progression patterns of local invasion and metastasis [3, 4]. A subset of patients die of complications caused by locally advanced pancreatic cancer (LAPC), including biliary sepsis and gastrointestinal obstruction (**Fig 1A**), while others succumb to widespread metastatic disease without presenting with intensive local invasion (**Fig 1B**) [3–5]. Whether a patient will develop widespread metastatic disease or local invasion is important for clinical decision-making; however, the course of disease remains difficult to predict in clinical practice [3, 4]. Hence, the development of a novel platform that fully depicts the divergence of PDAC progression phenotypes is needed.

Molecular determinants of the metastatic cascade in PDAC have been the topic of several investigations [3, 4, 6], and it has been shown that a loss of *SMAD4*, high levels of *RUNX3* in the context of intact *SMAD4*, and widespread epigenetic reprogramming confer metastatic potential [4, 7, 8]. In addition, genetic uniformity of driver mutations among primary and metastatic samples from the same patient has been observed [6, 9]. These findings collectively suggest that metastatic efficiency is determined by genetic and epigenetic alterations that arise during clonal expansion.

The introduction of gemcitabine (GEM) has led to survival improvements in both metastatic [10] and resectable disease [11]. The role of radiotherapy in unresectable disease is debated because phase III clinical trials have demonstrated that overall survival (OS) in regimens with radiotherapy is comparable to OS in regimens without radiotherapy [12]. More recently, the chemotherapy arsenal was updated to include two more combination regimens: FOLFIRINOX (FFX; folinic acid, fluorouracil, irinotecan, and oxaliplatin) [13] and GEM plus nab-paclitaxel [14]. In addition to survival advantages, FFX offers a high objective response rate [10, 13], particularly for local tumor control [15]. FFX does, however, lead to a higher incidence of adverse events including low blood counts, and complications such as fever, infection, diarrhea, weight loss, and fatigue limit the utility of FFX in elderly patient populations [13]. Due to therapeutic efficacy as well as differences in the rates of response and adverse events, the timing and sequence of therapies needs to be optimized. In particular, the role of radiotherapy in patients who received induction FFX, the potential impact of regimen adjustments due to drug toxicities, the impact of switching chemotherapy (CTx) after FFX discontinuation, and the efficacy of different treatment schedules in the adjuvant and/or neoadjuvant settings need to be investigated. Clinical studies aimed at answering these questions are challenging and can take years to mature.

Multiple studies have investigated tumor progression kinetics [16–23]. The paradigm of exponential growth was established early on based on the assumption that cancer cells divide in a completely unconstrained fashion [16]. This model represents an accurate description of early tumor growth but fails to incorporate dynamic changes of growth rates over time, for instance due to the increased limitation of nutrients, oxygen, and space as tumors increase in size. Because PDAC is characterized by a prominent desmoplastic/stroma reaction [24], and tumor volumes and growth rates are anti-correlated for both primary and metastatic tumors in PDAC patients [4, 25], a more comprehensive class of growth models accounting for dynamic changes of growth rates needs to be considered [21–23, 26]. The incorporation of these aspects into a single model will also enable the description of the inter-patient phenotypic heterogeneity of LAPC versus widespread metastatic PDAC [21–23].

Here we sought to construct a novel computational modeling platform of PDAC progression that integrates the complexity of PDAC progression phenotypes. Our approach was parameterized using the largest-to-date clinical cohorts of PDAC patients and allows for the evaluation of different treatment strategies using standard CTx regimens currently used to treat PDAC patients. Specifically, we utilized time-series tumor volume data for both primary and metastatic tumors of homogeneous treatment groups obtained from a large cohort treated at the Massachusetts General Hospital (MGH, **Fig 1C** and **Table 1**). Our model was subsequently validated using an independent database from Osaka Medical College (OMC, **Fig 1D** and **Table 1**) (**S1(A) Doc**). Our platform provides new insights into clinical decision-making for PDAC.

**Table 1:** Patient summary.

	Total MGH cohort	Cohort A	Cohort B	Cohort C	Cohort D	Cohort E	Cohort F	Cohort G	Cohort H	OMC Cohort
**Location/treatment**	Total	Primary/None	Metastasis/None	Primary/GEM	Metastasis/GEM	Primary/FFX	Metastasis/FFX	Primary/GEM+nab	Metastasis/GEM+nab	Total
**Number of patients**	1089	93	95	173	101	107	73	31	20	73
Age*	66(59–74)	66(58–72)	65(58–71.5)	67(58–73)	64(56–73)	61(55.5–68.0)	60(54–66)	68.2(63.5–73.5)	65.5(59–73)	63(55–74)
**Sex**										
**Male**	599(55.0%)	50(53.8%)	48(50.5%)	99(57.2%)	61(60.4%)	64(60.0%)	45(61.6%)	16(51.6%)	10(50.0%)	47(64.4%)
**Female**	490(45.0%)	43(46.2%)	47(49.5%)	74(42.8%)	40(39.6%)	43(40.0%)	28(38.4%)	15(48.3%)	10(50.0%)	26(35.6%)
**Race**										
**White**	945(86.8%)	78(83.9%)	76(80.0%)	159(91.9%)	92(91.1%)	94(87.9%)	67(91.8%)	27(87.1%)	17(85.0%)	0(0%)
**Asian**	37(3.4%)	7(7.5%)	10(10.5%)	6(3.5%)	4(4.0%)	5(4.7%)	1(1.4%)	2(6.5%)	2(10.0%)	73(100%)
**Black**	33(3.0%)	2(2.1%)	4(4.2%)	2(1.2%)	0(0.0%)	4(3.7%)	3(4.1%)	0(0%)	0(0%)	0(0%)
**Hispanic**	14(1.3%)	1(1.1%)	3(3.2%)	1(0.6%)	1(1.0%)	1(0.9%)	1(1.4%)	1(3.2%)	1(5.0%)	0(0%)
**Others**	60(5.5%)	5(5.4%)	2(2.1%)	5(2.9%)	4(4.0%)	3(2.8%)	1(1.4%)	1(3.2%)	0(0%)	0(0%)
**Marital status**										
**Yes**	690(63.4%)	65(70.0%)	67(70.5%)	120(69.4%)	73(72.3%)	77(72.0%)	55(75.3%)	23(74.2%)	14(70.0%)	NA
**No**	399(36.6%)	28(30.0%)	28(29.5%)	53(30.6%)	28(27.7%)	30(28.0%)	18(24.7%)	8(25.7%)	6(30.0%)	NA
Tumor size at diagnosis* (cm)	3.0(2.2–4.2)	3.0(2.3–3.8)	3.1(2.3–4.1)	3.3(2.5–4.4)	3.4(2.6–4.6)	3.4(2.4–4.1)	3.25(2.5–4.3)	3.4(2.6–4.4)	3.8(2.9–4.5)	NA
**Overall survival (months)**	21.9(19.2–25.8)	14.36(12.2–17.8)	14.1(12.0–18.5)	13.3(11.3–14.9)	11.1(9.7–12.5)	14.4(14.2–20.7)	14.9(13.7–20.4)	14.3(9.9–15.7)	14.4(9.6–15.3)	NA
**Received chemotherapy**										
**Yes**	772(70.9%)	70(75.3%)	79(83.2%)	173(100%)	101(100%)	107(100%)	73(100%)	31(100%)	20(100%)	73(100%)
**No**	317(29.1%)	23(24.7%)	16(16.9%)	0(0%)	0(0%)	0(0%)	0(0%)	0(0%)	0(0%)	0(0%)
**Received surgery**										
**Yes**	322(30.0%)	3(3.2%)	14(14.7%)	9(5.2%)	12(11.9%)	14(13.1%)	11(15.1%)	2(6.5%)	3(15.0%)	0(0%)
**No**	767(70.0%)	90(96.8%)	81(85.3%)	164(94.8%)	89(88.1%)	93(87.0%)	62(84.9%)	29(93.5%)	17(85.0%)	73(100%)
**Received radiation therapy**										
**Yes**	192(17.6%)	6(6.5%)	6(6.3%)	41(23.7%)	22(21.8%)	30(28.0%)	24(32.9%)	6(19.4%)	5(25.0%)	0(0%)
**No**	897(82.4%)	87(93.5%)	89(93.7%)	132(76.3%)	79(78.2%)	77(72.0%)	49(67.1%)	25(80.6%)	15(75.0%)	73(100%)
**Received proton therapy**										
**Yes**	83(7.6%)	5(5.4%)	4(4.2%)	4(2.3%)	3(3.0%)	8(7.5%)	5(6.9%)	2(6.5%)	2(10.0%)	0(0%)
**No**	1006(92.4%)	88(94.6%)	91(95.8%)	169(97.7%)	98(97.0%)	99(92.5%)	68(93.2%)	29(93.5%)	18(90.0%)	73(100%)

Abbreviation: GEM+nab = GEM+nab-paclitaxel.

Asterisks indicate that values are shown as median (first and third quartiles)

## Results

### A novel computational platform of PDAC patient data identifies in vivo growth kinetics

We first designed a stochastic mathematical model of cell growth, death, mutation accumulation, and dissemination to describe PDAC progression (**Fig 1E** and **S1(B)–S1(D) Doc**). In the model, the primary tumor consists of two cell types, one with (type-0 cells) and one without (type-1 cells) the potential to disseminate. This modeling choice was made based on evidence that metastatic ability can be the consequence of a single genetic or epigenetic change, such as genetic inactivation of *SMAD4*, high expression levels of *RUNX3*, or epigenetic reprogramming [4, 6–8, 25]. Type-0 cells divide and die at specified rates per time unit and may give rise to type-1 cells with a mutation rate *u* per cell division event. Type-1 cells also divide and die at given rates per time unit (**Fig 1E**). The stochastic model accounts for both situations in which PDAC cells acquire the ability to metastasize very early on and situations in which this phenotype arises later during tumor progression, depending on when the first surviving type-1 cell arises in the stochastic model. Furthermore, the model contains disseminated (type-2) cells that reside within metastatic sites [4, 25] and arise from type-1 cells at rate *q* per time unit. Each time a metastasis event occurs, a new metastatic colony is formed in which type-2 cells proliferate and die according to their specified rates. After PDAC diagnosis, both the primary tumor and metastases expand in cell number over time, eventually reaching a fatal tumor burden; see **S1(B)–S1(D) Doc** for details.

In order to parameterize our model, we obtained longitudinal sequential computed tomography (CT) imaging from 1,089 patients treated at the Massachusetts General Hospital (MGH) (**Table 1**). These patients are subdivided into cohorts: cohorts A and B contain patients with time series data in the absence of treatment for primary and metastatic sites; cohorts C and D contain patients treated with GEM for primary and metastatic sites; cohorts E and F contain patients treated with FFX for primary and metastatic sites; and cohorts G and H contain patients treated with FFX for primary and metastatic sites, respectively. In total, we analyzed data from 693 patients (742 tumors) with at least three sequential time points during a consistent treatment regimen consisting of either no treatment or homogeneous chemotherapy regimens (**Fig 1C(i)**); the remaining 396 patients were excluded because they did not have sufficient sequential data.

We first utilized time series tumor volume data in the absence of treatment to determine the growth kinetics of PDAC. To this end, we compared the goodness-of-fit of various growth models (Eqs (1)–(3) in **Materials and Methods**). In the exponential model (Eq 1), tumor cells divide in a completely unconstrained fashion with a constant growth rate [25]. In the logistic model (Eq 2), tumor cells divide at a rate that decreases with increasing tumor size such that the number of cells is given by an increasing curve that converges on a maximum size (**S1 Fig**). The maximum capacity of the primary site is defined as the Local Advancement Index (LAI), and that of a metastatic site as the Metastatic Advancement Index (MAI); both indices are measured in cm^3^. In the linear model (Eq 3), the tumor cell number increases linearly with time. To account for inter-patient variability, we considered both fixed and random effects of growth rates and LAI/MAI for both primary and metastatic sites for each patient. When using the Akaike Information Criterion (AIC) to assess model fits, we found that the logistic model provided the best fit to the data (**S1 Table and S1 Data**). However, since exponential and logistic models lead to similar predictions for early phases of tumor progression, we utilized an additional criterion to evaluate the predictive accuracies of the individual growth models based on the growth patterns at the final stages of tumor growth (**Fig 1F**). When investigating the extent of metastatic burden at death using logistic, linear, and exponential growth assumptions in the stochastic model, we found that the logistic model was able to recapitulate the metastatic burden at death observed previously using data obtained within a PDAC rapid autopsy program [4, 25]. The exponential and linear models led to worse predictions (p = 0.608, <0.001, and <0.001 for simulations with logistic, linear, and exponential models, respectively, Mann-Whitney test, **Fig 1F)**. Thus, we chose the logistic growth model for describing PDAC growth and progression patterns.

Based on the logistic stochastic model, we then estimated primary and metastatic growth rates as well as LAI/MAI (i) in the absence of treatment, (ii) during GEM, (iii) during FFX, and (iv) during GEM+nab-paclitaxel treatment (**S2 and S3 Datas**). Growth rates during CTx and in the absence of treatment were estimated using corresponding sequential volume measurement data. Median and first and third quartiles of the estimates (in units of 1/month) are shown in **S2 Table**. We found that growth rates for both primary sites and metastases during FFX were significantly smaller than those during GEM-based therapies or in the absence of treatment (p-values in **S2 Table)**. We also investigated the sensitivity of estimations to changes in prior distributions to examine the influence of the choice of prior on the posterior distributions, and found robustness of the posterior estimates when using priors on all other parameters (**S3 Data and S1(F) Doc**).

### Patients with a smaller LAI have a larger number of metastases

**Fig 2:**
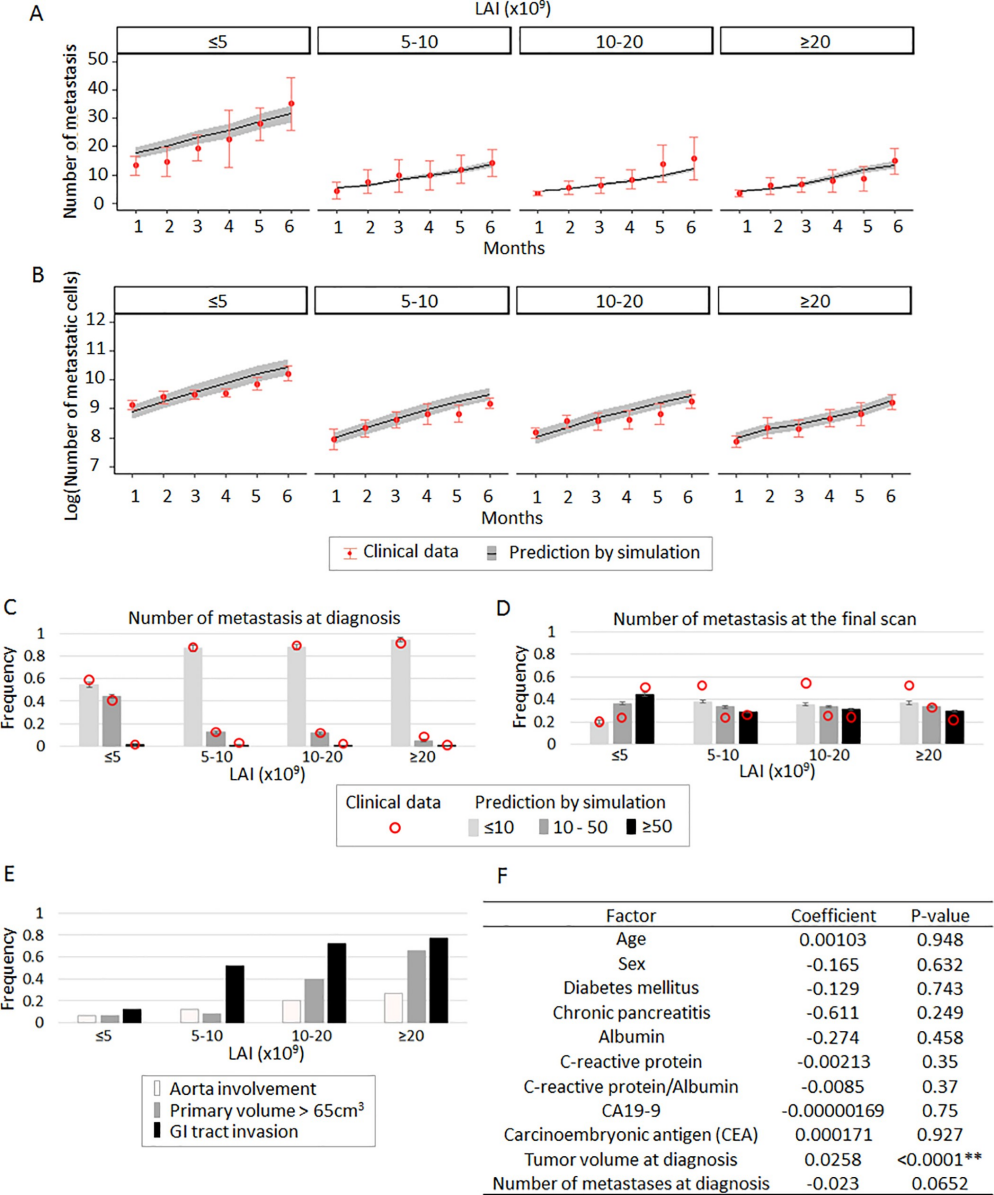
A small local advancement index (LAI) increases metastatic burden. (A and B) Time series of (A) the number of metastases and (B) the number of metastatic cells at the largest metastatic site according to the estimated LAI using clinical data. (C and D) Frequency of patients with the indicated number of metastatic sites (C) at diagnosis and (D) at the final scan according to the estimated LAI using clinical data. Numbers of patients were 16, 26, 25, and 26 for patients with LAI<5×10^9^, 5×10^9^≤LAI< 10×10^9^, 10×10^9^≤LAI<20×10^9^, 20×10^9^≤LAI, respectively, in clinical data; number of simulation cases was 100 per each in panels (A)–(D), performed in quadruplicate. Mean and standard error are indicated for both clinical data and simulation results in panels (A)–(D). Polyserial correlation coefficients between LAI and the number of metastases of ≤10, 10–50, or ≥50 were 0.931, -0.927, or -0.999, respectively in (C); and 0.810, -0.764, -0.816, respectively in (D). (E) Frequency of patients with indicated complications induced by local progression at the final scan according to the estimated LAI using clinical data. Polyserial correlation coefficients between LAI and each of aorta involvement, primary tumor > 65 cm^3^, and GI tact invasion, were 0.999, 0.984, and 0.999, respectively. (F) Univariate analysis to test the statistical significance of patients’ clinicopathological factors on LAI. Parameter values were *u* = 6.31×10^−5^, *q* = 6.31×10^−7^, *r*_*0*_ *and r*_*1*_ = 0.28, *r*_*2*_ = 1.16, death rate of each type = 0.01×growth rate, *M*_*diag*_ = 10^9.47^. MAI for primary and metastatic sites are based on the estimated distributions (**S2 Data**).

We next hypothesized that disease progression phenotypes in PDAC, such as local invasiveness or widespread metastasis, are correlated with a patient’s LAI. Primary tumors with a large LAI sustain a large population of cells, resulting in locally progressive disease. Patients with a small LAI contain a smaller population of cells in the primary site, thereby potentially leading to widespread metastatic disease. We found that both the number of metastatic sites and the number of cells at the largest metastatic site increased after diagnosis in simulated patients with a smaller LAI, defined as less than 5×10^9^ cells, as compared to other patients (**Fig 2A and 2B**); these findings were confirmed using patient data (**Fig 2A and 2B**). Moreover, simulated patients with a small LAI presented with a large metastatic burden both at diagnosis and the final scan (polyserial correlation coefficients in the legend, **Fig 2C and 2D**); again, these findings were confirmed using patient data (**Fig 2C and 2D**). Note that the model was originally parameterized using longitudinal imaging data of primary and metastatic sites but confirmed using different aspects of the data, such as the numbers of metastases at diagnosis or death.

Patients from the MGH database with a large LAI suffered from significantly more complications by local invasion (tumor volume over 65cm^3^, aorta involvement, and gastrointestinal invasion) than other patients (polyserial correlation coefficients in the legend, **Fig 2E**). When investigating the relationship between LAI and various clinicopathological factors in the MGH cohort, we observed a correlation between estimated LAI and tumor volume at diagnosis and a weak correlation between LAI and the number of metastases at diagnosis (**Fig 2F**). In sum, our parameterized computational framework was able to accurately capture the relationship between the size of LAI and metastatic progression.

### The effect of CTx on PDAC progression phenotypes

**Fig 3:**
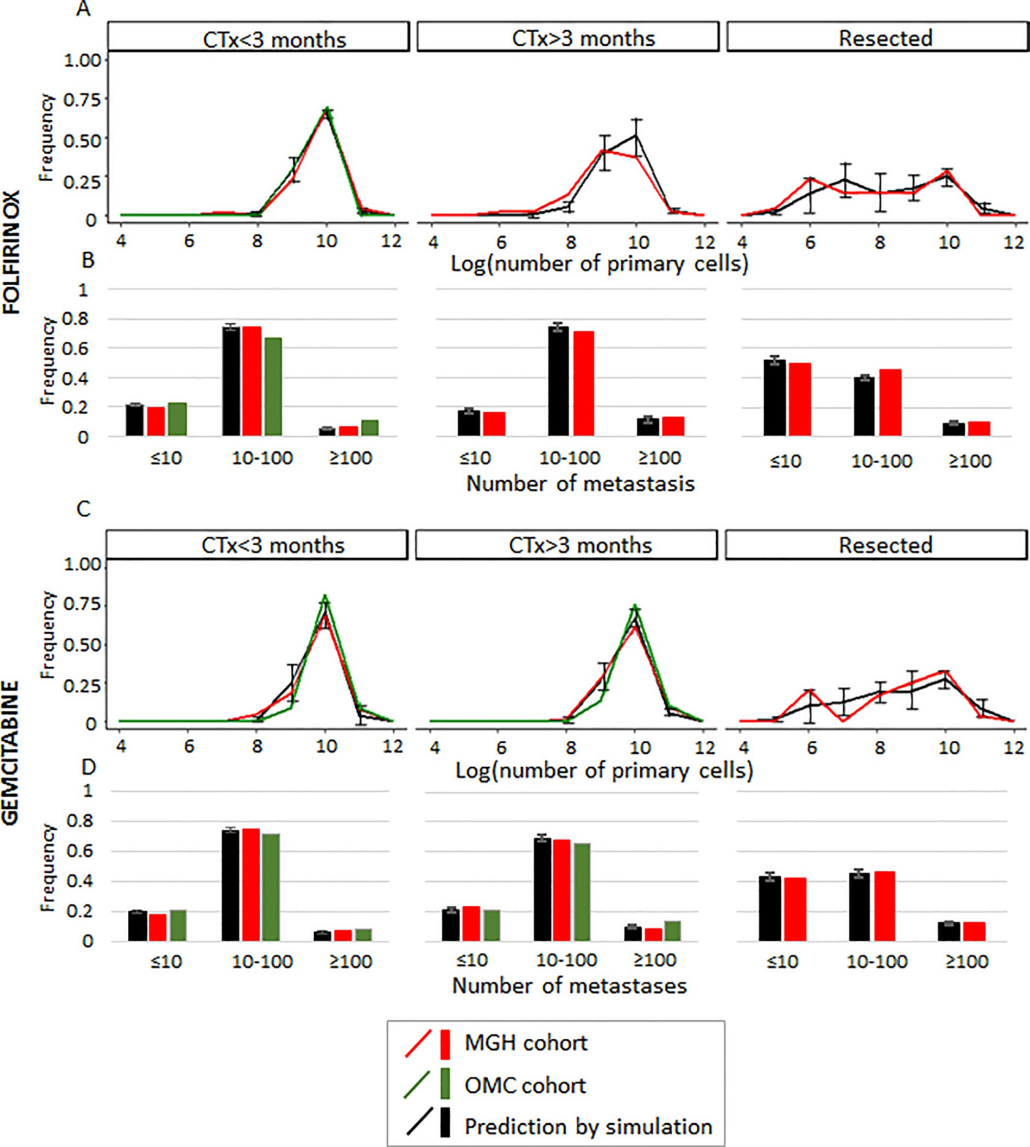
Predictions of the computational framework are validated using clinical data. Frequency of cases with (A) the number of primary cells on a logarithmic scale with base 10 and (B) the number of metastatic sites at the final scan in unresectable patients who received FFX for less than three months, unresectable patients who received FFX for three or more than three months, and patients after surgical resection of their primary site. (C and D) The quantities described above in patients who received GEM are shown in the same order. The number of simulation cases was the same as number of patient at each MGH cohort in each category described in **Fig 1C(ii),** i.e, 51, 48, and 24 for FFX; and 53, 158, and 35 for GEM, in cases with CTx<3 months, CTx>3 months, and with resection, respectively; and simulations in any categories were performed in quadruplicate. Mean and standard error for simulation results are indicated. Parameter values used for the panels were *u* = 6.31×10^−5^, *q* = 6.31×10^−7^, *r*_*0*_ *and r*_*1*_ = 0.28, *r*_*2*_ = 1.16, death rate of each type = 1/100×growth rate, *M*_*diag*_ = 10^N(9.47,0.29)^, and *M*_*death*_ = 10^10.6^. LAI/MAI and growth rates during CTx for primary and metastatic sites are based on the estimated distributions (**S2 Data**), CTx duration = 1.5 and 6 months that correspond to median CTx duration in the clinical cohort in groups that received CTx with (i) less than three months and (ii) three or more than three months, respectively, and *ε* was randomly chosen from [10^−5^, 10^−1^].

We then sought to investigate the effects of different treatment types and durations on tumor characteristics and patient outcomes. For simulated cases with unresectable PDAC who received more than 3 months of FFX, we observed smaller sizes of the primary tumors at the final scan as compared to those receiving fewer than 3 months (median 9.90 and 9.35 for ≤3 and >3 months, respectively, p<0.0001, **Fig 3A**). In contrast, the duration of GEM did not affect the primary tumor sizes in our simulations (median 9.92 and 9.89 for ≤3 and >3 months, respectively, p = 0.928, **Fig 3C**), as expected from the large estimated growth rates during GEM treatment. Meanwhile, among simulated cases with resections of their primary tumors, we observed two peaks in the distribution of the primary tumor sizes at the final scan **(Fig 3A and 3C**); the right peak represents disease recurrence from residual tumor cells in the primary site, while the left peak represents post-surgical tumor remnants confined to the pancreas (**Fig 3A and 3C**). In both simulated FFX and GEM regimens, the numbers of metastases were predicted to be smaller in cases with resection of the primary site as compared to unresectable cases (p = 0.040 and 0.0185 in FFX and GEM, respectively, **Fig 3B and 3D**). These model predictions were again confirmed using clinical cohorts from MGH (**Fig 1C(ii))**. Furthermore, they were validated in an independent patient cohort obtained from OMC (**Fig 1D**). Overall, we found that model predictions were accurate for the local and distant tumor burden at the final scan **(Fig 3A**–**3D**). The stochastic modeling platform also correctly predicts survival as confirmed using patient data from the MGH cohort (**S2 Fig**).

In this study, untreated patients include patients who have not yet initiated treatment, who are in terminal stages, and for whom no information was available regarding prior treatment. Thus, this patient group might represent a heterogeneous cohort. To investigate the effects of differing growth rates among patients in such a heterogeneous cohort, we performed sensitivity analyses by assuming that drugs may reversibly modify growth rates at the time of treatment discontinuation. We found that differences in the level that growth rates recover to post treatment discontinuation, and the speed at which this reversal occurs, do not affect the results (**S5 Fig**). These findings support the robustness and generalizability of our computational framework.

### Optimum treatment strategies for PDAC patients

**Fig 4:**
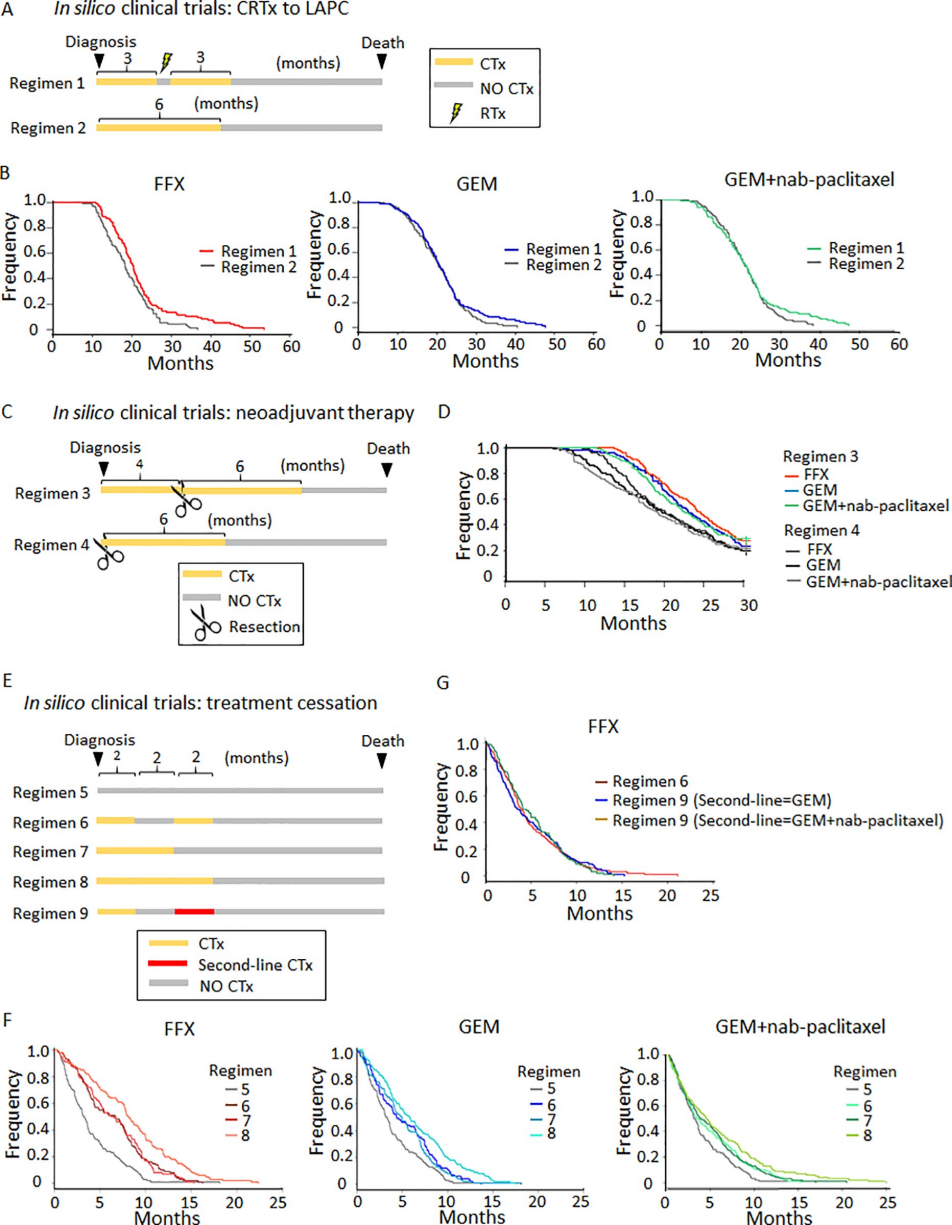
Prediction of optimum treatment schedules for PDAC. (A) Description of CRTx schedules in cases with LAPC. (B) Assessment of survival duration of regimens 1 and 2 in each drug. P-values by log-rank test were p = 0.008, 0.236, and 0.253 for FFX, GEM, and GEM+nab-paclitaxel, respectively. Subgroup analyses were shown in **S3 Fig**. (C) Description of neoadjuvant and/or adjuvant CTx schedules in cases with resectable disease. (D) Assessment of survival duration of regimens 3 and 4. P-values between regimen 3 and 4 were p<0.001 for any drugs. (E) Description of CTx schedules for cases with unresectable disease. (F) Assessment of survival duration of regimens with FFX, GEM, and GEM+nab-paclitaxel. P-values are shown in **S3 Table**. (G) Assessment of survival duration of regimens with four months FFX and two months cessation with various second-line drugs. P-values are shown in **S4 Table**. Parameter values were the same as those in **Fig 3**.

To evaluate the effects of different treatment strategies on survival, we designed *in silico* clinical trials of different scenarios using our validated computational modeling platform (**Fig 4 and S1(G) Doc**). First, we investigated whether the use of chemoradiation (CRTx) is effective for the treatment of LAPC [3]. Simulated LAPC cases were stratified into two arms: (i) three months of CTx followed by radiation (RTx) and three months of adjuvant CTx, and (ii) six months of CTx (**Fig 4A**). Interestingly, although the administration of CRTx after induction with GEM or GEM+nab-paclitaxel did not improve survival, the use of FFX did (**Fig 4B**). Furthermore, subgroup analyses showed that simulated cases with a larger than median LAI had a significantly better prognosis when adding CRTx compared to CTx (**S3 Fig**; p-values are 0.0547, 0.0429, and 0.0379 for FFX, GEM, and GEM+nab-paclitaxel, respectively), whereas simulated cases with a lower LAI had comparable OS when adding CRTx compared to CTx (**S3 Fig**; p-values 0.117, 0.809, and 0.466 for FFX, GEM, and GEM+nab-paclitaxel, respectively). These results indicate a significant role of LAI for identifying cases who will benefit from CRTx–those with a larger than median LAI.

We next investigated, again using *in silico* clinical trials, whether neoadjuvant CTx improves outcomes when added to the current standard of care of adjuvant protocols (**Fig 4C**) [27]. Simulated resectable cases were stratified into two arms: (i) four months of neoadjuvant CTx followed by resection and six months of adjuvant CTx and (ii) six months of CTx following resection (**Fig 4C**). We observed improved OS in simulated cases receiving the former compared with those receiving the latter for both GEM-based and FFX settings (**Fig 4D;** p<0.001 for any drug between regimens 3 and 4).

Finally, we investigated the effects of temporary cessations and switching CTx after FFX discontinuation. After PDAC diagnosis, simulated cases were randomized into four arms: (i) no CTx (regimen 5); (ii) four months CTx and two months cessation (regimen 6); (iii) four months CTx (regimen 7); (iv) six months CTx (regimen 8); and (v) four months CTx and two months cessation while treating with a different drug (regimen 9) (**Fig 4E**). The time period of two months for a drug holiday was chosen in order to ensure that sufficient time is provided for the drugs to be cleared from a patient’s body; clearance rates for each drug in the FFX regimen are 15 minutes, 8.8 hours, 14.1 minutes, and 7 hours, respectively [28–31], while the reported mean recovery time for FFX adverse effects is 11.5 days [32]. Based on this data we chose a two months drug holiday as a representative example for our *in silico* clinical trials.

When analyzing the simulated data, we found that the impact of CTx on OS was larger in simulated cases receiving FFX than GEM or GEM+nab-paclitaxel therapy (**Fig 4F and S3 Table**). Furthermore, a longer duration of CTx improved predicted OS in simulated cases receiving FFX or GEM-based therapies (**Fig 4F and S3 Table**) (p-values in **S3 Table**). Interestingly, simulated temporary cessations did not lead to significantly different OS compared to regimens without cessations, but with the same total treatment duration (**Fig 4F and S3 Table**). Moreover, OS of simulated cases receiving FFX and GEM-based therapies in the second-line setting after FFX failure were not statistically significantly different (p-values in **S3 and S4 Tables**) (**Fig 4G and S4 Table**). These findings provide a rational underpinning for evaluating best treatment regimens for testing in the clinic.

## Discussion

We have developed a novel computational modeling approach that was parameterized using the largest-to-date clinical cohort of PDAC patients. Our model captures the logistic tumor growth patterns observed in patients and can be used to estimate the eventual size a primary tumor will reach in a patient, termed the local advancement index (LAI) (**Fig 1**). Using our model, we found that patients with a small LAI are likely to develop widely metastatic disease, while patients with a large LAI tend to exhibit complications due to local tumor or progression with a small metastatic burden (**Fig 2**). The predictions from our computational modeling platform were then confirmed using clinical cohorts (**Fig 3**). These findings may provide new insights into clinical decision-making, suggesting that adjuvant systemic therapies could be necessary for patients with a small LAI who eventually develop widespread metastatic PDAC, while intensive local control as well as systemic therapies are necessary for patients with a large LAI.

In clinical practice, LAPC is commonly treated with CRTx due to its powerful local effects, although this treatment modality may reduce quality of life due to gastrointestinal obstruction, bleeding, jaundice, pain, and others, resulting in worse survival outcomes [3]. Here we demonstrated a potential role of LAI for identifying LAPC cases who will benefit from CRTx: we found that simulated cases with a larger LAI had significantly better OS when adding CRTx compared to cases with CTx, while simulated cases with a smaller LAI had comparable OS between CRTx and CTx regimens (**Fig 4B and S3 Fig**). The use of LAI as a marker in the clinic has not been a pragmatic approach because the estimation of LAI depends on the availability of sequential medical imaging data (**S1 Data**). However, the evaluation of LAI as a marker may be promising for cases in which multiple longitudinal tumor assessments were conducted before the initiation of treatment.

In addition, we found that CRTx leads to a significant survival benefit when FFX, but not when GEM or GEM+nab-paclitaxel are used (**Fig 4B**). These findings are in agreement with the results of the LAP-07 study, which showed no significant difference in survival with CRTx compared to CTx when either GEM or GEM/erlotinib were used, despite an improvement in local control [12]. The LAP-07 trial found that both GEM and GEM/erlotinib were insufficient to control micrometastases, thereby perhaps obscuring the benefits of local control due to CRTx for improving overall survival. In contrast, FFX may be more effective than GEM because it stabilizes both the local tumor and micro-metastases. Therefore, local treatment effects of CRTx are more pronounced, leading to improved overall survival (**Fig 4B and S2 Table**).

One caveat of our model is that the adverse effects of CRTx, such as hematologic and gastrointestinal toxicities, were not considered in the simulated trials, which could lead to a bias towards positive effects on our simulated survival. However, we found that CRTx reduces the primary tumor size at death, suggesting that patients receiving CRTx may have less severe local symptoms (**S4 Fig**) [3]. We also predicted that neoadjuvant CTx may improve survival for both GEM-based therapies and FFX regimens compared with CTx in the adjuvant setting (**Fig 4C and 4D**). Possible explanations for this finding include that reassessment after neoadjuvant CTx might efficiently exclude patients with progressively disseminated disease from undergoing surgery and that neoadjuvant CTx, although not curative, systemically reduces the number of metastatic cells before surgery, which otherwise might have expanded aggressively postoperatively [27]. Furthermore, a higher incidence of adverse events for FFX renders some patients in need of treatment discontinuation. Our analyses predict that temporary cessations of FFX do not negatively impact OS (**Fig 4F and S3 Table**), and that switching FFX to GEM-based therapies after FFX discontinuation does not negatively impact OS either (**Fig 4G and S4 Table**). Together, regimens with temporary cessations until patients recover to an acceptable performance status and regimens with GEM-based therapy as the second-line setting represent reasonable options.

Our validated computational modeling framework addresses another important issue: in the clinic, the same patient cannot be stratified into different treatment arms in a trial. It is thus impossible to know what the counterfactual outcome of a different treatment would have been in the same patient. Our *in silico* clinical trials tackle this important issue since each case can receive both standard and experimental arms, which enables us to evaluate the outcomes of several experimental regimens for exactly the same case. Our approach also has potential implications for the rational design of novel clinical strategies for patients with other cancer types for which similar data can be obtained.

## Materials and methods

### Clinical cohorts

We analyzed a total of 1,089 patients (599 men, 490 women) who were treated for PDAC at Massachusetts General Hospital (MGH) between October 2002 and September 2015 (**Table 1**). A validation cohort contained information on 73 PDAC patients (47 men, 26 women) with surgically unresectable disease who were treated between April 2008 and March 2016 at Osaka Medical College (OMC) in Japan (**Table 1**). This clinical dataset is unique in that longitudinal imaging data without any treatment can hardly ever be obtained in PDAC because most patients receive treatment soon after diagnosis. Data collection and analysis were approved by the Ethics Committees for Clinical Investigation of both MGH and OMC. Methods were carried out in accordance with the approved guidelines. **S1(A) Doc** provides more details.

### Computational modeling of PDAC progression

We designed a computational model of PDAC progression using a three-cell type logistic branching process starting from a single cell in the primary site. This cell gives rise to a clone of cells, so-called type-0 cells, that proliferate and die at specified rates. During each type-0 cell division, an alteration may arise at rate *u* that enables the cell, now called type-1 cell, to leave the primary site and establish a metastatic colony elsewhere; at that point a cell is called a type-2 cell. This modeling assumption is based on the findings that metastatic efficiency is determined by (epi)genetic alterations that arise during the clonal expansion of PDAC [4, 6–8] (**Fig 1E and S1 Fig**). In the model, the growth rate of each cell type decreases with increasing tumor size such that the number of cells is given by an increasing curve that converges on a maximal size, defined as local advancement index (LAI) for the primary and metastatic advancement index (MAI) for a metastatic site. The growth rates and LAI/MAI were estimated using the logistic model described in **S1(B) Doc**. The death rates of each cell type are assumed to be fixed as death rates = growth rate/100. One of the defining features of PDAC is the presence of extensive fibrosis. The desmoplastic stroma consists of proliferating fibroblasts and pancreatic stellate cells, inflammatory cells, nerve fibers, and marrow derived stem cells [33]. In this study, we assumed that the primary tumor consists of 80% stromal cells present [33]. We converted the tumor volume to cell numbers with the assumption of a spherical shape (10^9^ cells occupy a volume of 1 cm^3^) [25]. See **S1(B) and S1(C) Doc** for details.

### Computer simulations before and after diagnosis

We performed *in silico* trials of the stochastic process based on our mathematical modeling. Once a tumor has been diagnosed, we implemented clinical practice based on the guidelines of PDAC treatment provided by the National Comprehensive Cancer Network (NCCN) in the United States in the context of the computational framework [34]. If no metastases are detected at diagnosis, a case is regarded as non-metastatic disease and the patient becomes the candidates for either surgery or CRTx. If any metastases are detected, a patient receives CTx without surgical resection nor RTx.

- **Resection:** to remove a fraction (1-*ε*) (0 ≤*ε*≤ 1) of the primary tumor so that the remnant tumor volume becomes *ε* of the primary tumor. The parameter *ε* is randomly chosen from [10^−5^, 10^−1^].
- **CTx:** The growth rates of cells during CTx (GEM or FFX) were estimated by the corresponding clinical data of patients during treatment with these drugs. Specifically, we utilized three or more time points of sequential volume measurements in primary and metastatic sites during each treatment. We fitted a logistic model, which was selected as the best model. We confirmed a good fit to clinical data in each cohort and then estimated growth rates during each CTx (**S1 and S2 Datas**). In this study, we assumed that drugs reversibly modify growth rates at the time of treatment discontinuation.
- **RTx:** The conventional long course 3D-CRT has been assumed. The effect of conventionally fractioned RTx was determined by using the Linear-Quadratic model [35]. In this framework, the surviving fraction of radiated cells is given by *e*^*-(ωD+ξD2)*^, where *ω* and *ξ* are constants and *D* is the dose. We consider *ω* = 10*ξ*, which is a well-accepted quantity for cancer cells [36], that RTx with a total dose of 54 Gy was delivered in 30 fractions, and D is 1.8 Gy. For more details of treatment effects by CTx, RTx, and surgery, see **S1(D) Doc.**

### Computational studies for optimum treatment

To evaluate the effects of different treatment strategies on PDAC patients, we designed *in silico* computational clinical trials. We explored three scenarios: (i) CRTx to cases with LAPC (**Fig 4A**); (ii) neoadjuvant CTx followed by standard adjuvant care (**Fig 4C**); and (iii) administration of CTx at different timing in unresectable patients (**Fig 4E**). Note that all cases are virtual but not real clinical patients from MGH/OMC. Simulations are conducted based on each of trial designs in **Fig 4** so that we can mimic clinical trials in computers. Their parameters are determined according to distributions which have been informed by the clinical cases from MGH. In these scenarios, cases were randomized into different treatment groups after diagnosis, and simulations were performed until death in each case. See **S1(F) Doc** for sample size estimation.

### Model fitting and statistical analysis

We fit mixed effects models (exponential, logistic and linear models) to each individual patient’s data on both primary and metastatic sites. The exponential model is given by

$$Volume=e^{\left(\beta +b_{i}\right)}\times e^{\left(\beta_{r}+b_{ri}\right)}\times {DATE}_{i,j}$$ (1)

where *β* and *b*_*i*_ are fixed and random effects of intercept for patient *i*, *β*_*r*_ and *b*_*ri*_ are fixed and random effects of growth rates, and *DATE*_*i*,*j*_ is the time period (month) at *j-*th measurement occasion from the first measurement, respectively. The logistic model is given by

$$Volume=\frac{\beta_{Carry}+b_{{Carry}_{i}}}{1+B\times e^{\frac{-1}{\beta_{r}+b_{r_{i}}}\times {DATE}_{i,j}}}$$ (2)

where *β*_*Carry*_ and *b*_*Carry_i*_ are fixed and random effects of LAI/MAI, and *β*_*r*_ and *b*_*ri*_ are fixed and random effects of growth rates, respectively. Only fixed effects were considered for parameter *B* in the fitting because of identifiability issues. For parameter estimations of LAI/MAI and growth rates for primary and metastases, we performed Bayesian inference with Markov chain Monte Carlo methods (MCMC) sampling using stan (**S1(I) Doc**) [37]. Prior distributions were obtained from *N*(0.16, 0.14) and *N*(0.58, 2.72) for primary and metastatic sites, respectively, based on a previous study [25]. Prior distributions for *LAI* and *B* were *LN*(0, 10) and *LN*(0, 10), respectively. The linear model is given by

$$Volume=(\alpha +a_{i})\times {DATE}_{i,j}+(\beta +b_{i})$$ (3)

See statistical analyses in **S1(F) Doc** for more details.

**S1(H) Doc** contains supplementary discussion.

## Supporting information

S1 Data Growth rates, LAI/MAI for primary and metastatic sites. The estimated growth rates and LAI/MAI values for both primary and metastatic sites for each patient are provided.
(XLSX)

S2 Data Estimated growth curve and its comparison with clinical data. The estimated growth curves with the logistic model (solid line) and the exponential model (dotted line) for each patient are shown and the tumor sizes at the timing of each medical examination in the clinical data are also plotted as red square.
(PDF)

S3 Data Estimated parameters for a mixed effects logistic model. (i)-(iv) Assessments of growth rates, LAI and B by Bayesian estimation using volume measurements of primary tumors in different treatment regimens. (v)-(viii) Assessments of growth rates, MAI, and B by Bayesian estimation using volume measurements of metastatic tumors in different treatment regimens.
(DOCX)

S1 Doc Supplementary description. The descriptions include an explanation or discussion regarding (a) Clinical cohorts, (b) Computational modeling of PDAC progression, (c) Computer simulations before diagnosis, (d) Computer simulations after diagnosis, (e) Three-step branching process with different growth models, (f) Statistical analysis, (g) Computational studies for optimal treatment, (h) Supplementary discussion, (i) Stan code for Bayesian inference, and (j) Evaluation of the accuracy of LAI.
(DOCX)

S1 Fig Schematic illustration of the computational model. We considered a model of logistic expansion of the number of cancer cells starting from a single cell in the primary site. Cancer cells follow a stochastic process: during each elementary time step, cells may divide with a possibility of accumulating an alteration that allows it to divide, die, or metastasize elsewhere. We considered the situation in which the growth rate of the tumor decreases with increasing tumor size. Cells that have not yet evolved the ability to metastasize, type-0 cells, divide at rate *r*_*0*_(1-(*w+x*)*/LAI*) and die at rate *d*_*0*_ per unit time. Type-0 cells give rise to type-1 cells through accumulating an alteration in a metastatic-related gene with probability *u* per type-0 cell division. Type-1 cells divide and die at rates of *r*_*1*_(1-(*w+x*)*/LAI*) and *d*_1_ per unit time. Type-1 cells can establish a metastatic colony, consisting of type-2 cells, at another location with probability *q*; these sites start from a single metastatic cell in each metastatic site. Type-2 cells grow with a division and death rate of *r*_*2*_(1-*y*_*i*_*/MAI*_*i*_) and *d*_2_ per unit time, respectively. When the total number of all tumor cells reaches *M*_*diag*_, the tumor is detected and treatment in the form of chemotherapy, radiation, and/or surgery initiates. When the total number of cells reaches *M*_*death*_, the patient dies. See **S1(B)–S1(D) Doc** for a description of the model.
(TIF)

S2 Fig The model accurately predicts OS observed in the clinical cohort. The panel shows overall survival of patients in the MGH cohort (black line) and simulated cases (red line). Parameter values are *u* = 6.31×10^−5^, *q* = 6.31×10^−7^, *r*_*0*_ *and r*_*1*_ = 0.28, *r*_*2*_ = 1.16, death rate of each type = 1/100×growth rate, *M*_*diag*_ = 10^N(9.47,0.29)^, and *M*_*death*_ = 10^10.6^. LAI/MAI and growth rates during CTx for primary and metastatic sites are based on the estimated distributions (**S2 and S3 Data**), and *ε* was randomly chosen from [10^−5^, 10^−1^]. The number of simulated cases was the same as the number of patients in the clinical cohort (n = 1,089).
(TIF)

S3 Fig Subgroup analysis of the in silico clinical trial of CRTx for LAPC patients (Subgroup analysis of cases in Fig 4A). (A) Description of CRTx schedules for LAPC cases. (B and C) Assessment of survival of regimens 1 and 2 in simulated cases with LAPC (B) whose LAI is less than the median, and (C) whose LAI is larger than the median. The median LAI = 1.2×10^10^, 1.4×10^10^, and 1.4×10^10^ for FFX, GEM, and GEM+nab-paclitaxel, respectively. Number of simulated cases was 50 per group; and P-values by log-rank test were 0.117, 0.809, and 0.466 for FFX, GEM, and GEM+nab-paclitaxel in (B); and 0.0547, 0.0429, and 0.0379 for FFX, GEM, and GEM+nab-paclitaxel in (C). Parameter values were the same as those in **S2 Fig**.
(TIF)

S4 Fig Effects of CRTx schedules on PDAC progression phenotype at death. (A–C) Assessment of (A) the primary tumor size at death; (B) the average size of metastatic sites at death; and (C) the number of metastases at death with different chemotherapies in each regimen. Number of simulated cases was 100 per category, and P<0.001 for each pair of regimens using any drug in (A)–(C). Parameter values used for the panels were the same as those described in **S2 Fig**.
(TIF)

S5 Fig Sensitivity analysis of the effect of FFX under the assumption that treatment reversibly alters growth rates. (A) Description of tested CTx schedules with regard to our sensitivity analyses of the assumption of growth rate reversibility after treatment. In scenarios 2–4, the time until the growth rates recover to pre-treatment levels after treatment discontinuation is 2 weeks, 1 month, and 2 months, respectively. In scenarios 5 and 6, the levels the growth rate recovers to after treatment discontinuation are 95% and 90% of the original growth rate levels, respectively. (B) Waterfall plot of relative changes in diameters of simulated primary tumors at death compared with those at diagnosis in each scenario. P>0.05 for comparisons of all pairs of scenarios. (C) Assessment of survival duration. P>0.05 for comparisons of all pairs of scenarios 1–4; P>0.05 for scenarios 1 vs 5 and 1 vs 6. (D-F) Assessment of (D) the primary tumor size at death; (E) the average size of metastatic sites at death; and (F) the number of metastases at death in each scenario. P>0.05 for each pair of scenarios. Number of simulation cases are 100 per each scenario. Parameter values used are the same as those described in **S2 Fig**.
(TIF)

S1 Table Comparison of model fitting according to AIC. The Akaike Information Criterion is shown to assess model fits to the data. The models include the logistic model, the linear model, and the exponential model.
(DOCX)

S2 Table Median, 1^st^ and 3^rd^ quartiles for estimated growth rates and LAI/MAI using the logistic growth model. Estimated growth rates during CTx and in the absence of treatment are summarized and shown in the values of median, first and third quartiles. Also, the P-values by Mann-Whitney test are shown for the comparison of metastatic growth rates among the treatments.
(DOCX)

S3 Table P-values using the log-rank test for comparisons of the four chemotherapies regimens. The log-rank test was performed for the group comparisons regarding the Kaplan-Meier analyses shown in Fig 4F.
(DOCX)

S4 Table P-values using the log-rank test for comparisons of the three regimens with cessations. The log-rank test was performed for the group comparison regarding the Kaplan-Meier analyses shown in Fig 4G.
(DOCX)
