# Supplementary material for: Computational modeling of pancreatic cancer patients receiving FOLFIRINOX and gemcitabine-based therapies identifies optimum intervention strategies
Source: PLoS One. 2019 Apr 26;14(4):e0215409. doi: 10.1371/journal.pone.0215409 (PMC6485645; doi:10.1371/journal.pone.0215409)
Supplement: S2 Data — The estimated growth curves with the logistic model (solid line) and the exponential model (dotted line) for each patient are shown and the tumor sizes at the timing of each medical examination in the clinical data are also plotted as red square. (PDF) [file pone.0215409.s002.pdf]

Primary no treatment

Y axis: Tumor volume/10^2 (cm^3)

X axis: Months

line: logistic model

dotted line: exponential model

red square: data

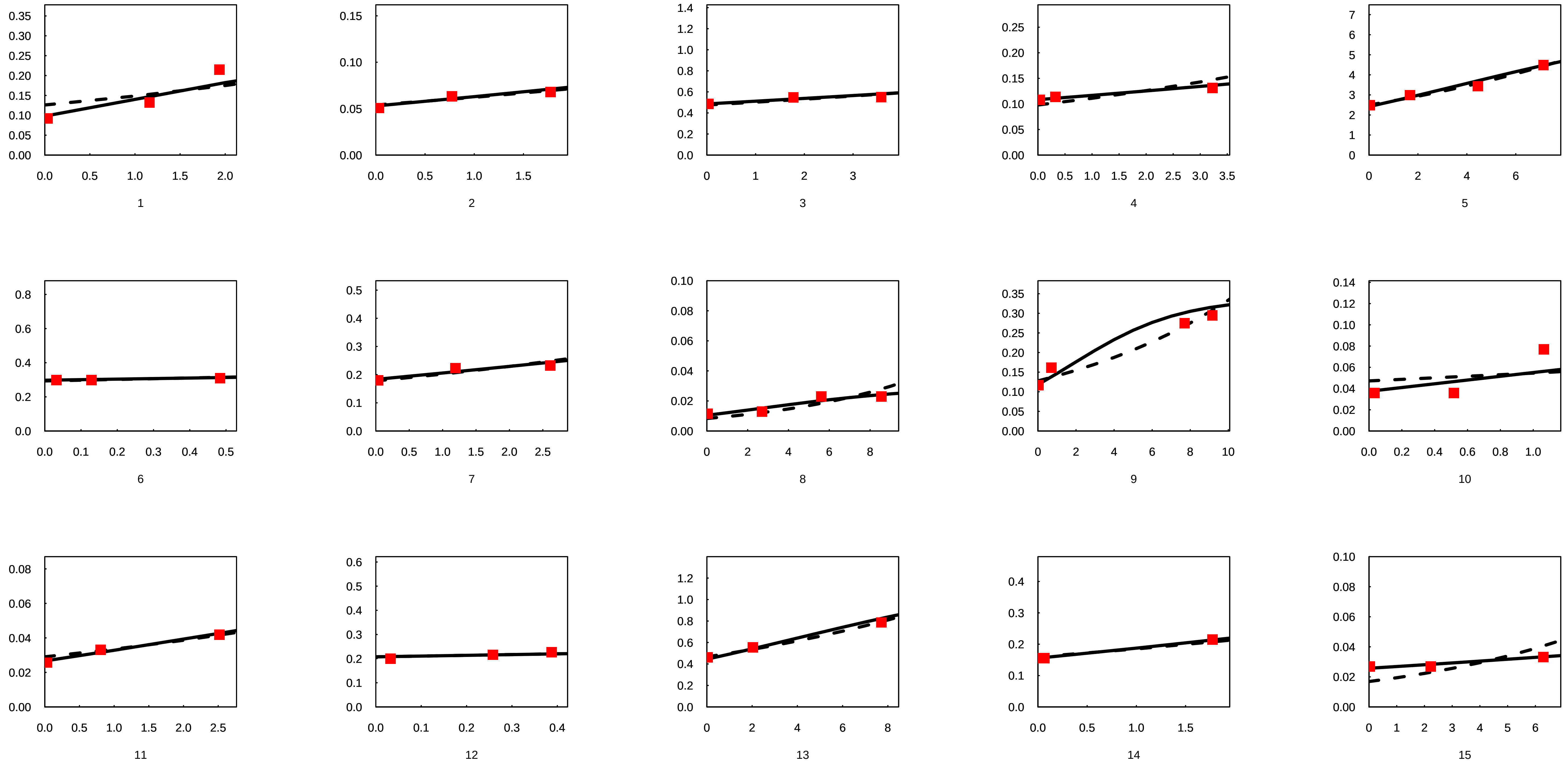

0.35 0.30 0.25 0.20 0.15 0.10 0.05 0.00
0.0 0.5 1.0 1.5 2.0
1
0.15 0.10 0.05 0.00
0.0 0.5 1.0 1.5
2
1.4 1.2 1.0 0.8 0.6 0.4 0.2 0.0
0 1 2 3
3
0.25 0.20 0.15 0.10 0.05 0.00
0.0 0.5 1.0 1.5 2.0 2.5 3.0 3.5
4
7 6 5 4 3 2 1 0
0 2 4 6
5
0.8 0.6 0.4 0.2 0.0
0.0 0.1 0.2 0.3 0.4 0.5
6
0.5 0.4 0.3 0.2 0.1 0.0
0.0 0.5 1.0 1.5 2.0 2.5
7
0.10 0.08 0.06 0.04 0.02 0.00
0 2 4 6 8
8
0.35 0.30 0.25 0.20 0.15 0.10 0.05 0.00
0 2 4 6 8 10
9
0.14 0.12 0.10 0.08 0.06 0.04 0.02 0.00
0.0 0.2 0.4 0.6 0.8 1.0
10
0.08 0.06 0.04 0.02 0.00
0.0 0.5 1.0 1.5 2.0 2.5
11
0.6 0.5 0.4 0.3 0.2 0.1 0.0
0.0 0.1 0.2 0.3 0.4
12
1.2 1.0 0.8 0.6 0.4 0.2 0.0
0 2 4 6 8
13
0.4 0.3 0.2 0.1 0.0
0.0 0.5 1.0 1.5
14
0.10 0.08 0.06 0.04 0.02 0.00
0 1 2 3 4 5 6
15

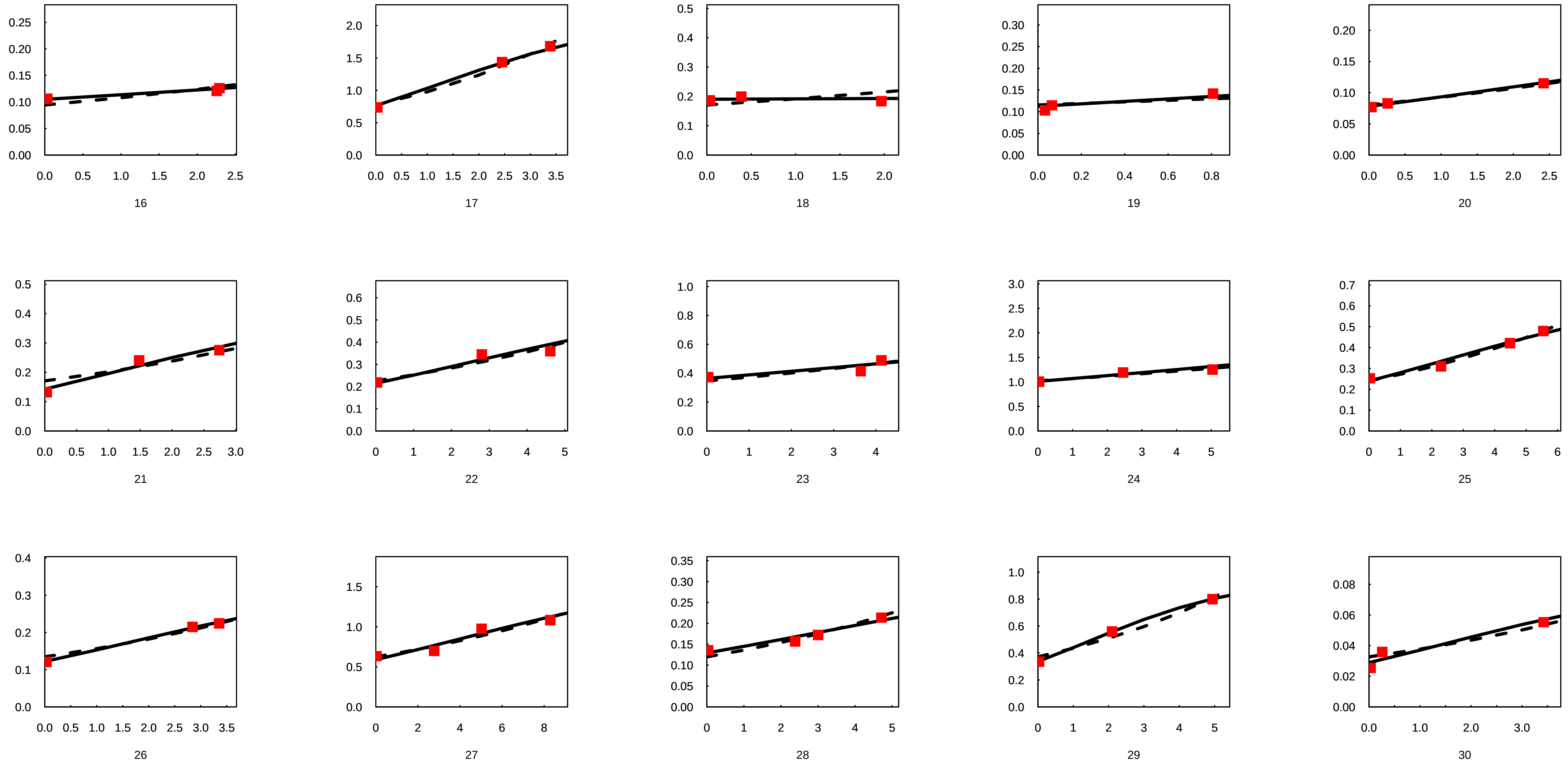
16
17
18
19
20
21
22
23
24
25
26
27
28
29
30

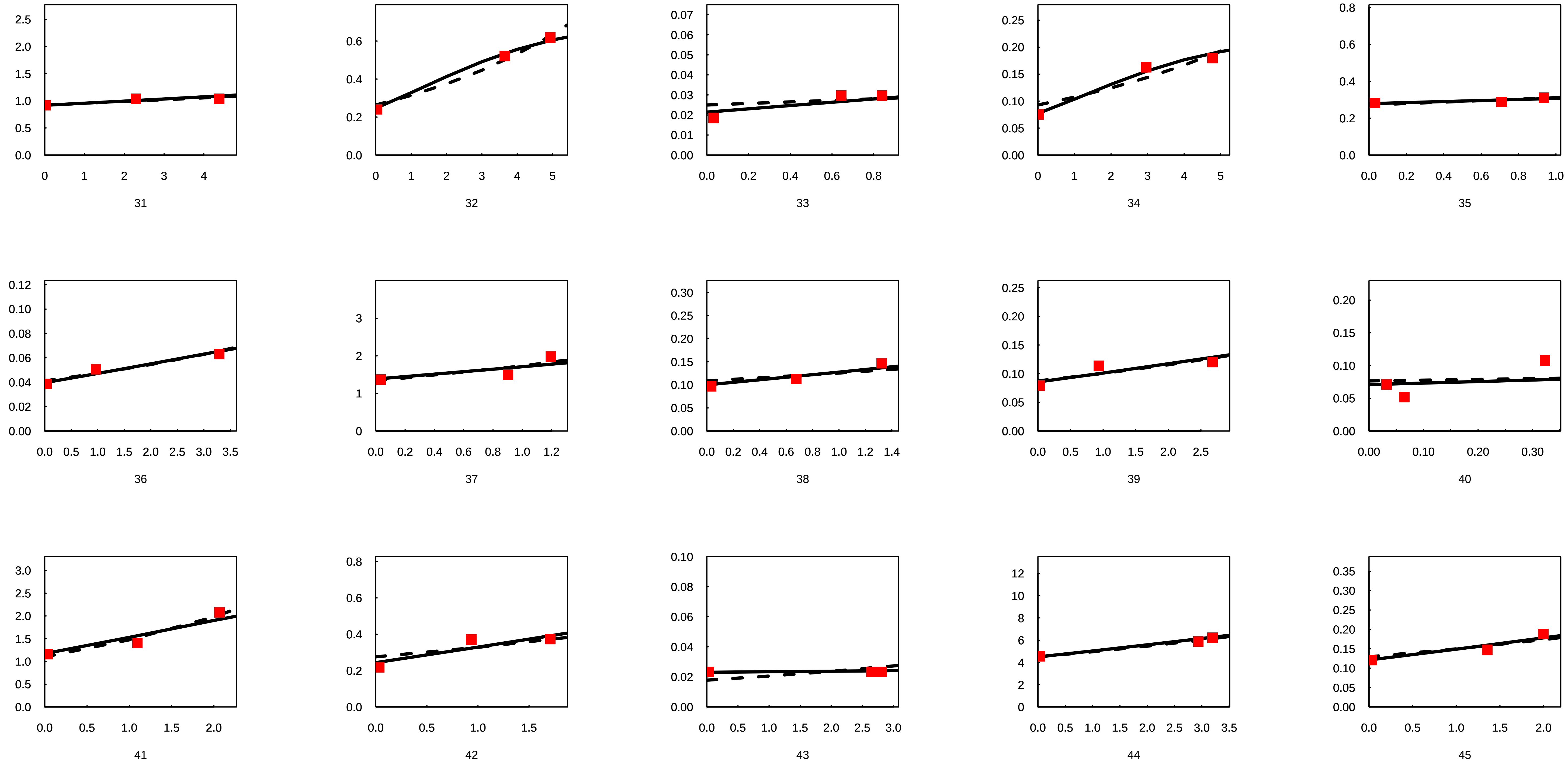
31
32
33
34
35
36
37
38
39
40
41
42
43
44
45

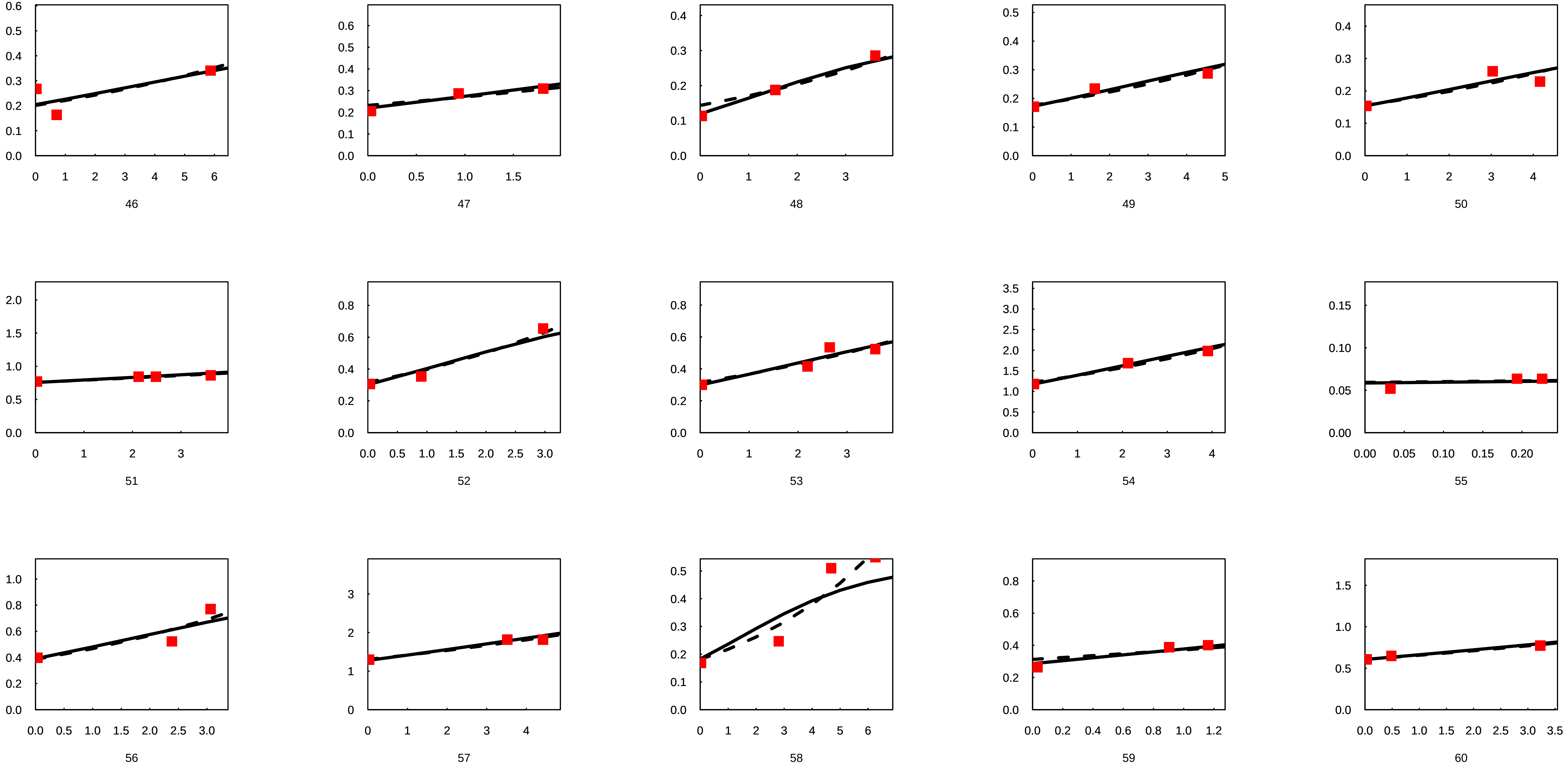
46
47
48
49
50
51
52
53
54
55
56
57
58
59
60

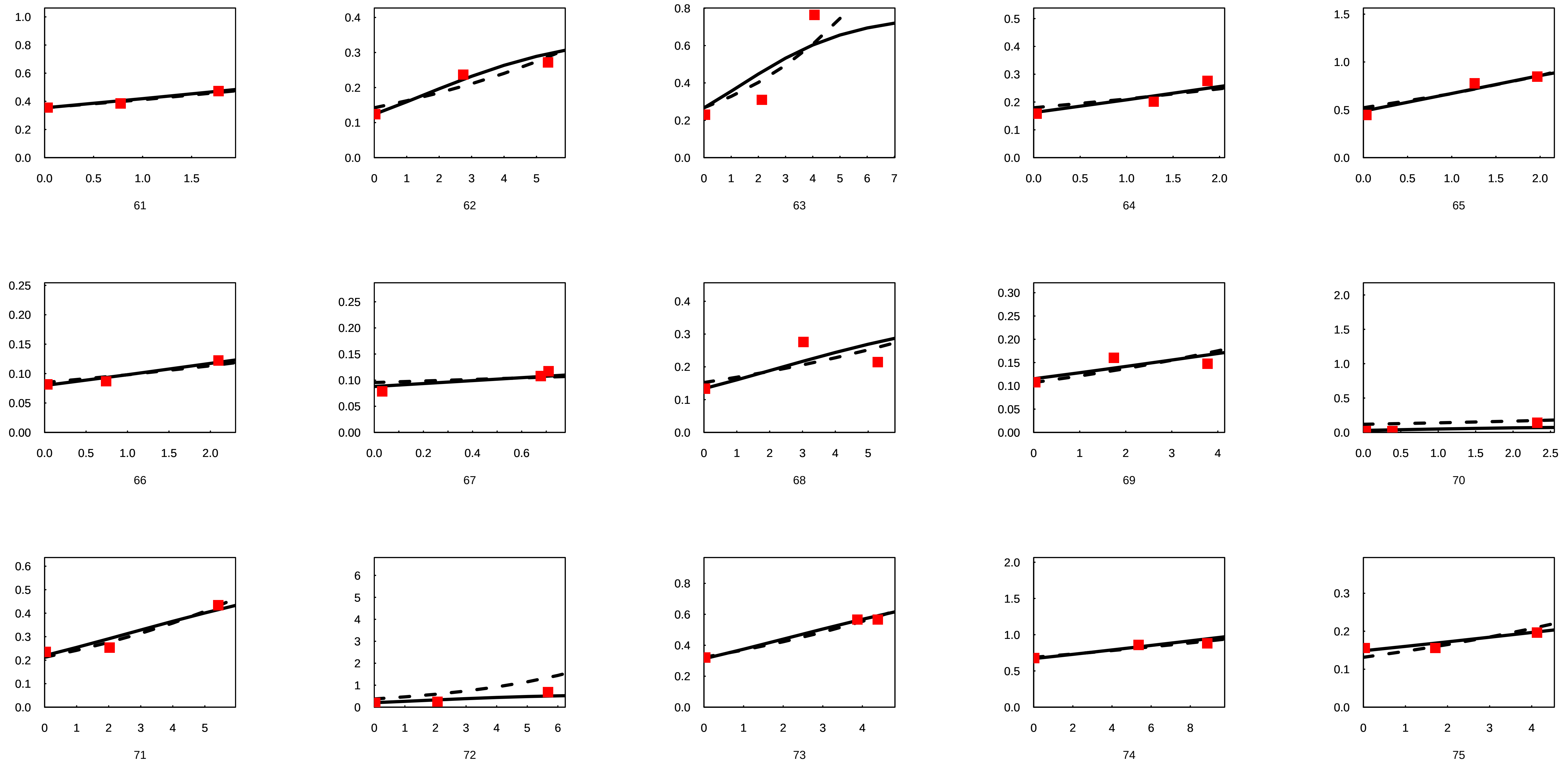
61
62
63
64
65
66
67
68
69
70
71
72
73
74
75

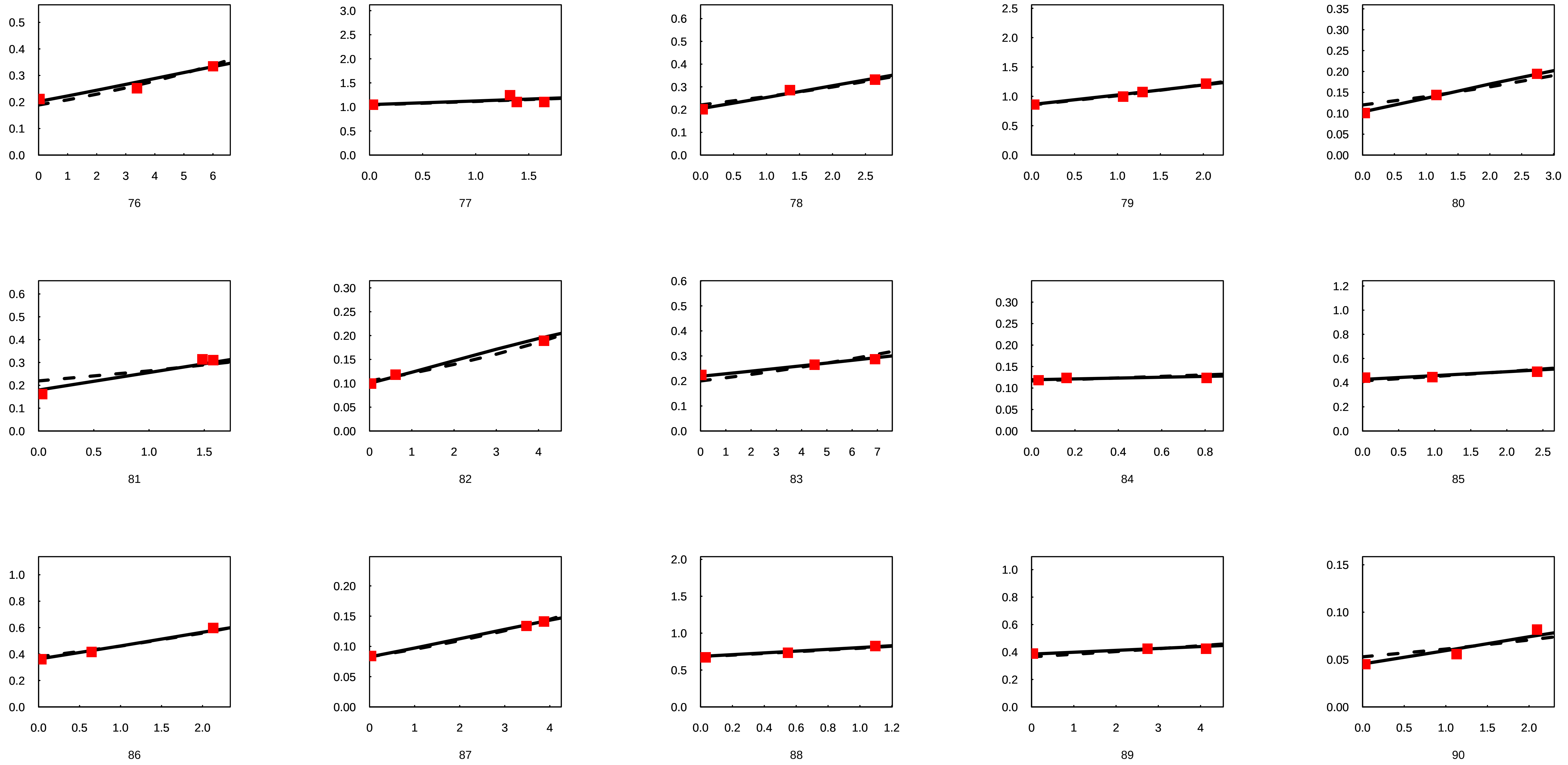
0.0 0.1 0.2 0.3 0.4 0.5
0 1 2 3 4 5 6
76
0.0 0.5 1.0 1.5 2.0 2.5 3.0
0.0 0.5 1.0 1.5
77
0.0 0.1 0.2 0.3 0.4 0.5 0.6
0.0 0.5 1.0 1.5 2.0 2.5
78
0.0 0.5 1.0 1.5 2.0 2.5
0.0 0.5 1.0 1.5 2.0
79
0.00 0.05 0.10 0.15 0.20 0.25 0.30 0.35
0.0 0.5 1.0 1.5 2.0 2.5 3.0
80
0.0 0.1 0.2 0.3 0.4 0.5 0.6
0.0 0.5 1.0 1.5
81
0.00 0.05 0.10 0.15 0.20 0.25 0.30
0 1 2 3 4
82
0.0 0.1 0.2 0.3 0.4 0.5 0.6
0 1 2 3 4 5 6 7
83
0.00 0.05 0.10 0.15 0.20 0.25 0.30
0.0 0.2 0.4 0.6 0.8
84
0.0 0.2 0.4 0.6 0.8 1.0 1.2
0.0 0.5 1.0 1.5 2.0 2.5
85
0.0 0.2 0.4 0.6 0.8 1.0
0.0 0.5 1.0 1.5 2.0
86
0.00 0.05 0.10 0.15 0.20
0 1 2 3 4
87
0.0 0.5 1.0 1.5 2.0
0.0 0.2 0.4 0.6 0.8 1.0 1.2
88
0.0 0.2 0.4 0.6 0.8 1.0
0 1 2 3 4
89
0.00 0.05 0.10 0.15
0.0 0.5 1.0 1.5 2.0
90

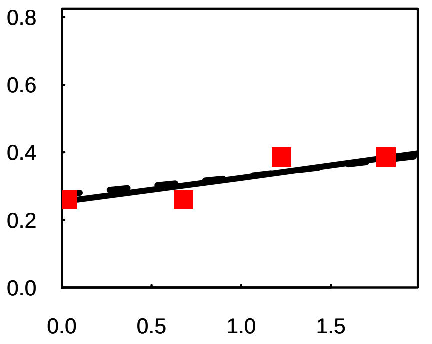
0.8
0.6
0.4
0.2
0.0
0.0
0.5
1.0
1.5

91

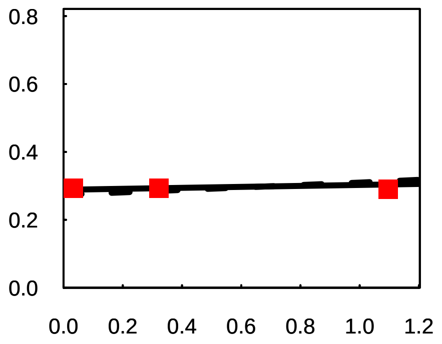
0.8
0.6
0.4
0.2
0.0
0.0
0.2
0.4
0.6
0.8
1.0
1.2

92

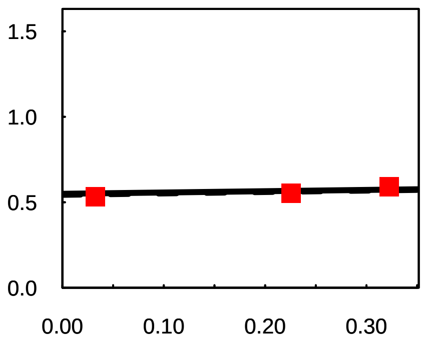
1.5
1.0
0.5
0.0
0.00
0.10
0.20
0.30

93

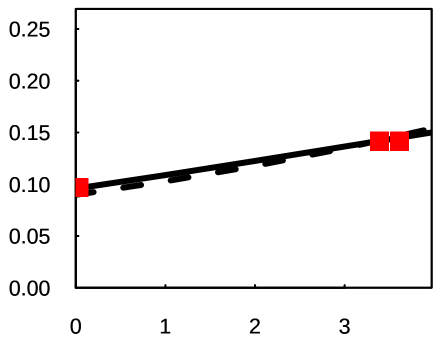
0.25
0.20
0.15
0.10
0.05
0.00
0
1
2
3

94

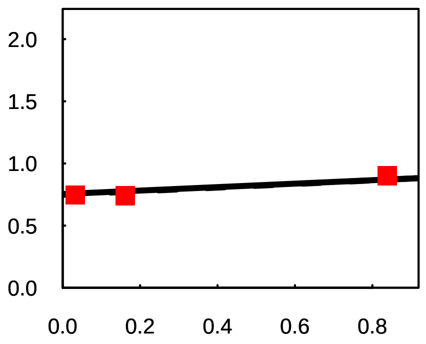
2.0
1.5
1.0
0.5
0.0
0.0
0.2
0.4
0.6
0.8

95

Metastasis no treatment

Y axis: Tumor volume/10^2 (cm^3)

X axis: Months

line: logistic model

dotted line: exponential model

red square: data

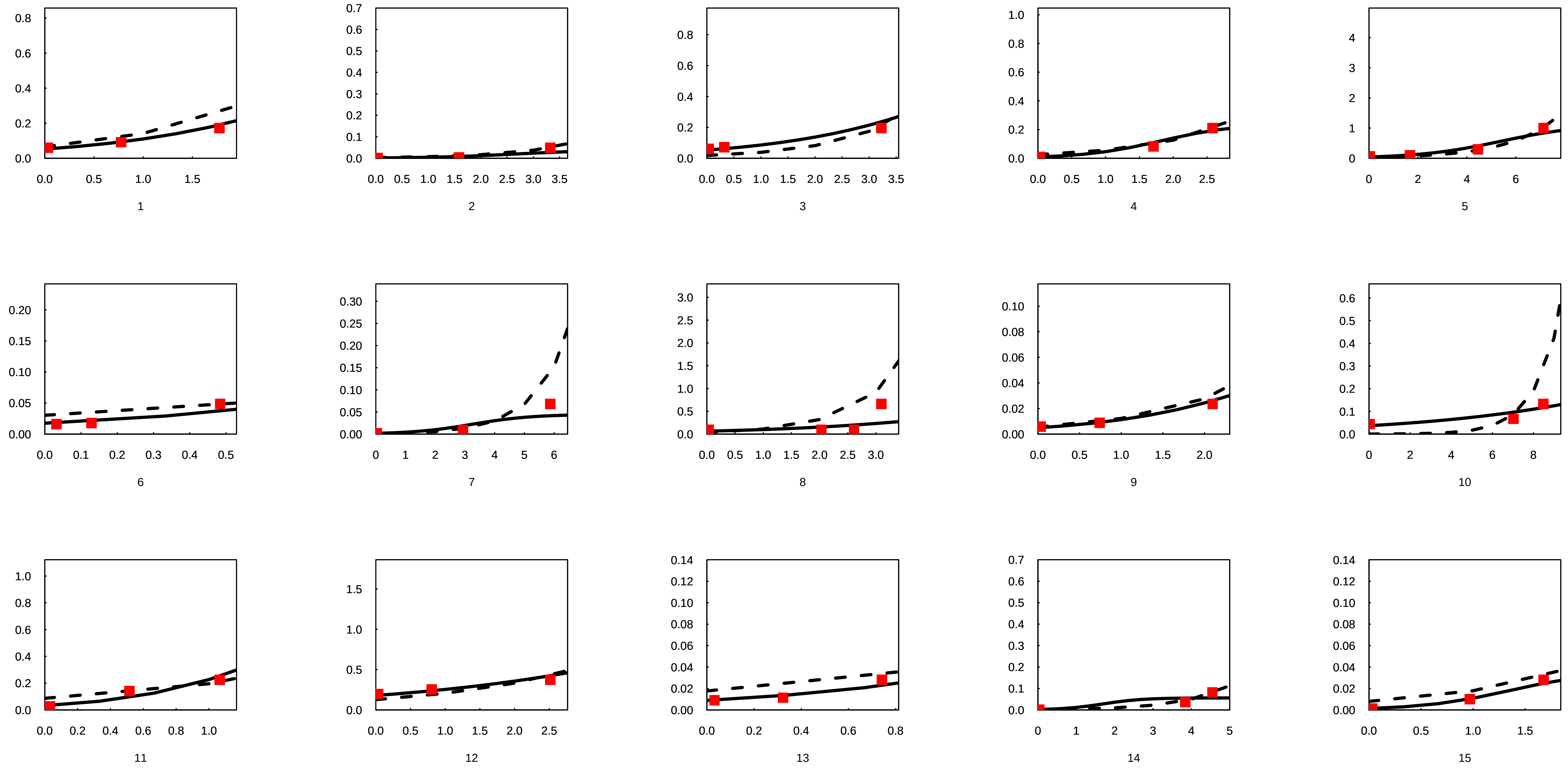
1
2
3
4
5
6
7
8
9
10
11
12
13
14
15

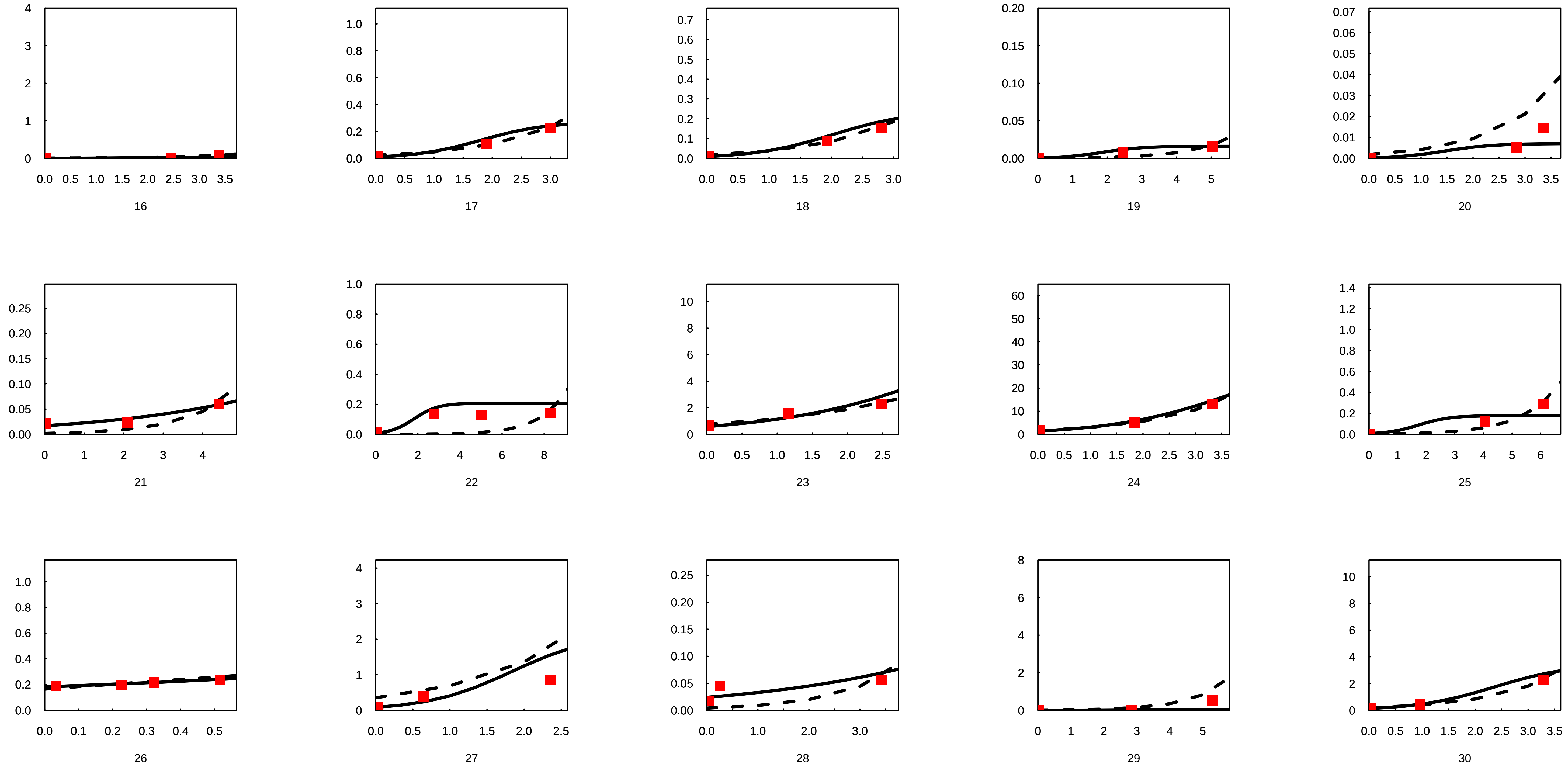
16
17
18
19
20
21
22
23
24
25
26
27
28
29
30

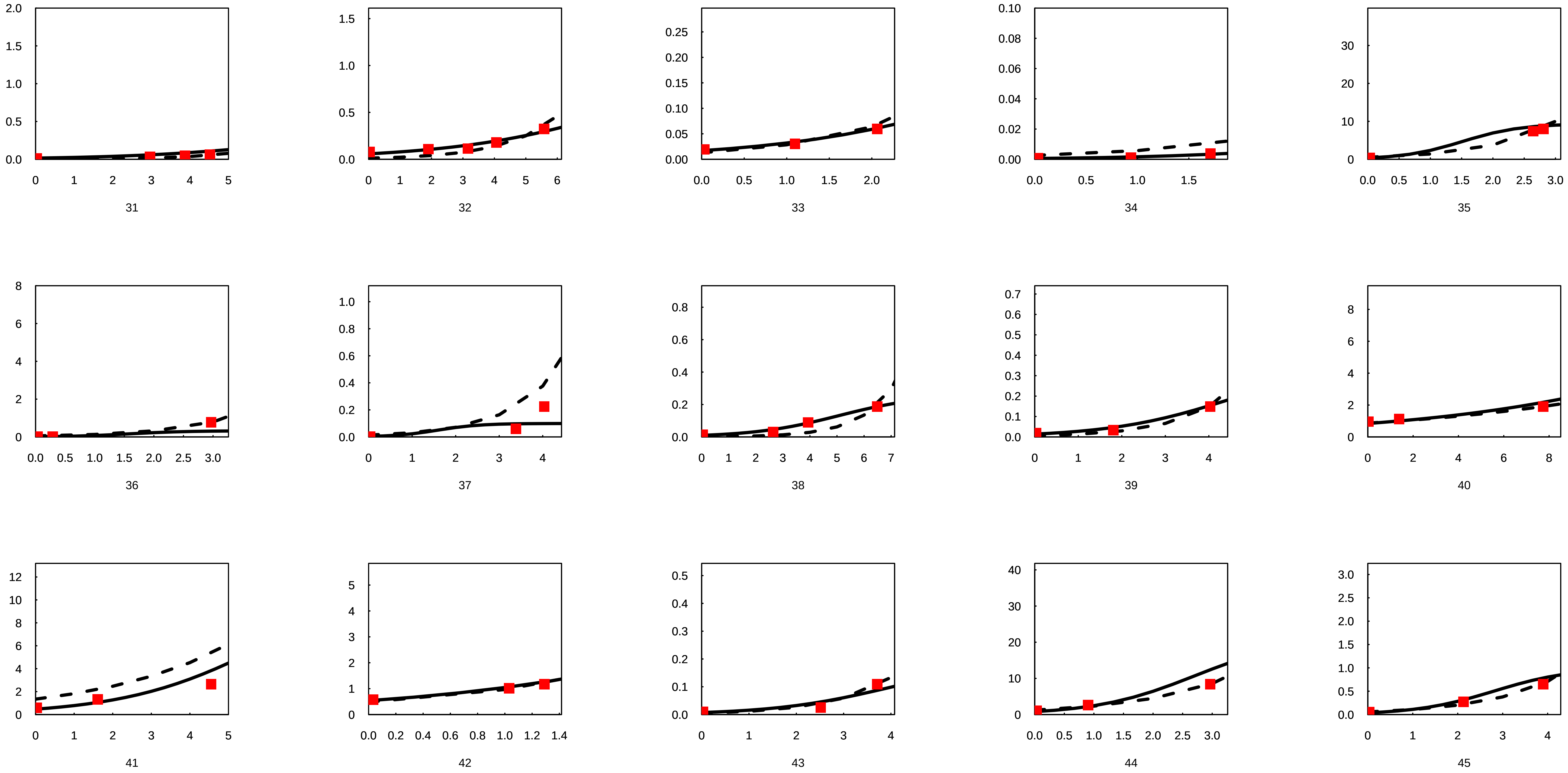
31
32
33
34
35
36
37
38
39
40
41
42
43
44
45

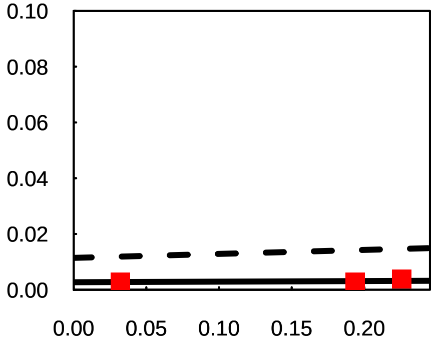

46

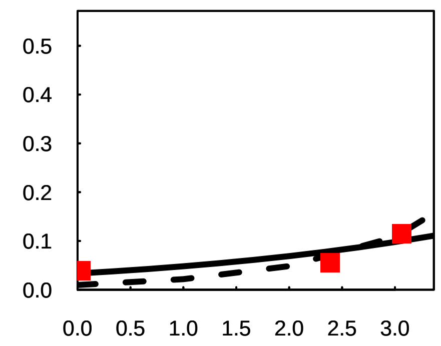

47

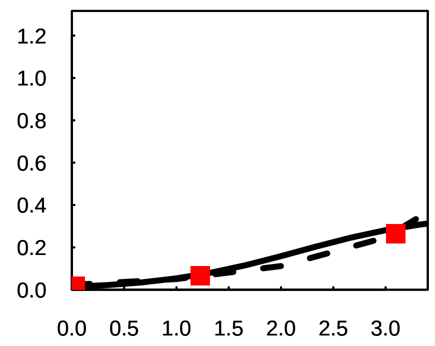

48

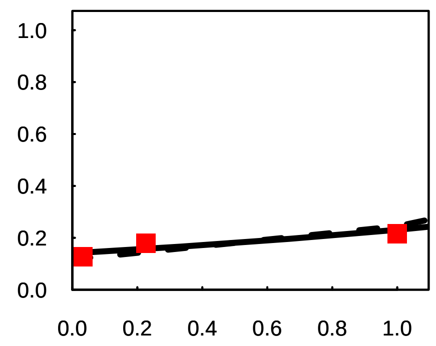

49

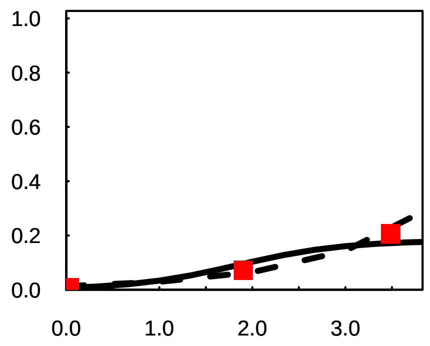

50

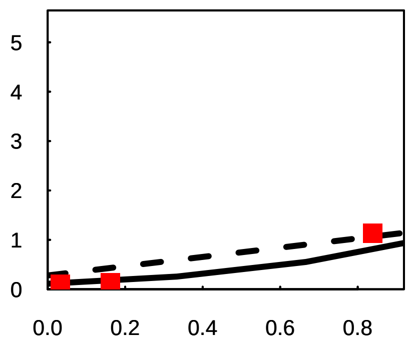

51

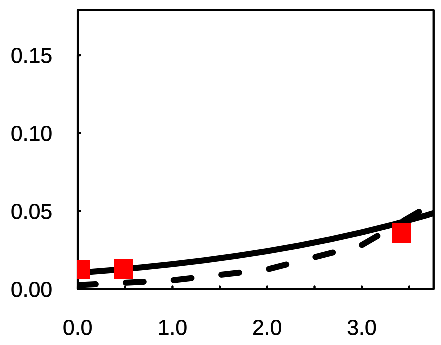

52

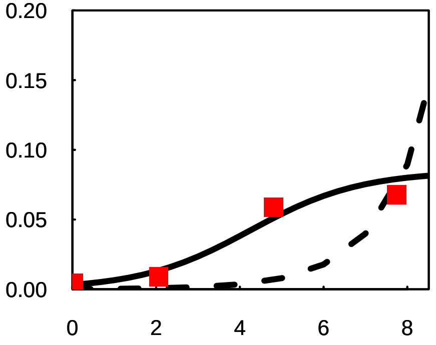

53

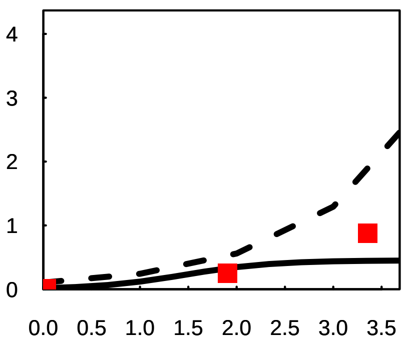

54

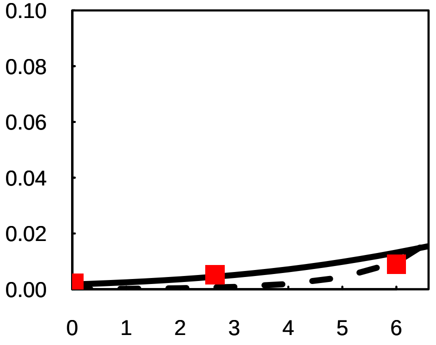

55

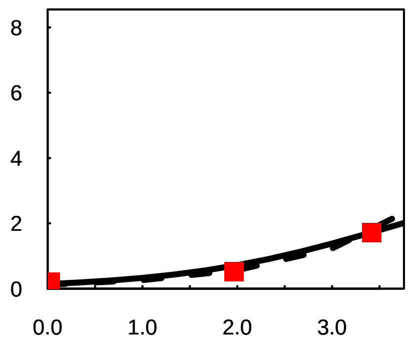

56

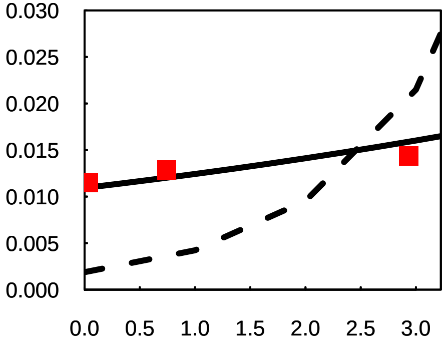

57

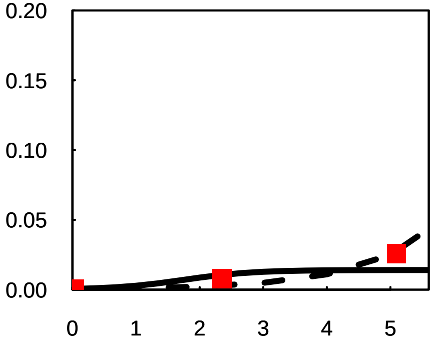

58

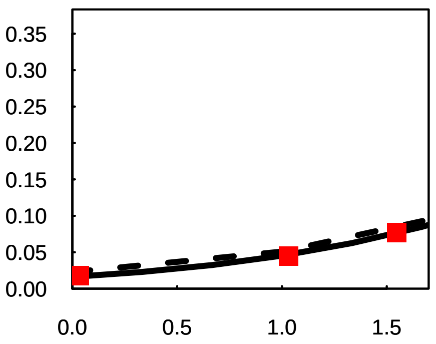

59

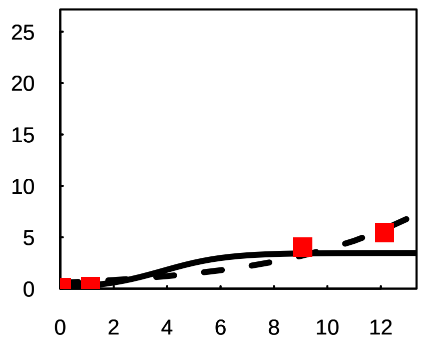

60

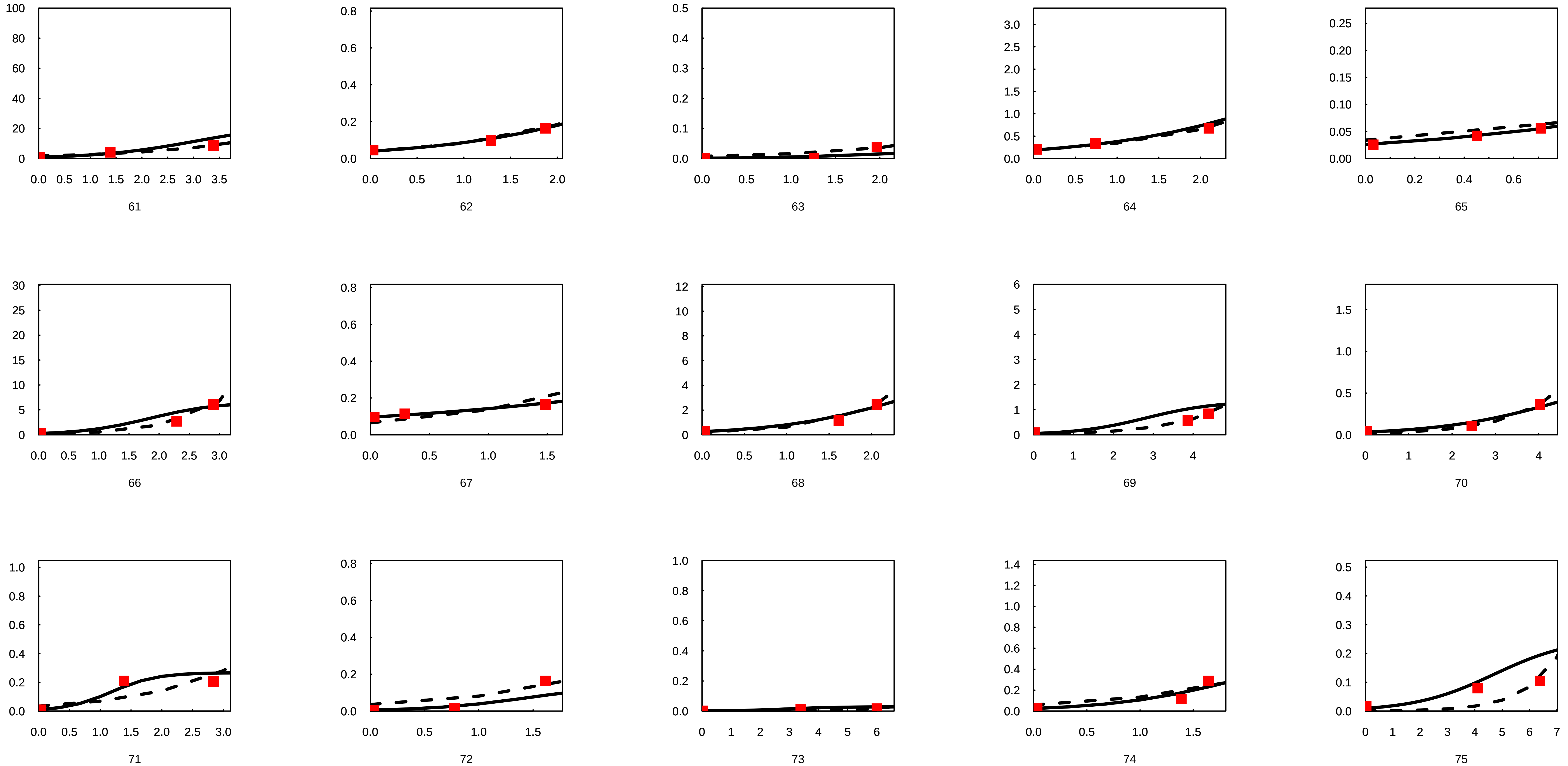
61
62
63
64
65
66
67
68
69
70
71
72
73
74
75

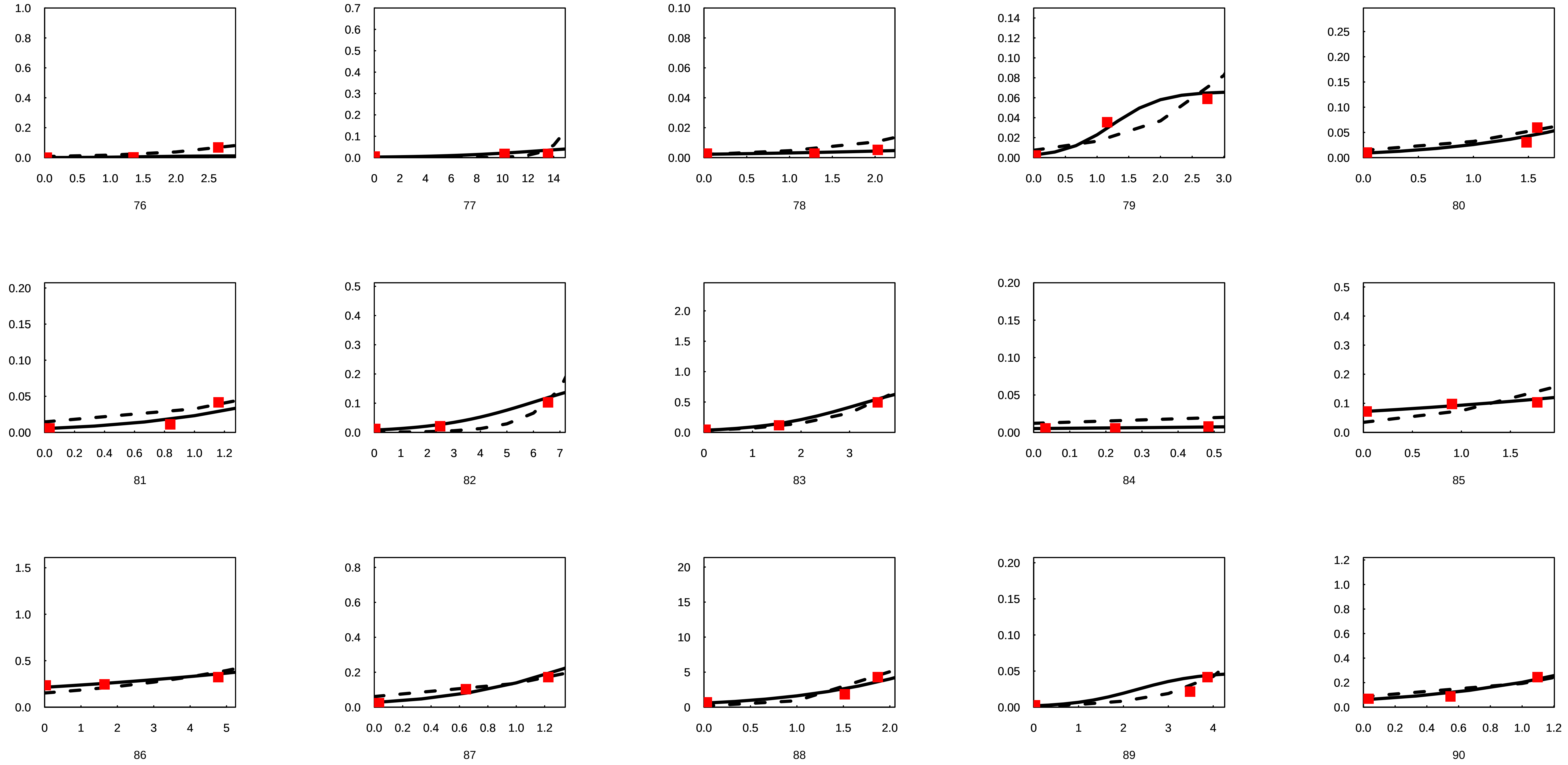

1.0 0.8 0.6 0.4 0.2 0.0
0.0 0.5 1.0 1.5 2.0 2.5
76
0.7 0.6 0.5 0.4 0.3 0.2 0.1 0.0
0 2 4 6 8 10 12 14
77
0.10 0.08 0.06 0.04 0.02 0.00
0.0 0.5 1.0 1.5 2.0
78
0.14 0.12 0.10 0.08 0.06 0.04 0.02 0.00
0.0 0.5 1.0 1.5 2.0 2.5 3.0
79
0.25 0.20 0.15 0.10 0.05 0.00
0.0 0.5 1.0 1.5
80
0.20 0.15 0.10 0.05 0.00
0.0 0.2 0.4 0.6 0.8 1.0 1.2
81
0.5 0.4 0.3 0.2 0.1 0.0
0 1 2 3 4 5 6 7
82
2.0 1.5 1.0 0.5 0.0
0 1 2 3
83
0.20 0.15 0.10 0.05 0.00
0.0 0.1 0.2 0.3 0.4 0.5
84
0.5 0.4 0.3 0.2 0.1 0.0
0.0 0.5 1.0 1.5
85
1.5 1.0 0.5 0.0
0 1 2 3 4 5
86
0.8 0.6 0.4 0.2 0.0
0.0 0.2 0.4 0.6 0.8 1.0 1.2
87
20 15 10 5 0
0.0 0.5 1.0 1.5 2.0
88
0.20 0.15 0.10 0.05 0.00
0 1 2 3 4
89
1.2 1.0 0.8 0.6 0.4 0.2 0.0
0.0 0.2 0.4 0.6 0.8 1.0 1.2
90

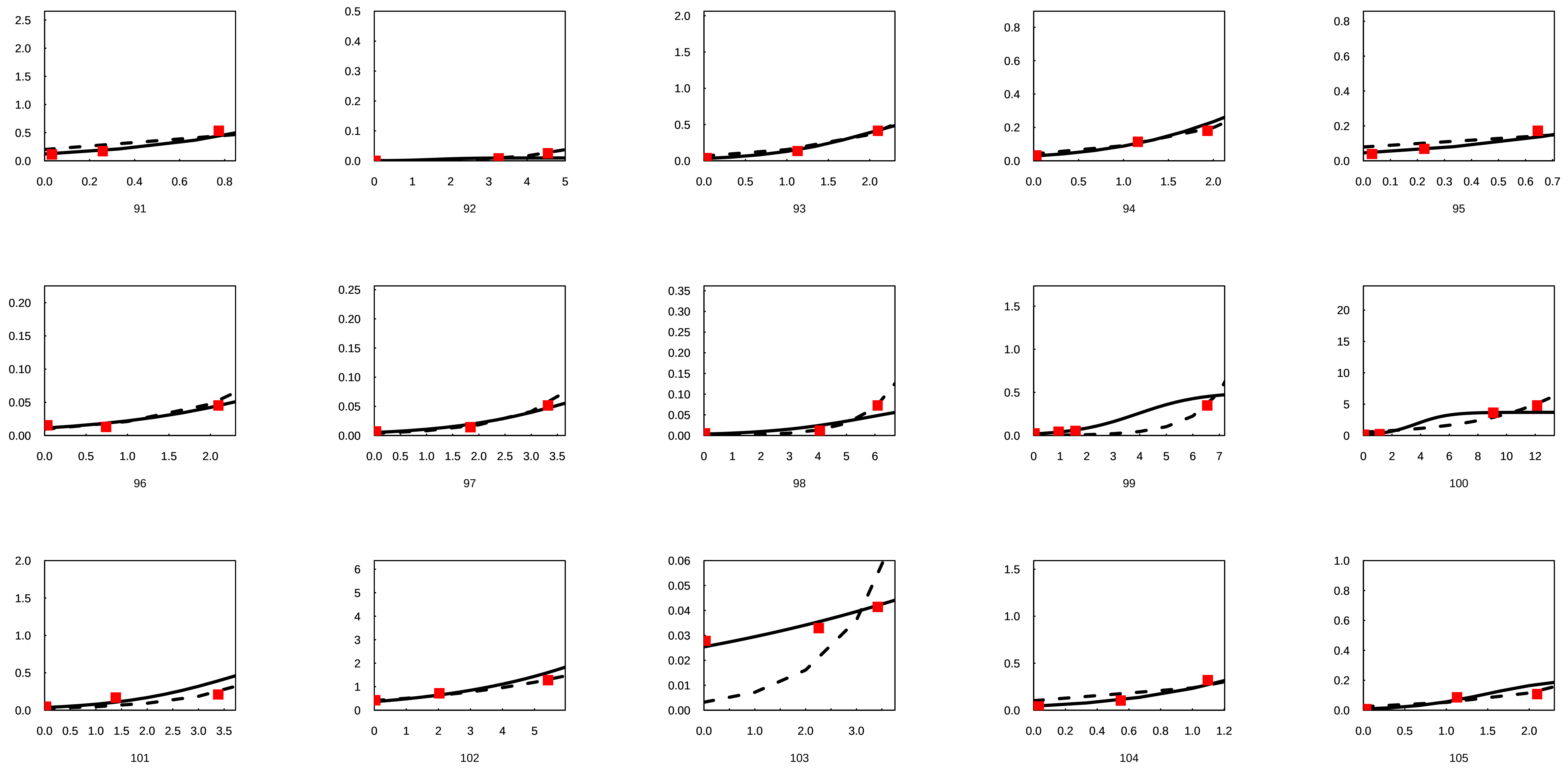
91
92
93
94
95
96
97
98
99
100
101
102
103
104
105

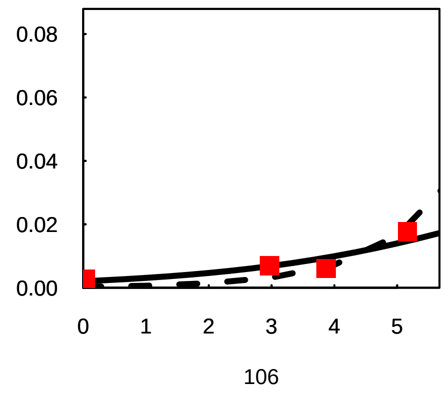
0.08
0.06
0.04
0.02
0.00
0
1
2
3
4
5
106

Primary gemcitabine

Y axis: Tumor volume/10^2 (cm^3)

X axis: Months

line: logistic model

dotted line: exponential model

red square: data

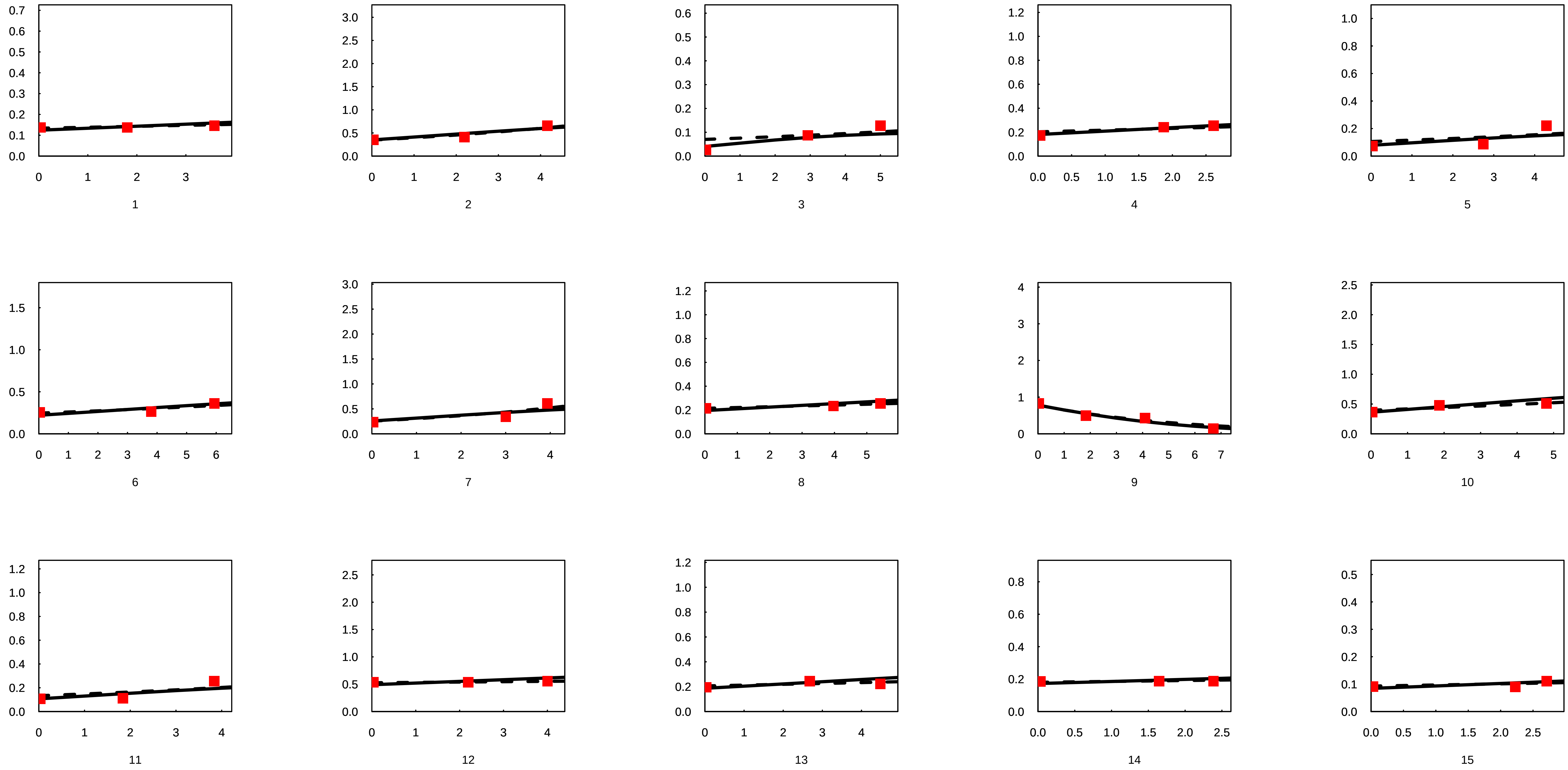
0.7 0.6 0.5 0.4 0.3 0.2 0.1 0.0
0 1 2 3
1
3.0 2.5 2.0 1.5 1.0 0.5 0.0
0 1 2 3 4
2
0.6 0.5 0.4 0.3 0.2 0.1 0.0
0 1 2 3 4 5
3
1.2 1.0 0.8 0.6 0.4 0.2 0.0
0.0 0.5 1.0 1.5 2.0 2.5
4
1.0 0.8 0.6 0.4 0.2 0.0
0 1 2 3 4
5
1.5 1.0 0.5 0.0
0 1 2 3 4 5 6
6
3.0 2.5 2.0 1.5 1.0 0.5 0.0
0 1 2 3 4
7
1.2 1.0 0.8 0.6 0.4 0.2 0.0
0 1 2 3 4 5
8
4 3 2 1 0
0 1 2 3 4 5 6 7
9
2.5 2.0 1.5 1.0 0.5 0.0
0 1 2 3 4 5
10
1.2 1.0 0.8 0.6 0.4 0.2 0.0
0 1 2 3 4
11
2.5 2.0 1.5 1.0 0.5 0.0
0 1 2 3 4
12
1.2 1.0 0.8 0.6 0.4 0.2 0.0
0 1 2 3 4
13
0.8 0.6 0.4 0.2 0.0
0.0 0.5 1.0 1.5 2.0 2.5
14
0.5 0.4 0.3 0.2 0.1 0.0
0.0 0.5 1.0 1.5 2.0 2.5
15

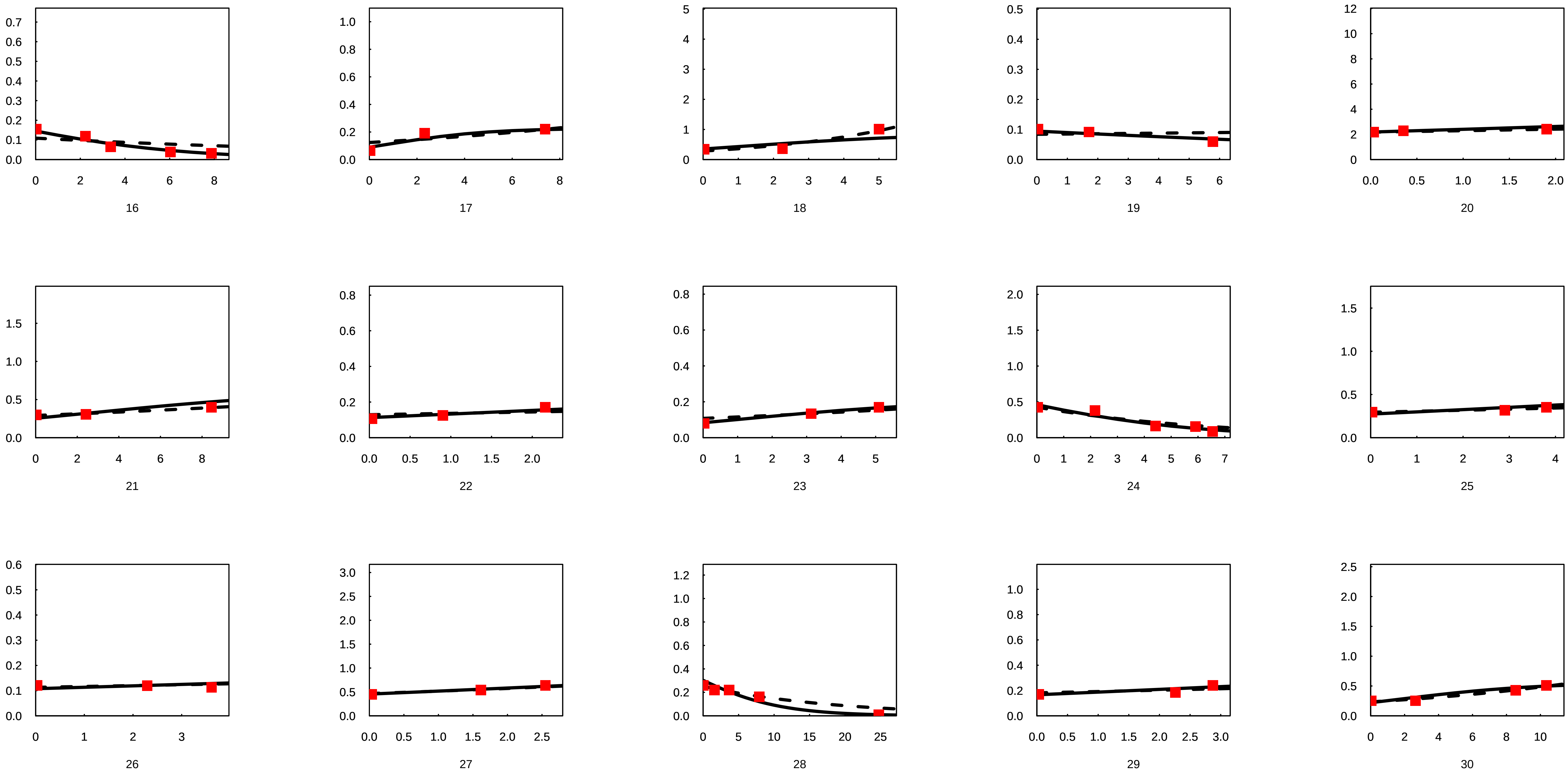
0.7 0.6 0.5 0.4 0.3 0.2 0.1 0.0
0 2 4 6 8
16
1.0 0.8 0.6 0.4 0.2 0.0
0 2 4 6 8
17
5 4 3 2 1 0
0 1 2 3 4 5
18
0.5 0.4 0.3 0.2 0.1 0.0
0 1 2 3 4 5 6
19
12 10 8 6 4 2 0
0.0 0.5 1.0 1.5 2.0
20
1.5 1.0 0.5 0.0
0 2 4 6 8
21
0.8 0.6 0.4 0.2 0.0
0.0 0.5 1.0 1.5 2.0
22
0.8 0.6 0.4 0.2 0.0
0 1 2 3 4 5
23
2.0 1.5 1.0 0.5 0.0
0 1 2 3 4 5 6 7
24
1.5 1.0 0.5 0.0
0 1 2 3 4
25
0.6 0.5 0.4 0.3 0.2 0.1 0.0
0 1 2 3
26
3.0 2.5 2.0 1.5 1.0 0.5 0.0
0.0 0.5 1.0 1.5 2.0 2.5
27
1.2 1.0 0.8 0.6 0.4 0.2 0.0
0 5 10 15 20 25
28
1.0 0.8 0.6 0.4 0.2 0.0
0.0 0.5 1.0 1.5 2.0 2.5 3.0
29
2.5 2.0 1.5 1.0 0.5 0.0
0 2 4 6 8 10
30

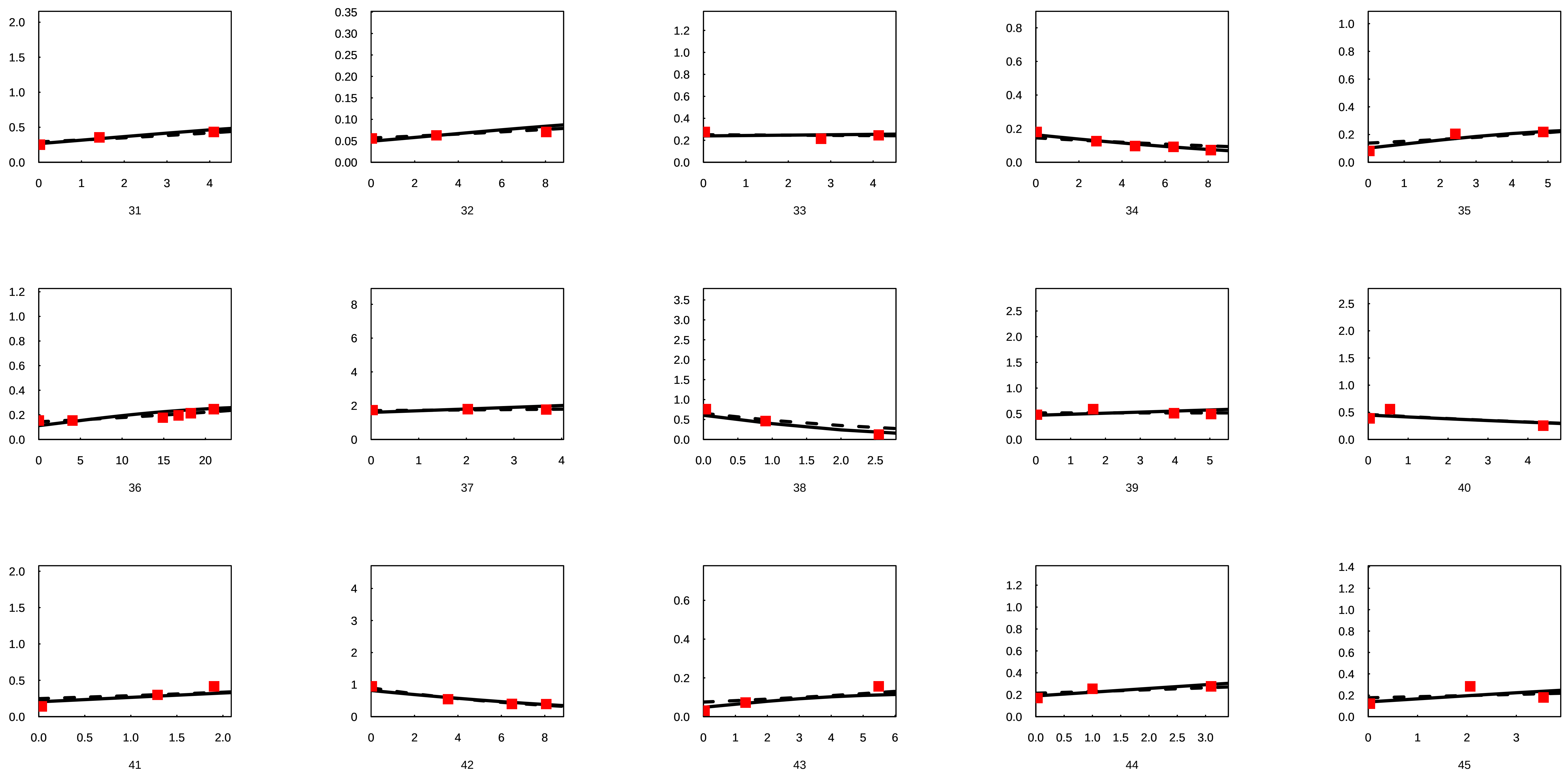
31
32
33
34
35
36
37
38
39
40
41
42
43
44
45

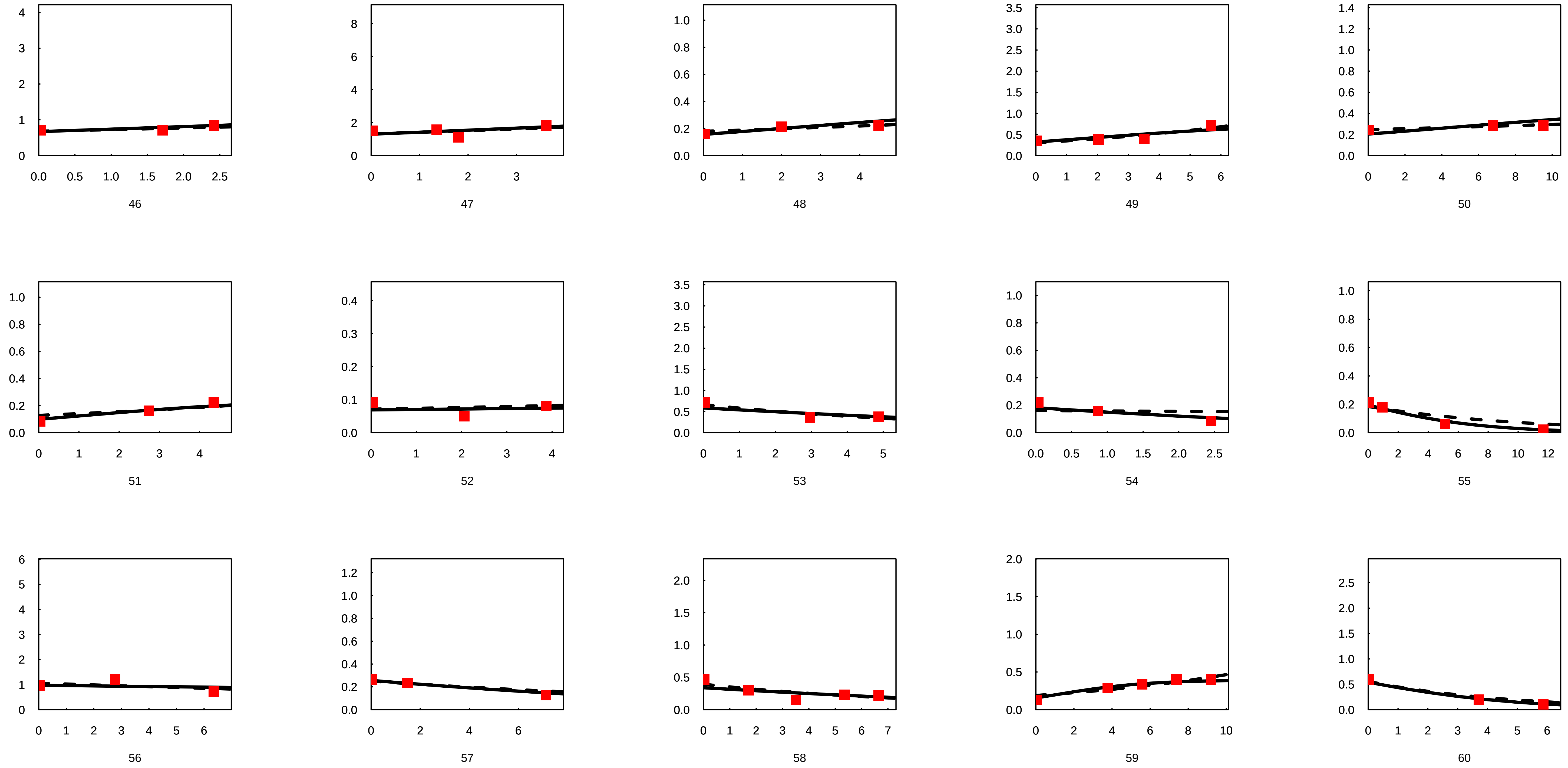

0 1 2 3 4
0.0 0.5 1.0 1.5 2.0 2.5
46
0 2 4 6 8
0 1 2 3
47
0.0 0.2 0.4 0.6 0.8 1.0
0 1 2 3 4
48
0.0 0.5 1.0 1.5 2.0 2.5 3.0 3.5
0 1 2 3 4 5 6
49
0.0 0.2 0.4 0.6 0.8 1.0 1.2 1.4
0 2 4 6 8 10
50
0.0 0.2 0.4 0.6 0.8 1.0
0 1 2 3 4
51
0.0 0.1 0.2 0.3 0.4
0 1 2 3 4
52
0.0 0.5 1.0 1.5 2.0 2.5 3.0 3.5
0 1 2 3 4 5
53
0.0 0.2 0.4 0.6 0.8 1.0
0.0 0.5 1.0 1.5 2.0 2.5
54
0.0 0.2 0.4 0.6 0.8 1.0
0 2 4 6 8 10 12
55
0 1 2 3 4 5 6
0 1 2 3 4 5 6
56
0.0 0.2 0.4 0.6 0.8 1.0 1.2
0 2 4 6
57
0.0 0.5 1.0 1.5 2.0
0 1 2 3 4 5 6 7
58
0.0 0.5 1.0 1.5 2.0
0 2 4 6 8 10
59
0.0 0.5 1.0 1.5 2.0 2.5
0 1 2 3 4 5 6
60

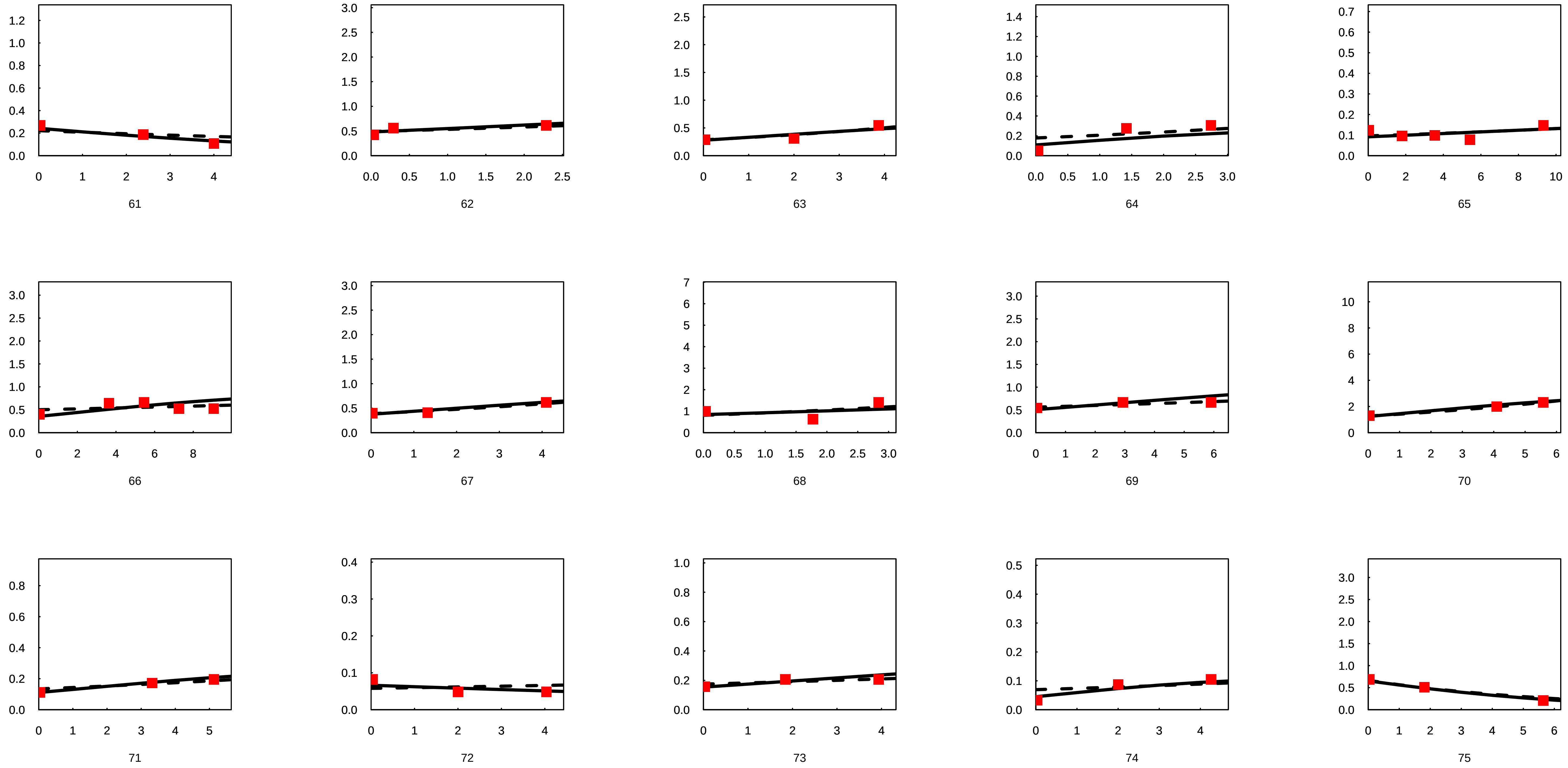
61
62
63
64
65
66
67
68
69
70
71
72
73
74
75

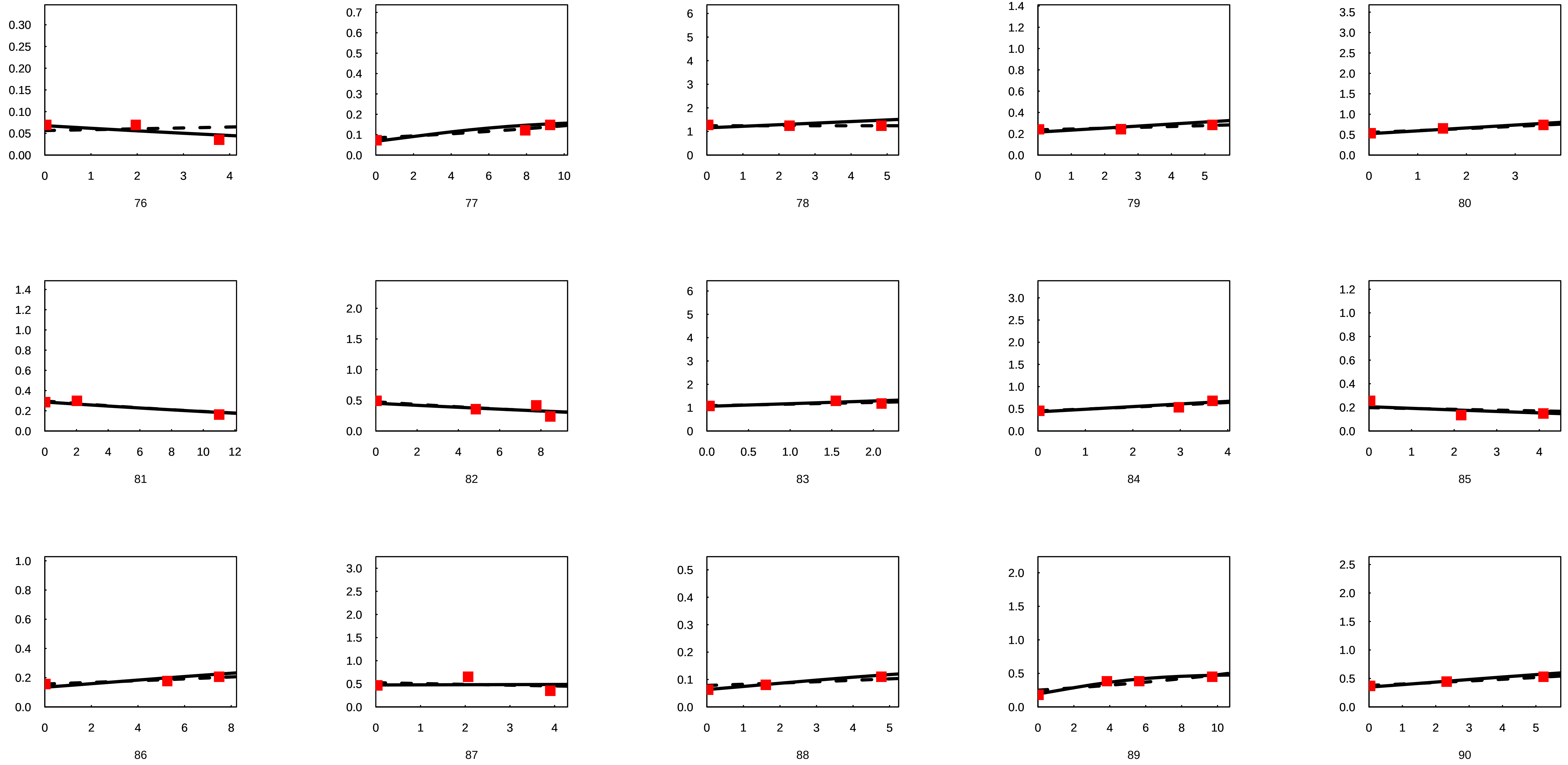

76
77
78
79
80
81
82
83
84
85
86
87
88
89
90

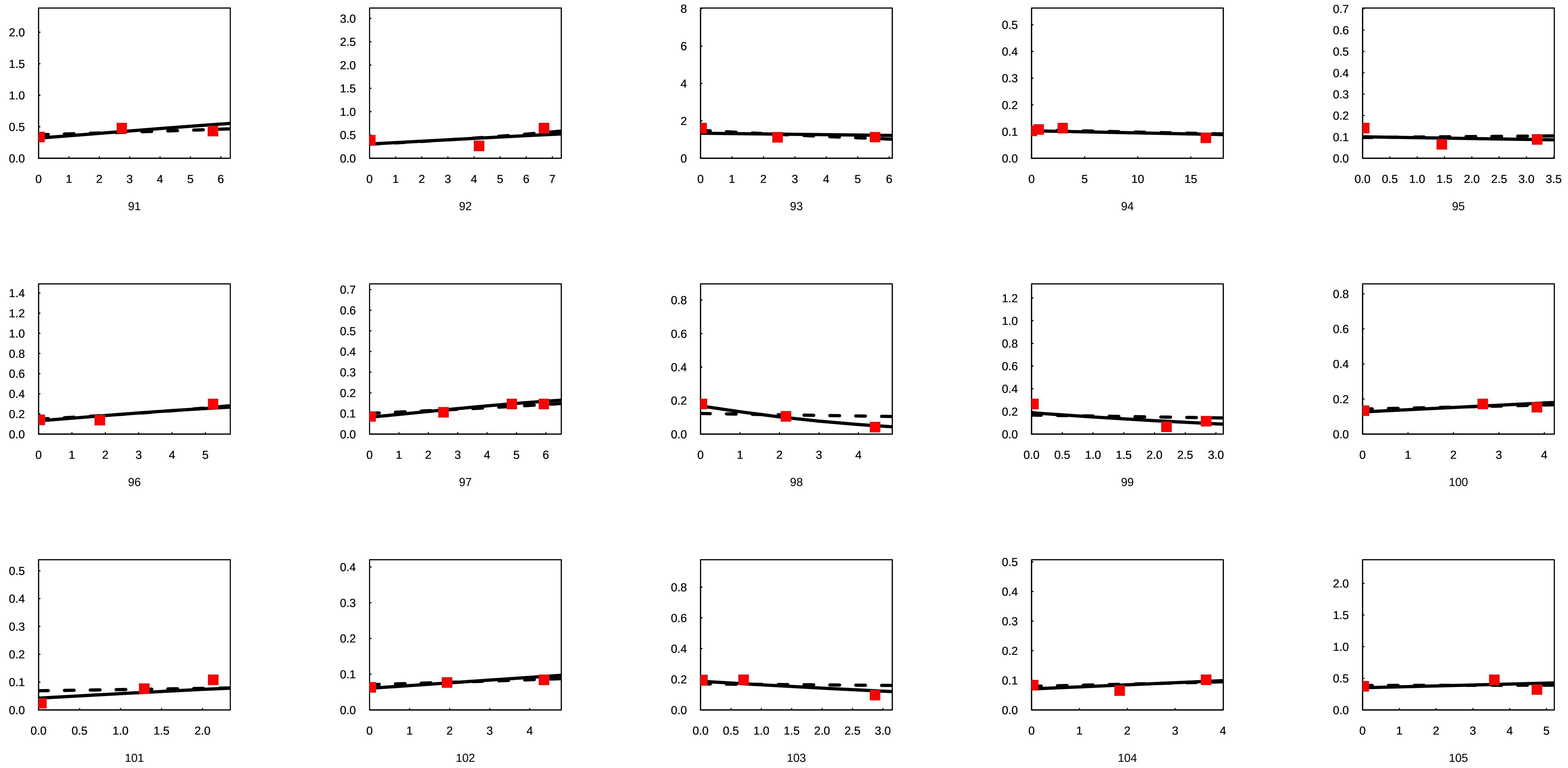
0.0 0.5 1.0 1.5 2.0
0 1 2 3 4 5 6
91
0.0 0.5 1.0 1.5 2.0 2.5 3.0
0 1 2 3 4 5 6 7
92
0 2 4 6 8
0 1 2 3 4 5 6
93
0.0 0.1 0.2 0.3 0.4 0.5
0 5 10 15
94
0.0 0.1 0.2 0.3 0.4 0.5 0.6 0.7
0.0 0.5 1.0 1.5 2.0 2.5 3.0 3.5
95
0.0 0.2 0.4 0.6 0.8 1.0 1.2 1.4
0 1 2 3 4 5
96
0.0 0.1 0.2 0.3 0.4 0.5 0.6 0.7
0 1 2 3 4 5 6
97
0.0 0.2 0.4 0.6 0.8
0 1 2 3 4
98
0.0 0.2 0.4 0.6 0.8 1.0 1.2
0.0 0.5 1.0 1.5 2.0 2.5 3.0
99
0.0 0.2 0.4 0.6 0.8
0 1 2 3 4
100
0.0 0.1 0.2 0.3 0.4 0.5
0.0 0.5 1.0 1.5 2.0
101
0.0 0.1 0.2 0.3 0.4
0 1 2 3 4
102
0.0 0.2 0.4 0.6 0.8
0.0 0.5 1.0 1.5 2.0 2.5 3.0
103
0.0 0.1 0.2 0.3 0.4 0.5
0 1 2 3 4
104
0.0 0.5 1.0 1.5 2.0
0 1 2 3 4 5
105

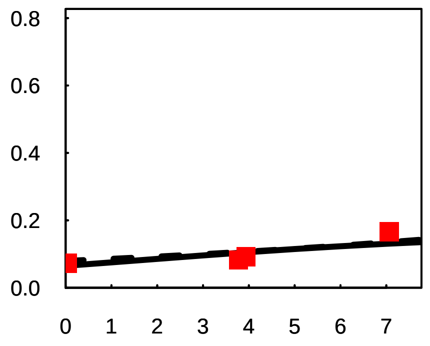

106

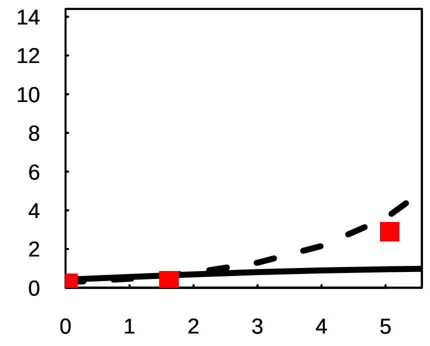

107

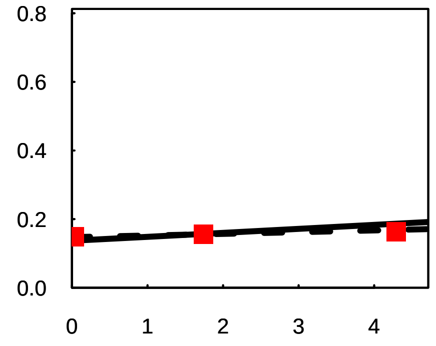

108

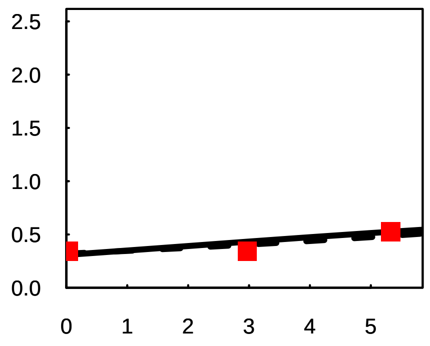

109

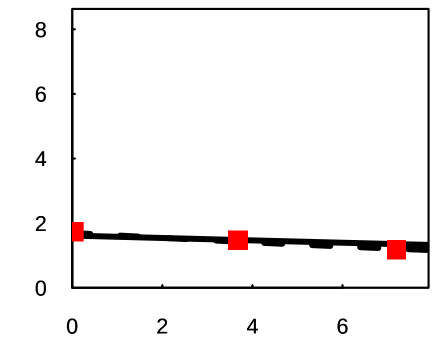

110

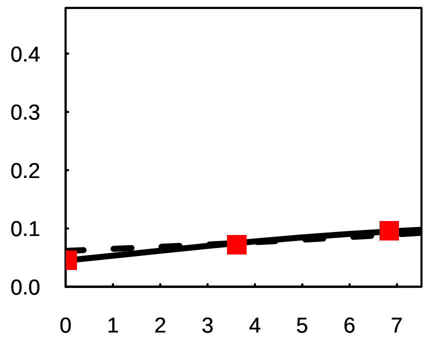

111

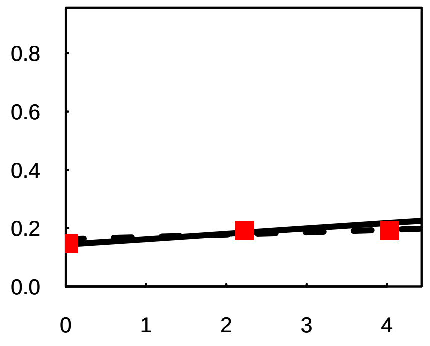

112

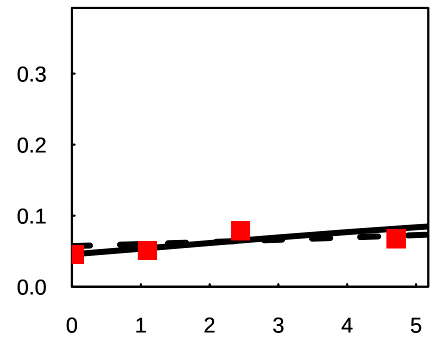

113

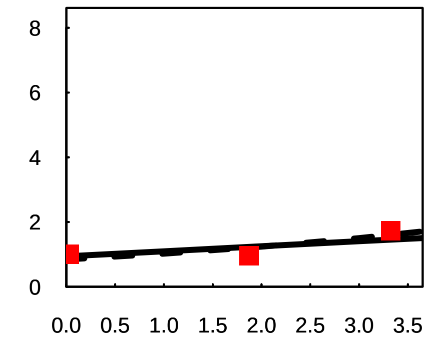

114

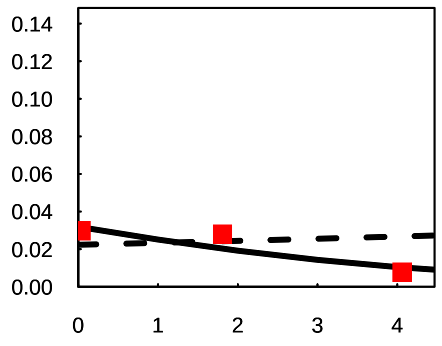

115

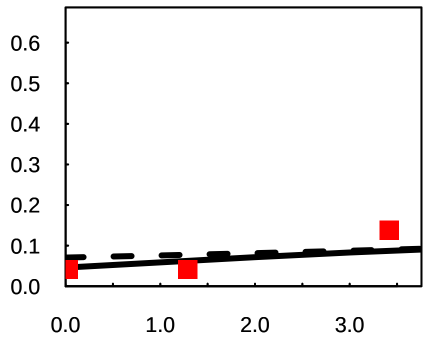

116

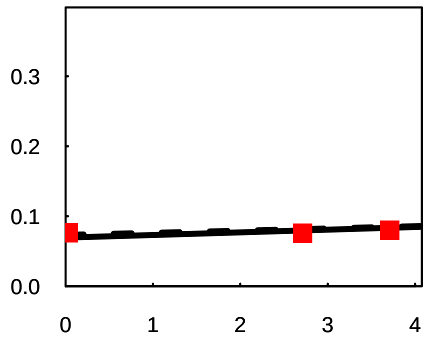

117

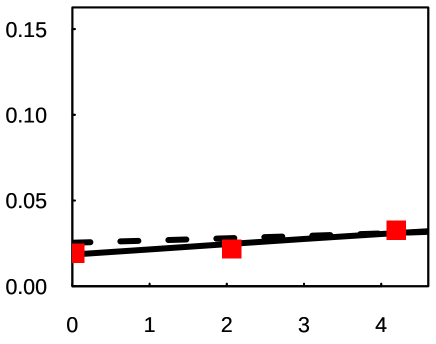

118

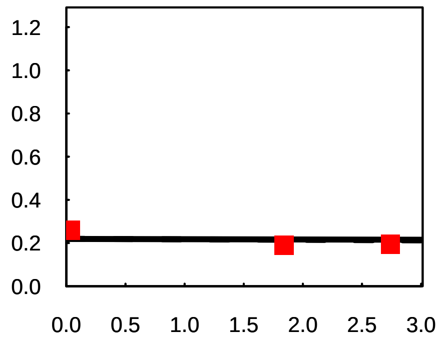

119

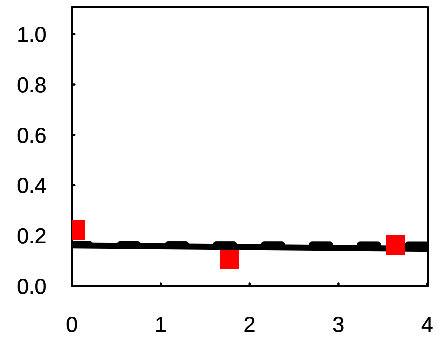

120

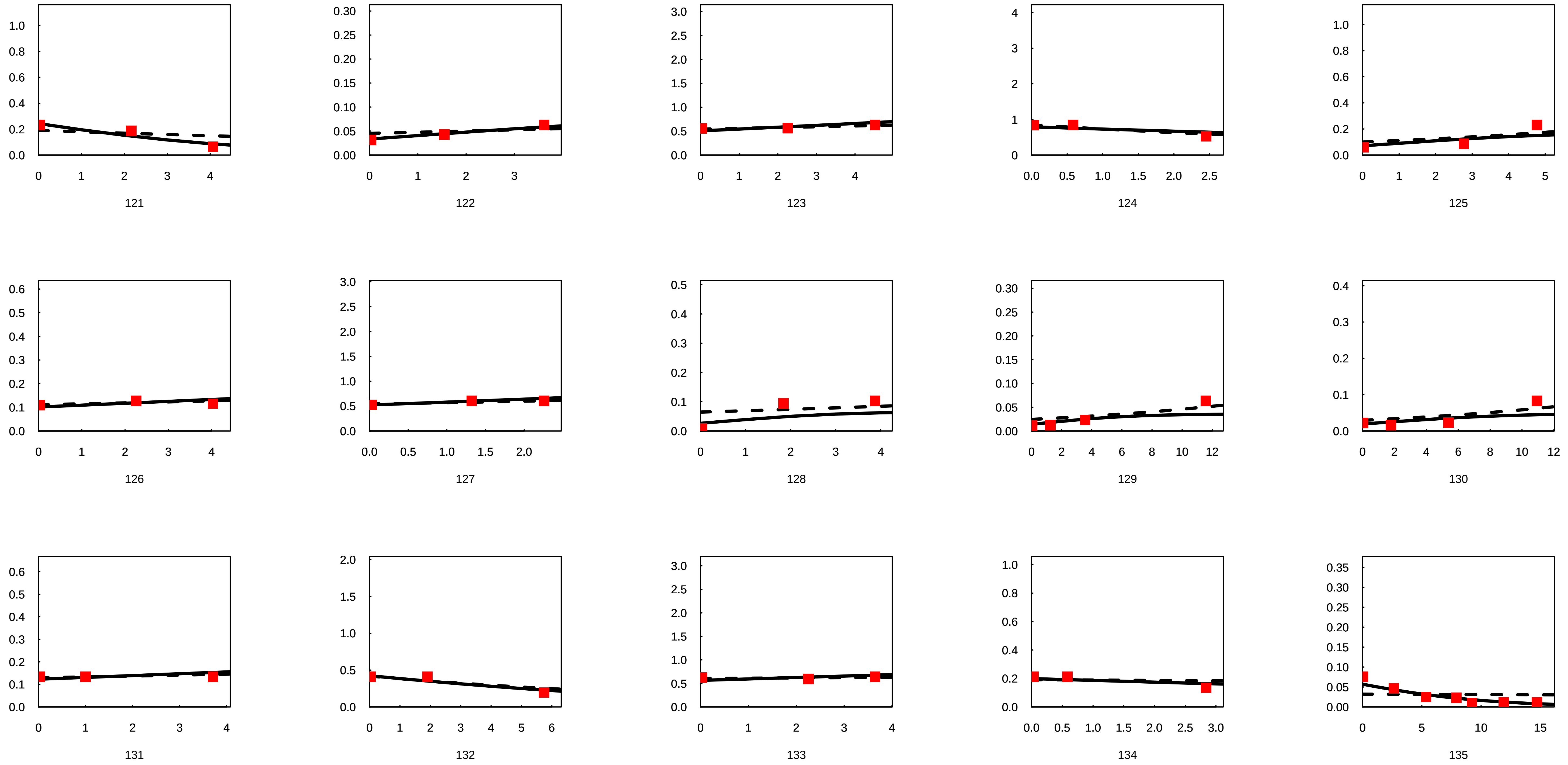
121
122
123
124
125
126
127
128
129
130
131
132
133
134
135

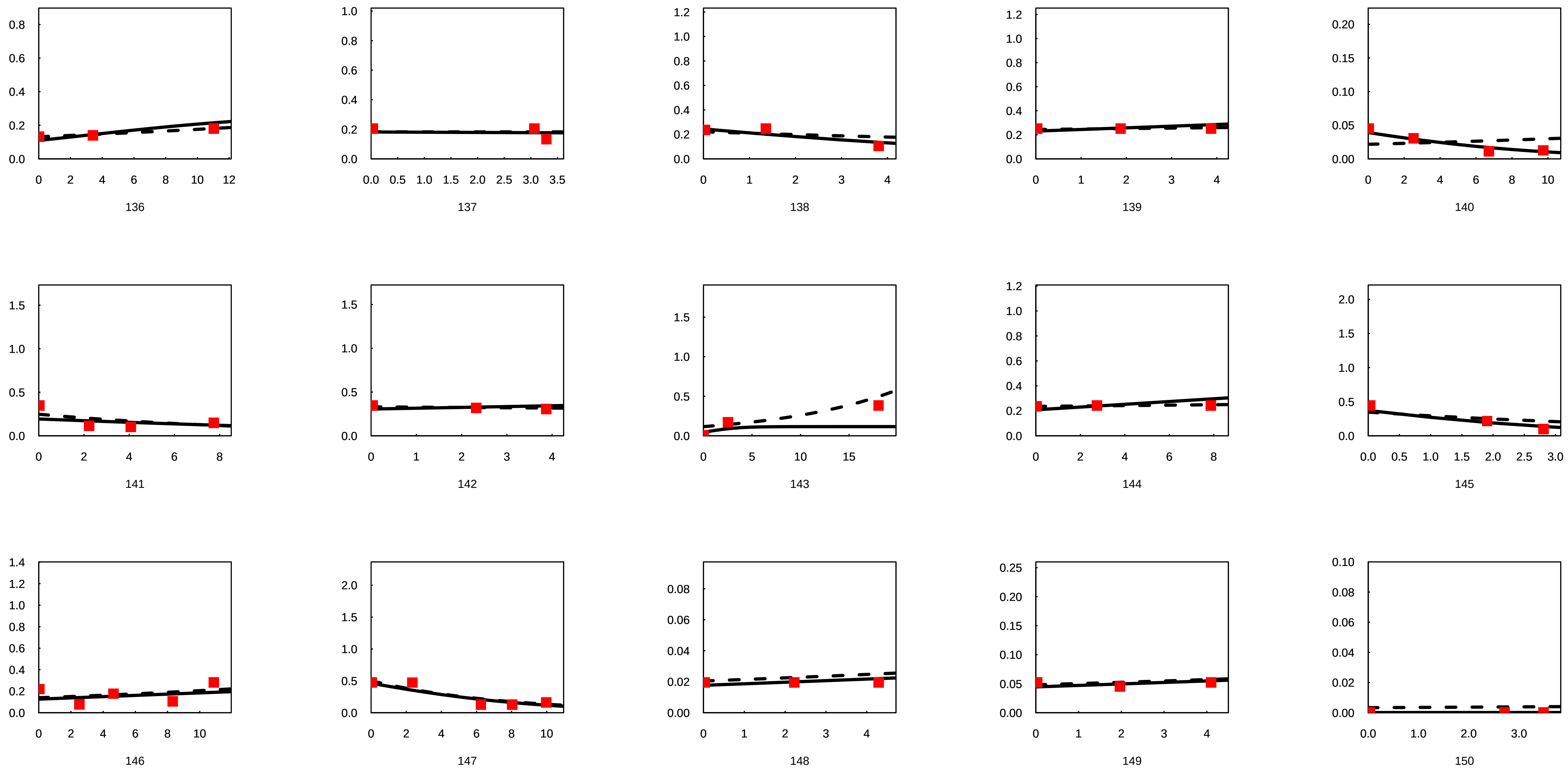
136
137
138
139
140
141
142
143
144
145
146
147
148
149
150

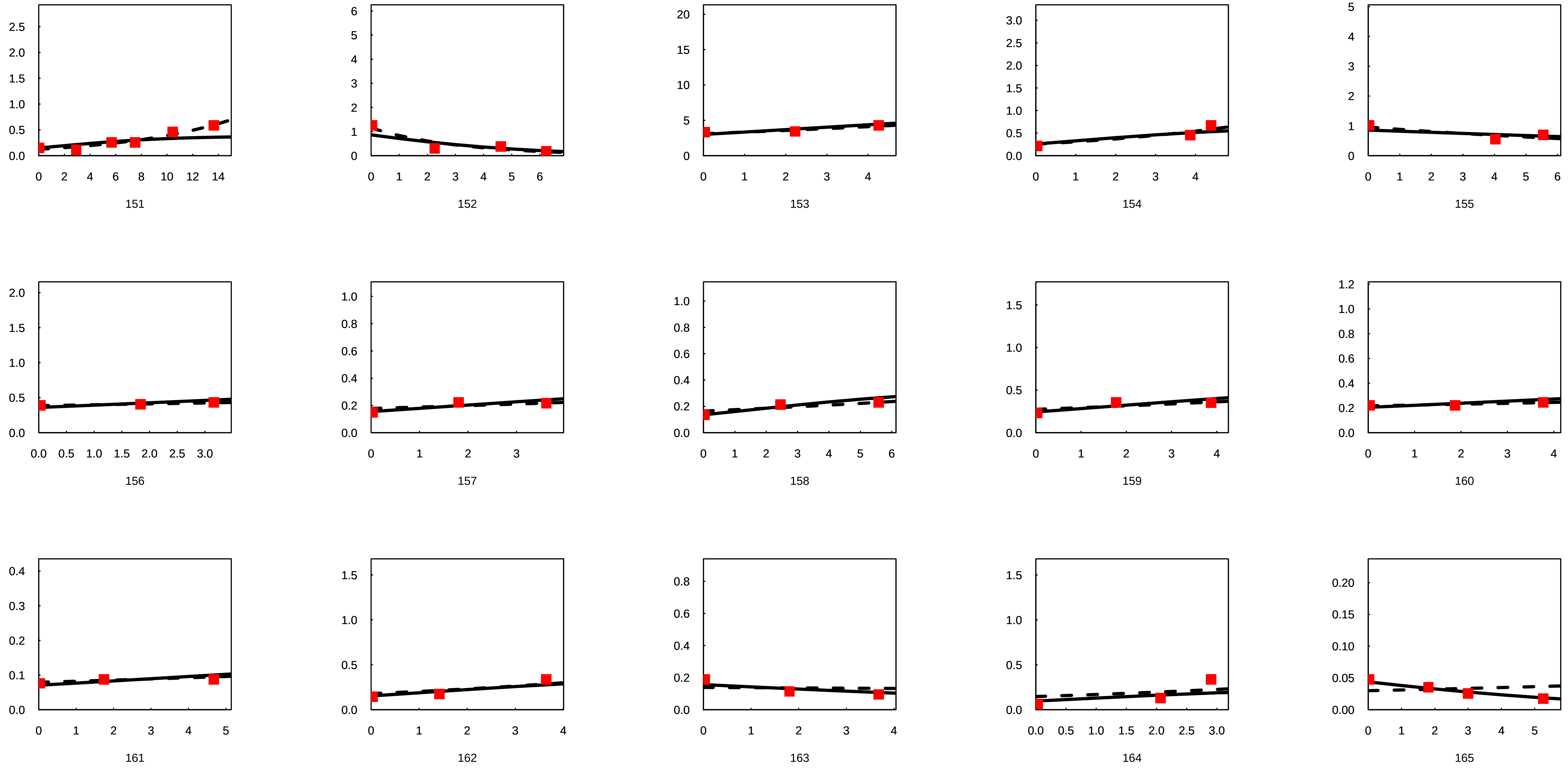
0.0 0.5 1.0 1.5 2.0 2.5
0 2 4 6 8 10 12 14
151
0 1 2 3 4 5 6
0 1 2 3 4 5 6
152
0 5 10 15 20
0 1 2 3 4
153
0.0 0.5 1.0 1.5 2.0 2.5 3.0
0 1 2 3 4
154
0 1 2 3 4 5
0 1 2 3 4 5 6
155
0.0 0.5 1.0 1.5 2.0
0.0 0.5 1.0 1.5 2.0 2.5 3.0
156
0.0 0.2 0.4 0.6 0.8 1.0
0 1 2 3
157
0.0 0.2 0.4 0.6 0.8 1.0
0 1 2 3 4 5 6
158
0.0 0.5 1.0 1.5
0 1 2 3 4
159
0.0 0.2 0.4 0.6 0.8 1.0 1.2
0 1 2 3 4
160
0.0 0.1 0.2 0.3 0.4
0 1 2 3 4 5
161
0.0 0.5 1.0 1.5
0 1 2 3 4
162
0.0 0.2 0.4 0.6 0.8
0 1 2 3 4
163
0.0 0.5 1.0 1.5
0.0 0.5 1.0 1.5 2.0 2.5 3.0
164
0.00 0.05 0.10 0.15 0.20
0 1 2 3 4 5
165

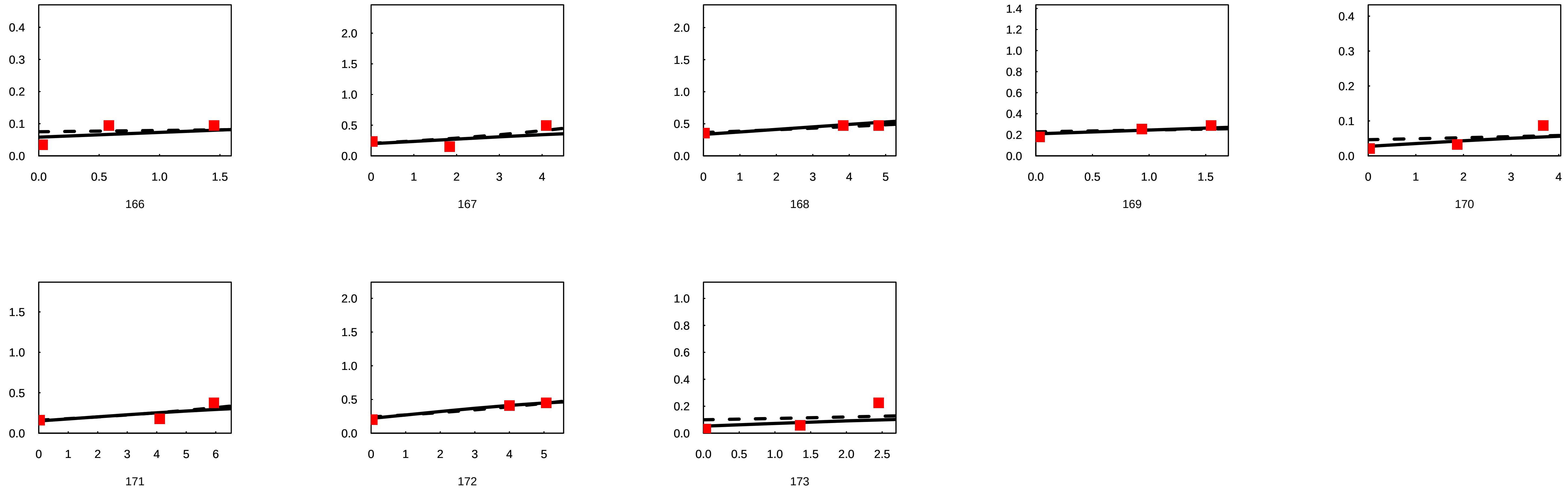
166
167
168
169
170
171
172
173

Metastasis gemcitabine

Y axis: Tumor volume/10^2 (cm^3)

X axis: Months

line: logistic model

dotted line: exponential model

red square: data

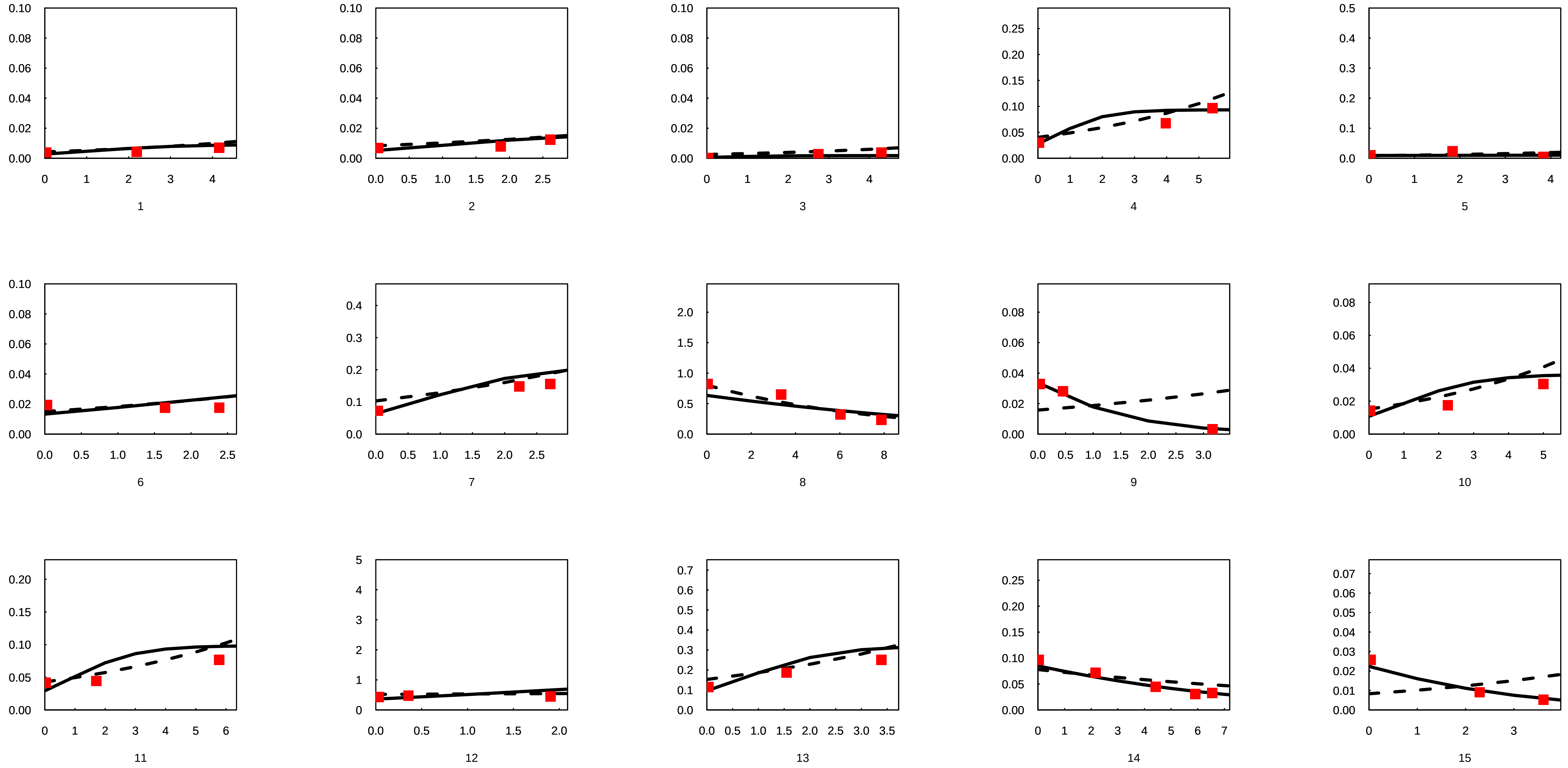
0.10 0.08 0.06 0.04 0.02 0.00
0 1 2 3 4
1
0.10 0.08 0.06 0.04 0.02 0.00
0.0 0.5 1.0 1.5 2.0 2.5
2
0.10 0.08 0.06 0.04 0.02 0.00
0 1 2 3 4
3
0.25 0.20 0.15 0.10 0.05 0.00
0 1 2 3 4 5
4
0.5 0.4 0.3 0.2 0.1 0.0
0 1 2 3 4
5
0.10 0.08 0.06 0.04 0.02 0.00
0.0 0.5 1.0 1.5 2.0 2.5
6
0.4 0.3 0.2 0.1 0.0
0.0 0.5 1.0 1.5 2.0 2.5
7
2.0 1.5 1.0 0.5 0.0
0 2 4 6 8
8
0.08 0.06 0.04 0.02 0.00
0.0 0.5 1.0 1.5 2.0 2.5 3.0
9
0.08 0.06 0.04 0.02 0.00
0 1 2 3 4 5
10
0.20 0.15 0.10 0.05 0.00
0 1 2 3 4 5 6
11
5 4 3 2 1 0
0.0 0.5 1.0 1.5 2.0
12
0.7 0.6 0.5 0.4 0.3 0.2 0.1 0.0
0.0 0.5 1.0 1.5 2.0 2.5 3.0 3.5
13
0.25 0.20 0.15 0.10 0.05 0.00
0 1 2 3 4 5 6 7
14
0.07 0.06 0.05 0.04 0.03 0.02 0.01 0.00
0 1 2 3
15

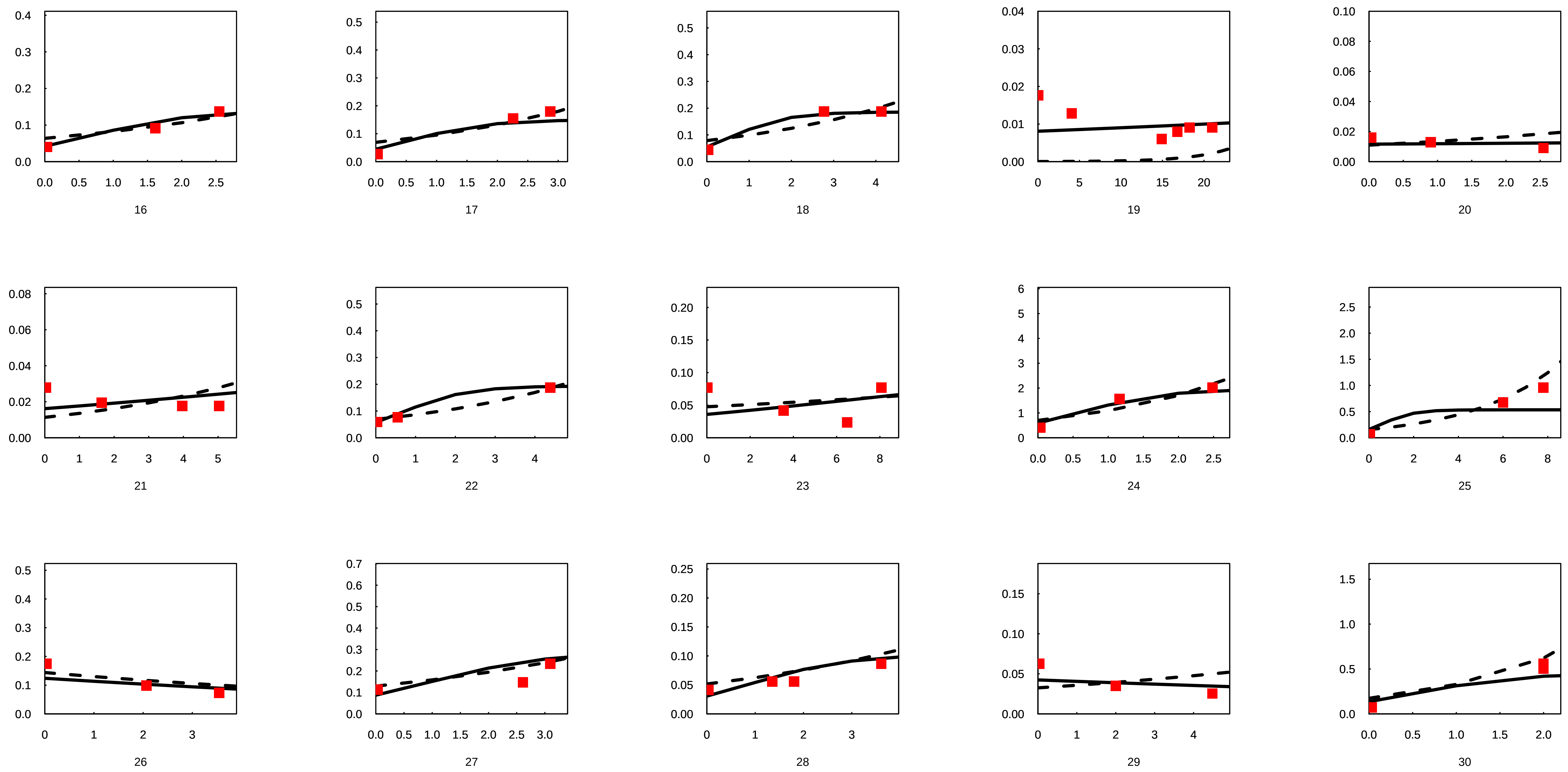
16
17
18
19
20
21
22
23
24
25
26
27
28
29
30

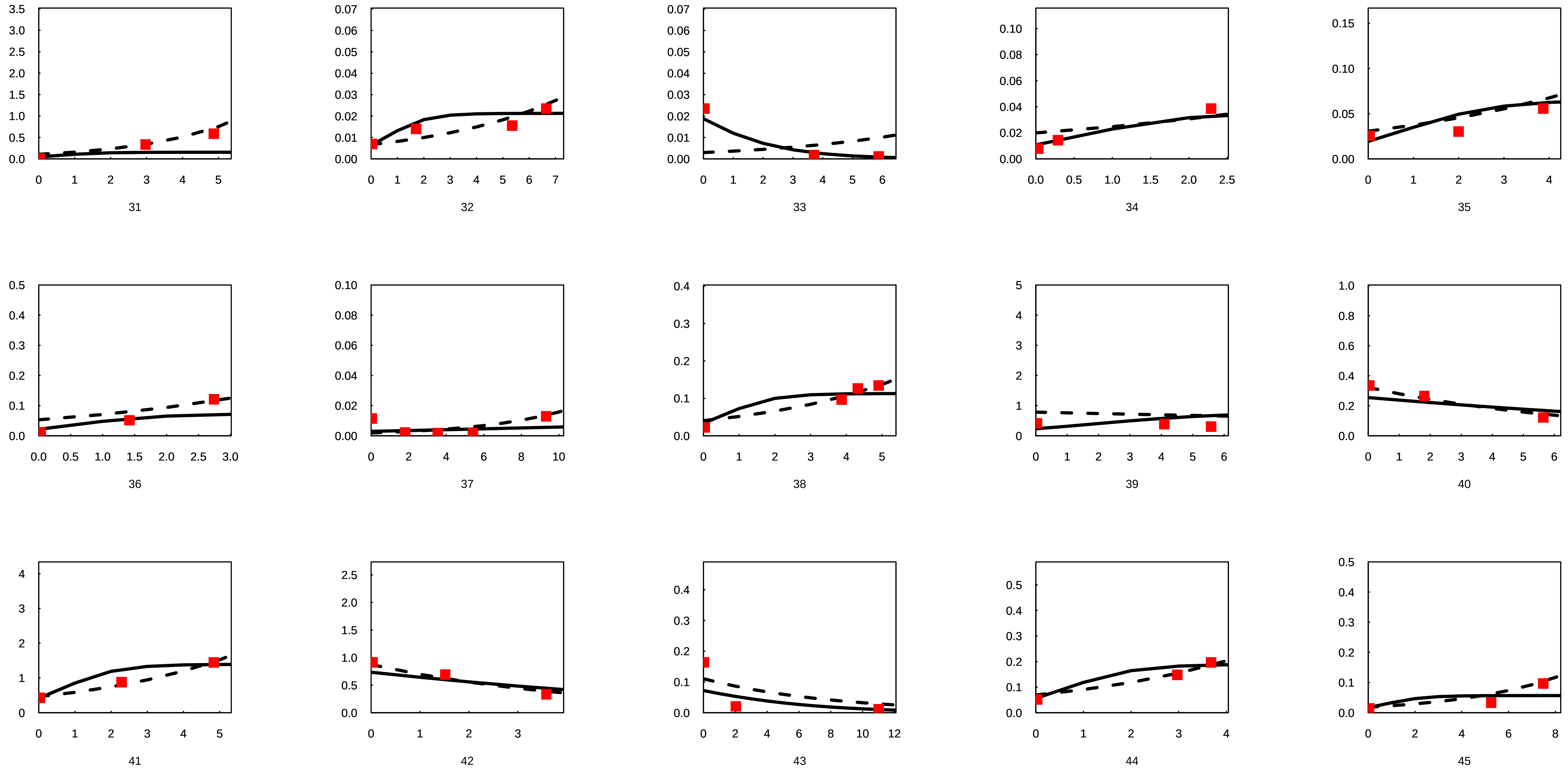

3.5 3.0 2.5 2.0 1.5 1.0 0.5 0.0
0 1 2 3 4 5
31
0.07 0.06 0.05 0.04 0.03 0.02 0.01 0.00
0 1 2 3 4 5 6 7
32
0.07 0.06 0.05 0.04 0.03 0.02 0.01 0.00
0 1 2 3 4 5 6
33
0.10 0.08 0.06 0.04 0.02 0.00
0.0 0.5 1.0 1.5 2.0 2.5
34
0.15 0.10 0.05 0.00
0 1 2 3 4
35
0.5 0.4 0.3 0.2 0.1 0.0
0.0 0.5 1.0 1.5 2.0 2.5 3.0
36
0.10 0.08 0.06 0.04 0.02 0.00
0 2 4 6 8 10
37
0.4 0.3 0.2 0.1 0.0
0 1 2 3 4 5
38
5 4 3 2 1 0
0 1 2 3 4 5 6
39
1.0 0.8 0.6 0.4 0.2 0.0
0 1 2 3 4 5 6
40
4 3 2 1 0
0 1 2 3 4 5
41
2.5 2.0 1.5 1.0 0.5 0.0
0 1 2 3
42
0.4 0.3 0.2 0.1 0.0
0 2 4 6 8 10 12
43
0.5 0.4 0.3 0.2 0.1 0.0
0 1 2 3 4
44
0.5 0.4 0.3 0.2 0.1 0.0
0 2 4 6 8
45

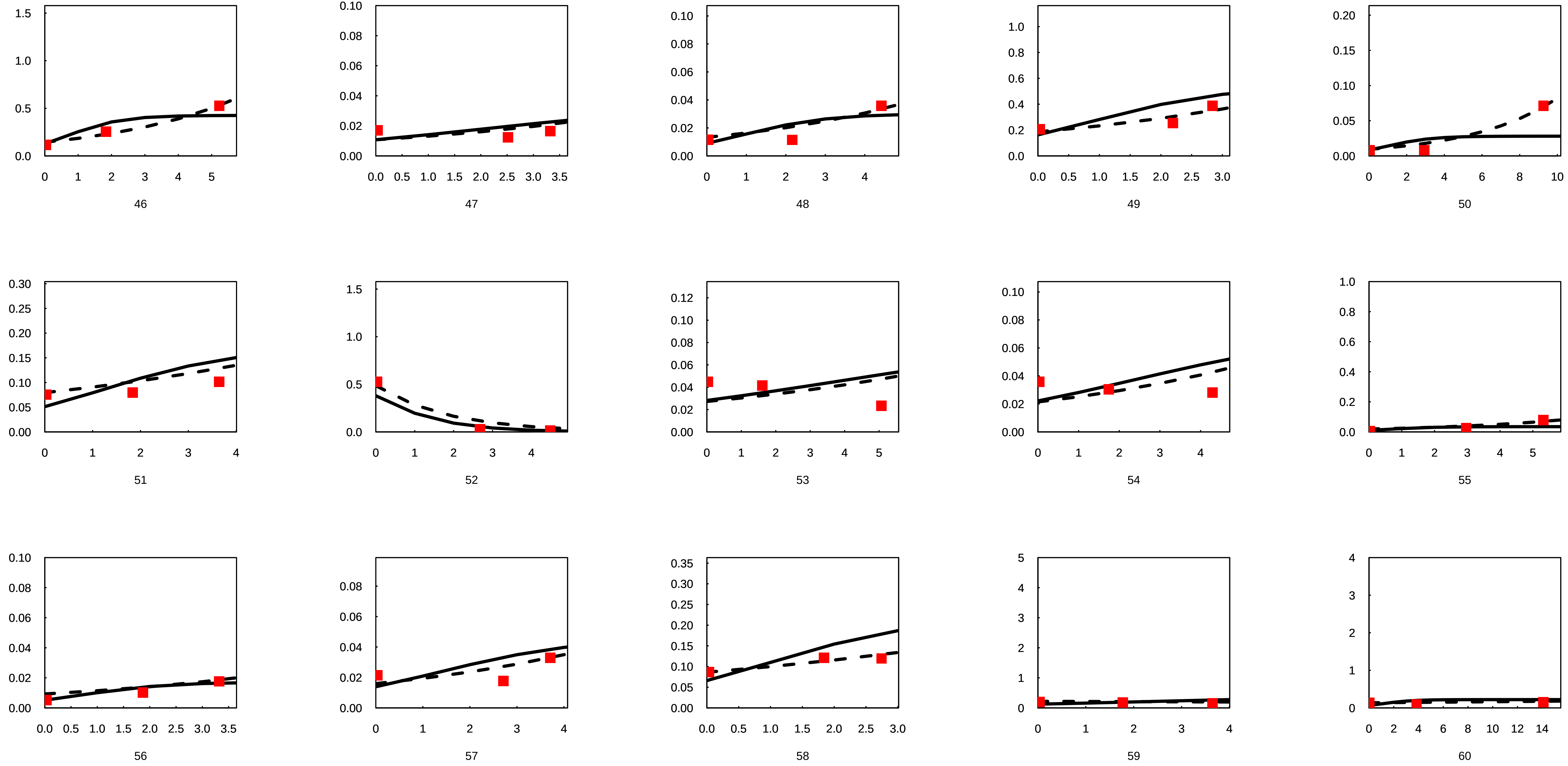

1.5 1.0 0.5 0.0
0 1 2 3 4 5
46
0.10 0.08 0.06 0.04 0.02 0.00
0.0 0.5 1.0 1.5 2.0 2.5 3.0 3.5
47
0.10 0.08 0.06 0.04 0.02 0.00
0 1 2 3 4
48
1.0 0.8 0.6 0.4 0.2 0.0
0.0 0.5 1.0 1.5 2.0 2.5 3.0
49
0.20 0.15 0.10 0.05 0.00
0 2 4 6 8 10
50
0.30 0.25 0.20 0.15 0.10 0.05 0.00
0 1 2 3 4
51
1.5 1.0 0.5 0.0
0 1 2 3 4
52
0.12 0.10 0.08 0.06 0.04 0.02 0.00
0 1 2 3 4 5
53
0.10 0.08 0.06 0.04 0.02 0.00
0 1 2 3 4
54
1.0 0.8 0.6 0.4 0.2 0.0
0 1 2 3 4 5
55
0.10 0.08 0.06 0.04 0.02 0.00
0.0 0.5 1.0 1.5 2.0 2.5 3.0 3.5
56
0.08 0.06 0.04 0.02 0.00
0 1 2 3 4
57
0.35 0.30 0.25 0.20 0.15 0.10 0.05 0.00
0.0 0.5 1.0 1.5 2.0 2.5 3.0
58
5 4 3 2 1 0
0 1 2 3 4
59
4 3 2 1 0
0 2 4 6 8 10 12 14
60

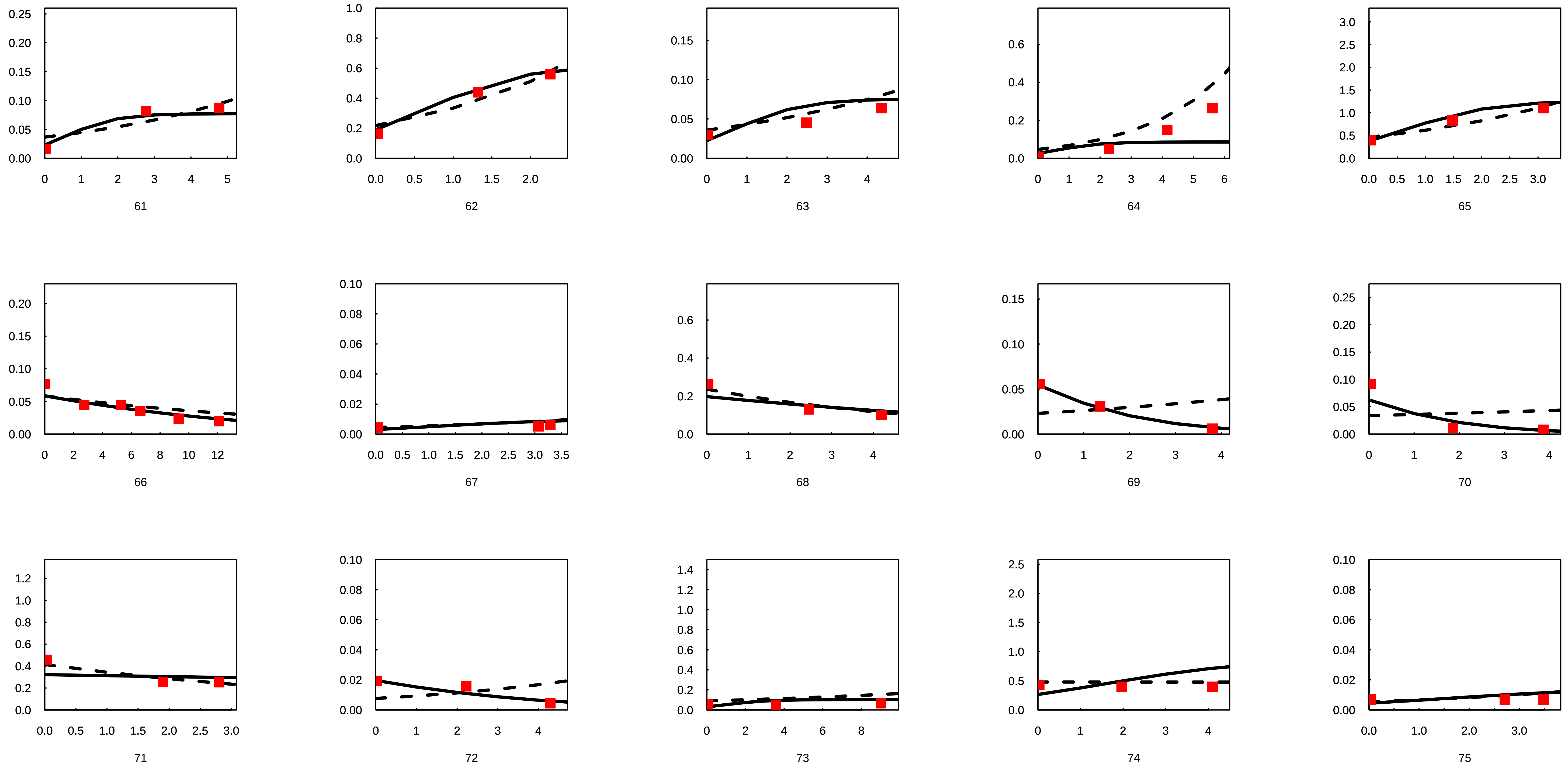
61
62
63
64
65
66
67
68
69
70
71
72
73
74
75

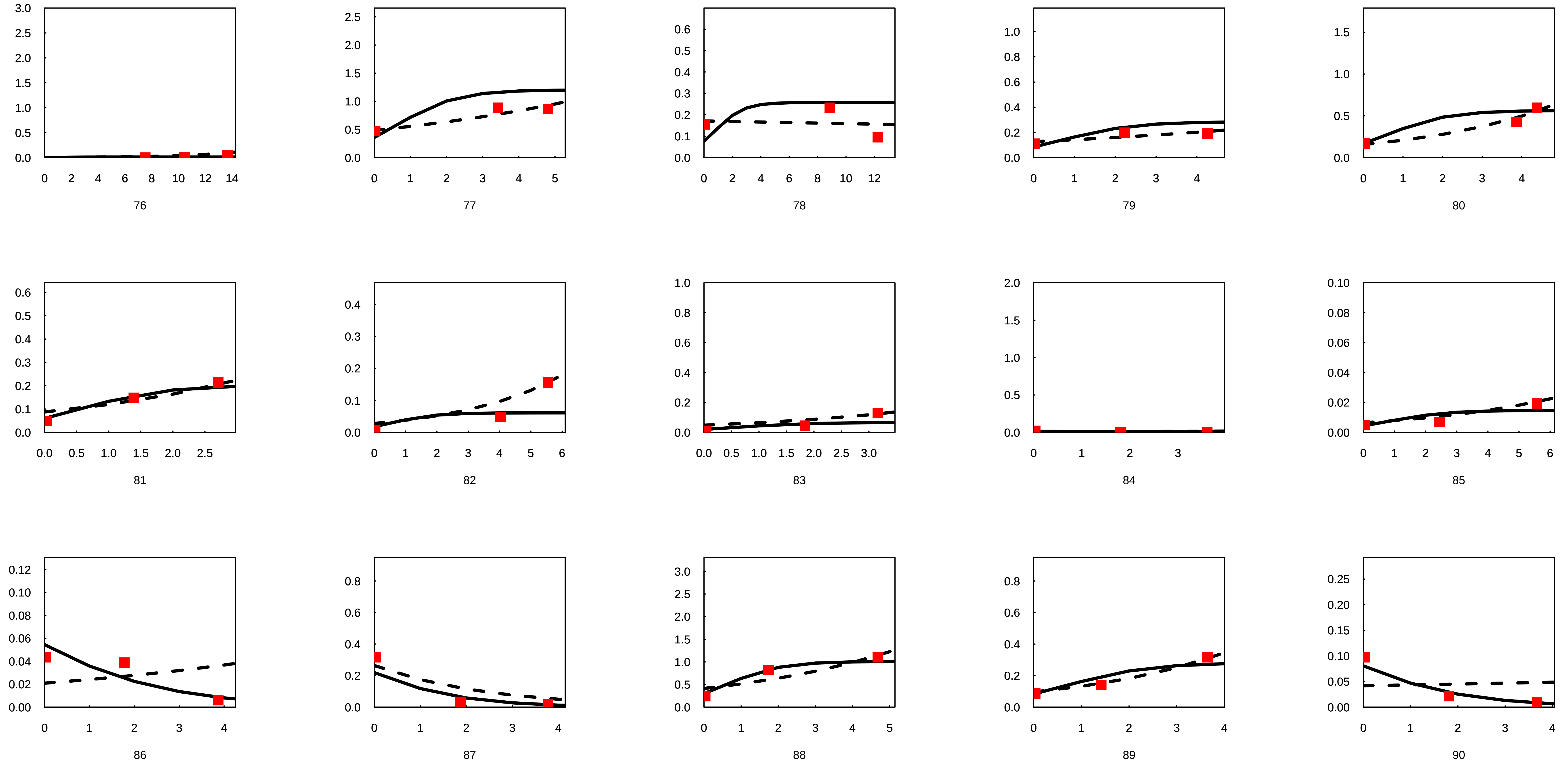
0.0 0.5 1.0 1.5 2.0 2.5 3.0
0 2 4 6 8 10 12 14
76
0.0 0.5 1.0 1.5 2.0 2.5
0 1 2 3 4 5
77
0.0 0.1 0.2 0.3 0.4 0.5 0.6
0 2 4 6 8 10 12
78
0.0 0.2 0.4 0.6 0.8 1.0
0 1 2 3 4
79
0.0 0.5 1.0 1.5
0 1 2 3 4
80
0.0 0.1 0.2 0.3 0.4 0.5 0.6
0.0 0.5 1.0 1.5 2.0 2.5
81
0.0 0.1 0.2 0.3 0.4
0 1 2 3 4 5 6
82
0.0 0.2 0.4 0.6 0.8 1.0
0.0 0.5 1.0 1.5 2.0 2.5 3.0
83
0.0 0.5 1.0 1.5 2.0
0 1 2 3
84
0.00 0.02 0.04 0.06 0.08 0.10
0 1 2 3 4 5 6
85
0.00 0.02 0.04 0.06 0.08 0.10 0.12
0 1 2 3 4
86
0.0 0.2 0.4 0.6 0.8
0 1 2 3 4
87
0.0 0.5 1.0 1.5 2.0 2.5 3.0
0 1 2 3 4 5
88
0.0 0.2 0.4 0.6 0.8
0 1 2 3 4
89
0.00 0.05 0.10 0.15 0.20 0.25
0 1 2 3 4
90

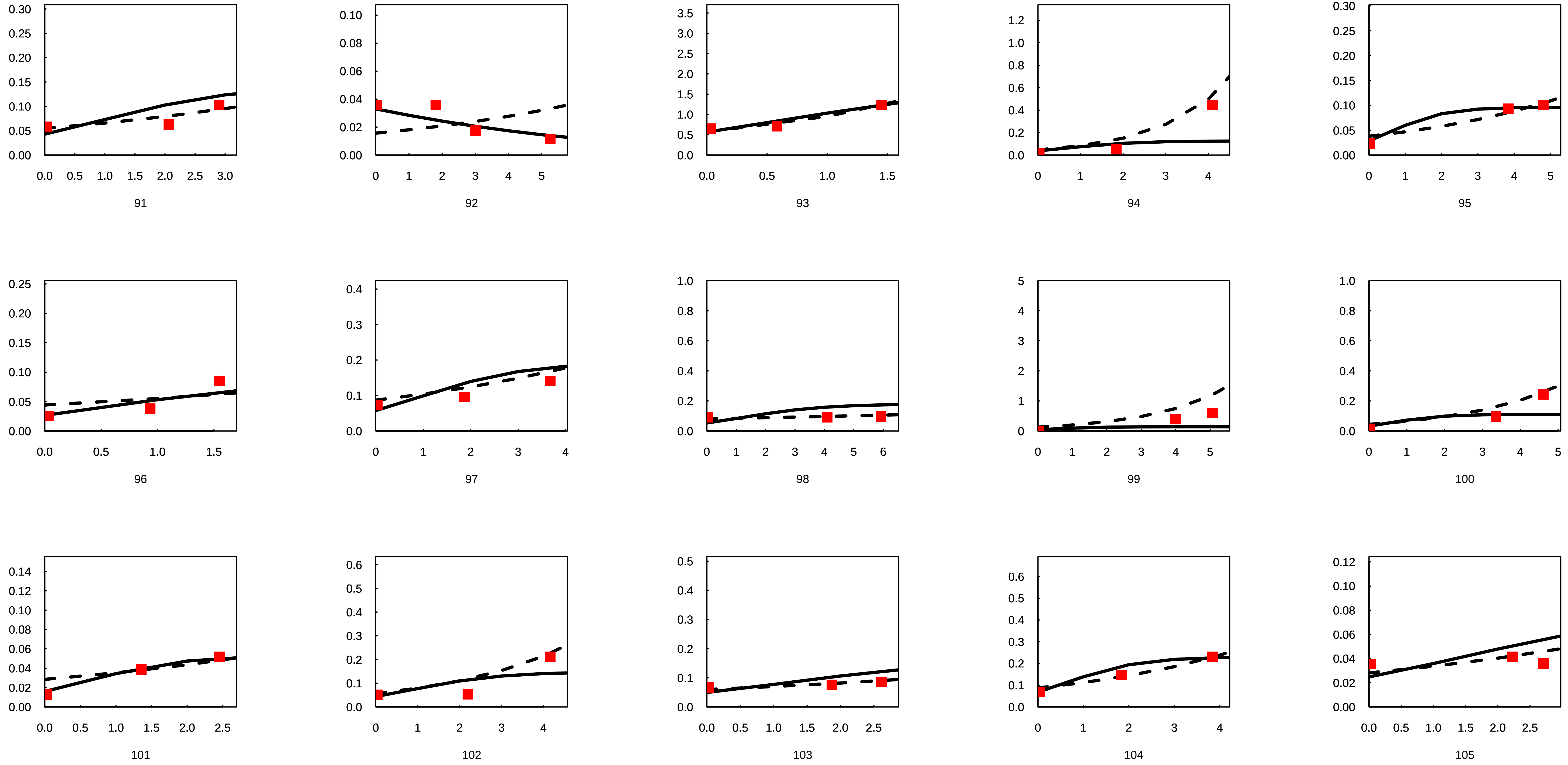
91
92
93
94
95
96
97
98
99
100
101
102
103
104
105

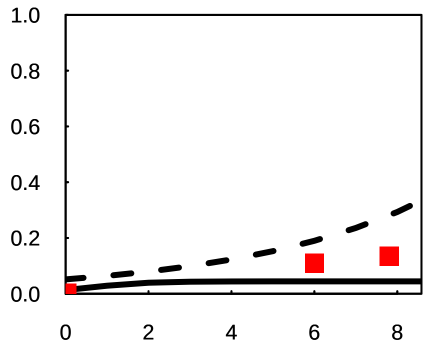
1.0
0.8
0.6
0.4
0.2
0.0
0
2
4
6
8

106

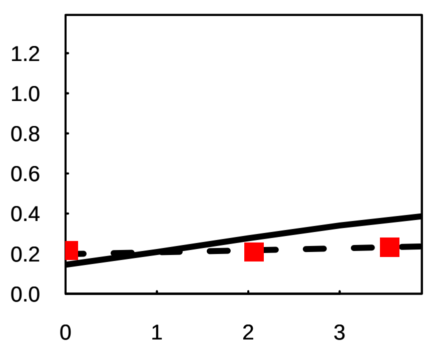
1.2
1.0
0.8
0.6
0.4
0.2
0.0
0
1
2
3

107

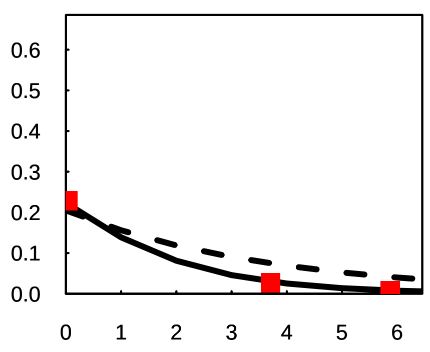
0.6
0.5
0.4
0.3
0.2
0.1
0.0
0
1
2
3
4
5
6

108

Primary FOLFIRINOX

Y axis: Tumor volume/10^2 (cm^3)

X axis: Months

line: logistic model

dotted line: exponential model

red square: data

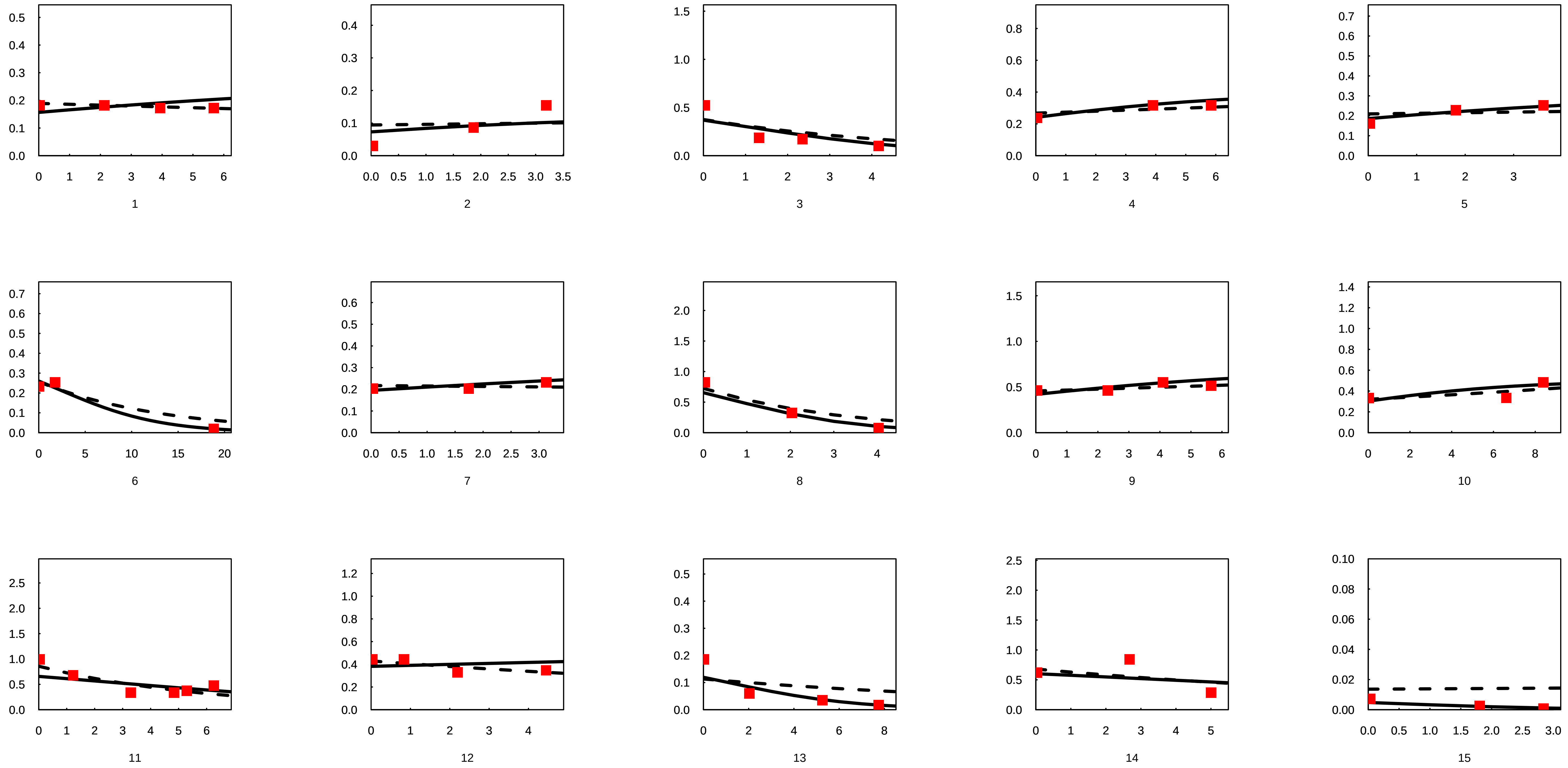
1
2
3
4
5
6
7
8
9
10
11
12
13
14
15

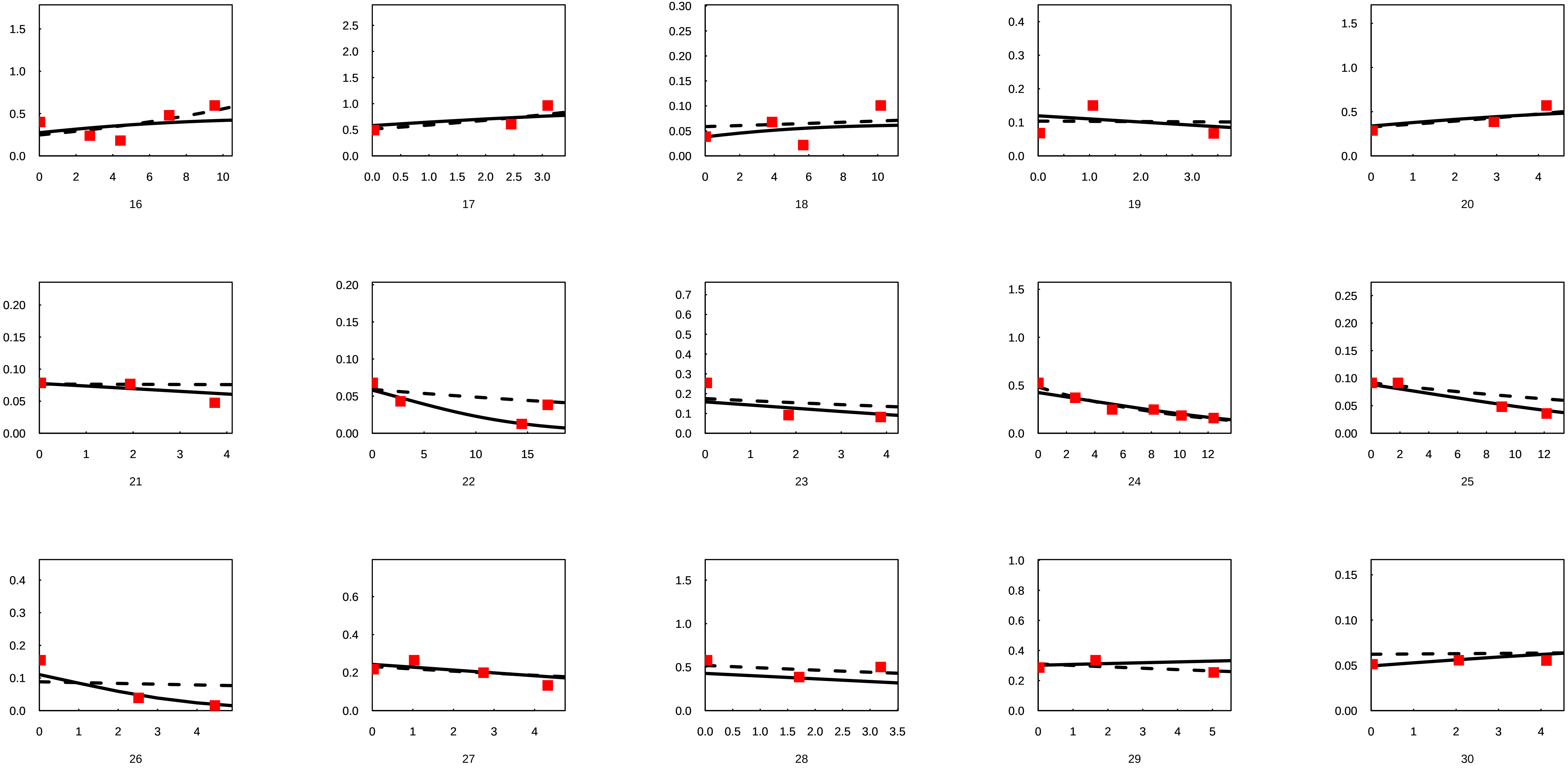

0.0 0.5 1.0 1.5
0 2 4 6 8 10
16
0.0 0.5 1.0 1.5 2.0 2.5
0.0 0.5 1.0 1.5 2.0 2.5 3.0
17
0.00 0.05 0.10 0.15 0.20 0.25 0.30
0 2 4 6 8 10
18
0.0 0.1 0.2 0.3 0.4
0.0 1.0 2.0 3.0
19
0.0 0.5 1.0 1.5
0 1 2 3 4
20
0.00 0.05 0.10 0.15 0.20
0 1 2 3 4
21
0.00 0.05 0.10 0.15 0.20
0 5 10 15
22
0.0 0.1 0.2 0.3 0.4 0.5 0.6 0.7
0 1 2 3 4
23
0.0 0.5 1.0 1.5
0 2 4 6 8 10 12
24
0.00 0.05 0.10 0.15 0.20 0.25
0 2 4 6 8 10 12
25
0.0 0.1 0.2 0.3 0.4
0 1 2 3 4
26
0.0 0.2 0.4 0.6
0 1 2 3 4
27
0.0 0.5 1.0 1.5
0.0 0.5 1.0 1.5 2.0 2.5 3.0 3.5
28
0.0 0.2 0.4 0.6 0.8 1.0
0 1 2 3 4 5
29
0.00 0.05 0.10 0.15
0 1 2 3 4
30

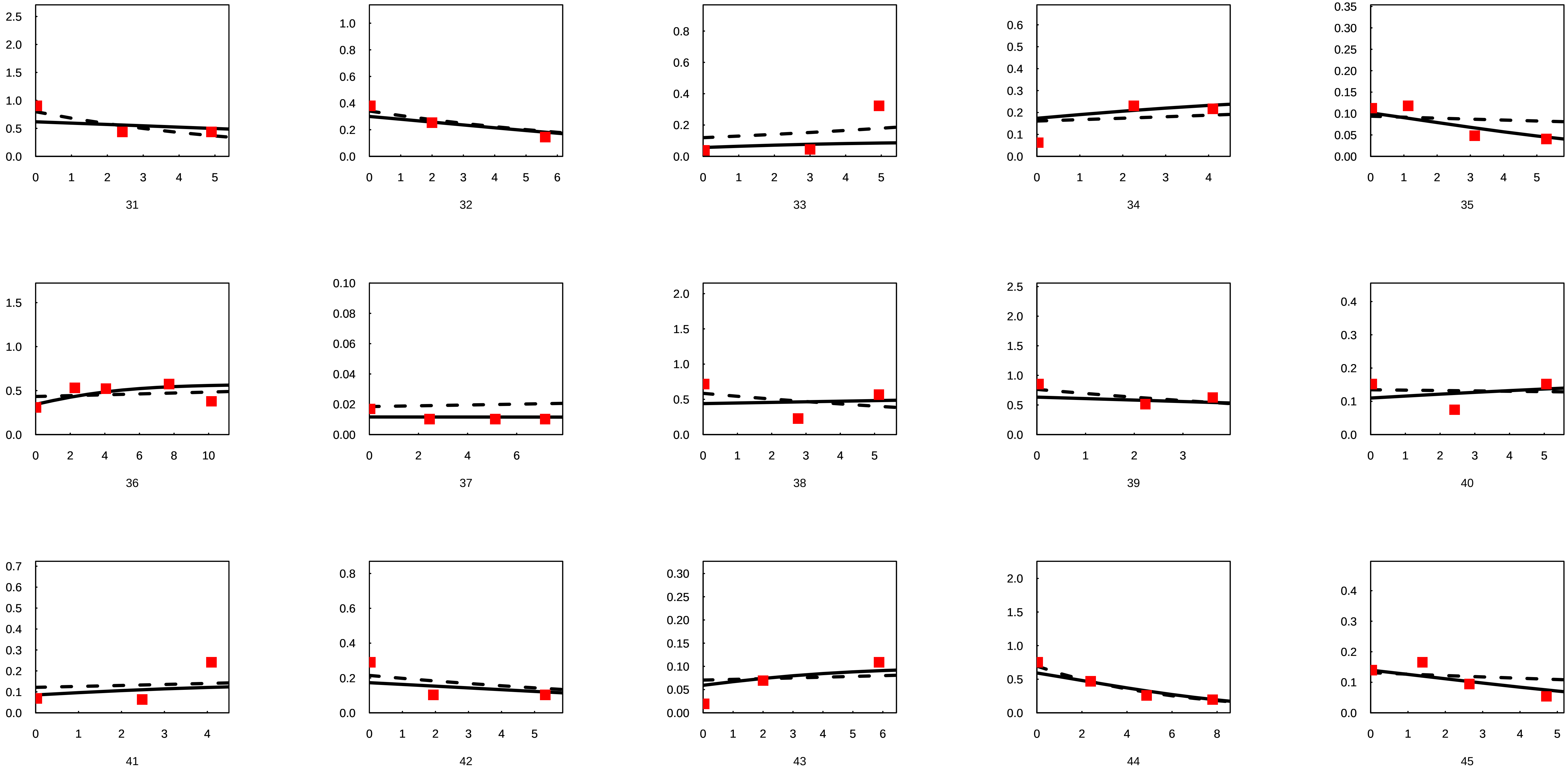
31
32
33
34
35
36
37
38
39
40
41
42
43
44
45

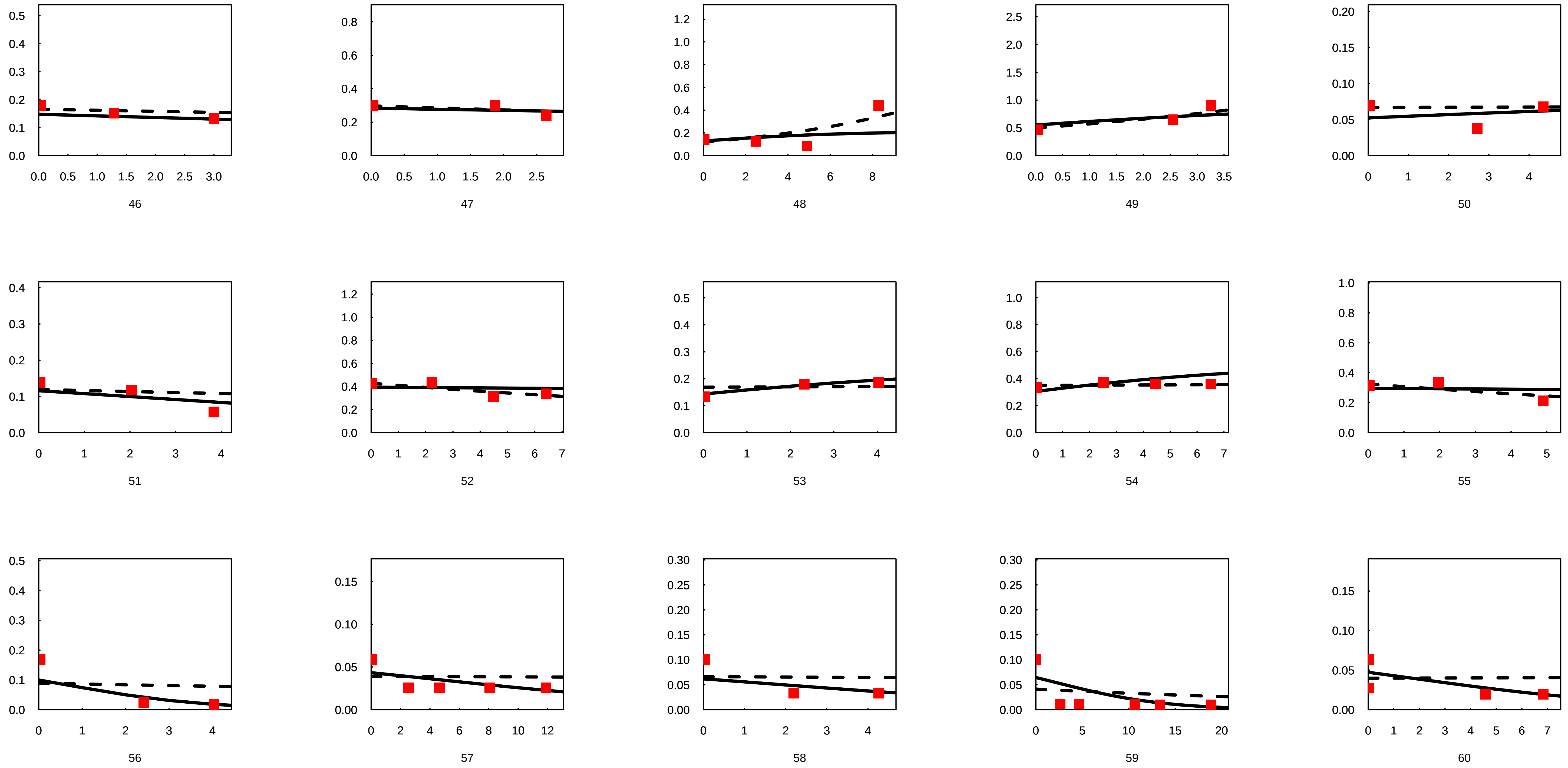
46
47
48
49
50
51
52
53
54
55
56
57
58
59
60

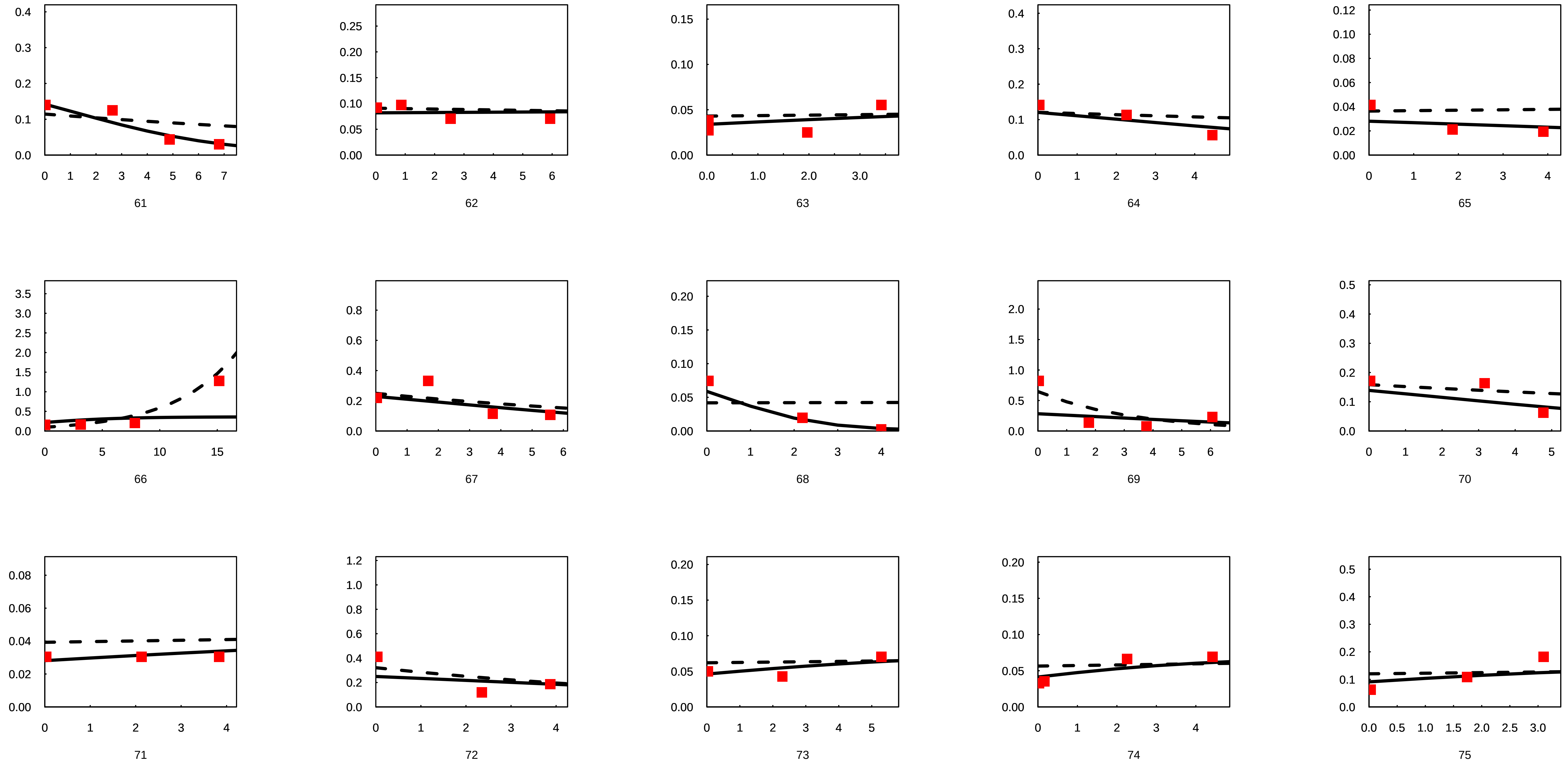

0.4 0.3 0.2 0.1 0.0
0 1 2 3 4 5 6 7
61
0.25 0.20 0.15 0.10 0.05 0.00
0 1 2 3 4 5 6
62
0.15 0.10 0.05 0.00
0.0 1.0 2.0 3.0
63
0.4 0.3 0.2 0.1 0.0
0 1 2 3 4
64
0.12 0.10 0.08 0.06 0.04 0.02 0.00
0 1 2 3 4
65
3.5 3.0 2.5 2.0 1.5 1.0 0.5 0.0
0 5 10 15
66
0.8 0.6 0.4 0.2 0.0
0 1 2 3 4 5 6
67
0.20 0.15 0.10 0.05 0.00
0 1 2 3 4
68
2.0 1.5 1.0 0.5 0.0
0 1 2 3 4 5 6
69
0.5 0.4 0.3 0.2 0.1 0.0
0 1 2 3 4 5
70
0.08 0.06 0.04 0.02 0.00
0 1 2 3 4
71
1.2 1.0 0.8 0.6 0.4 0.2 0.0
0 1 2 3 4
72
0.20 0.15 0.10 0.05 0.00
0 1 2 3 4 5
73
0.20 0.15 0.10 0.05 0.00
0 1 2 3 4
74
0.5 0.4 0.3 0.2 0.1 0.0
0.0 0.5 1.0 1.5 2.0 2.5 3.0
75

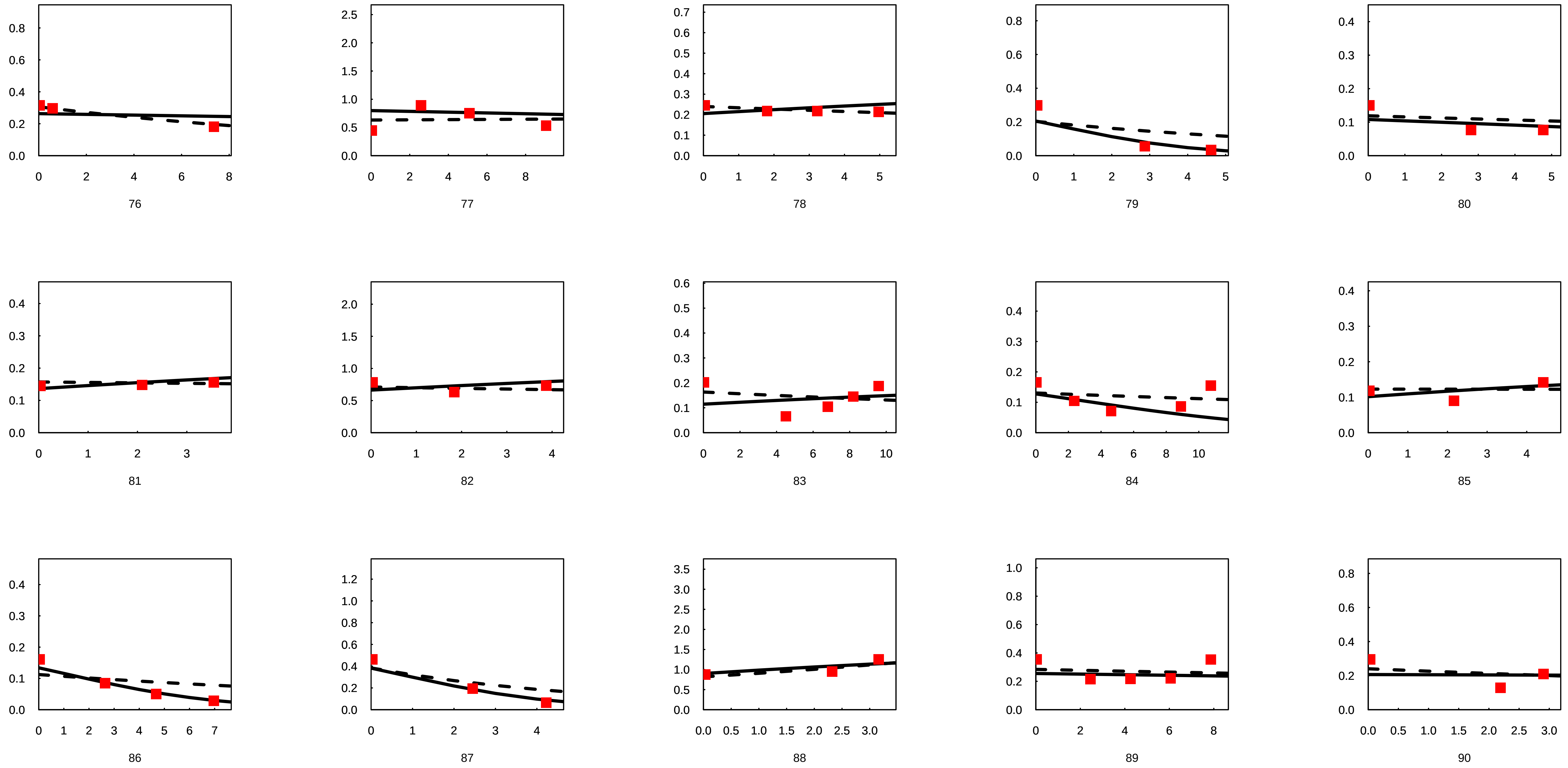
0.0 0.2 0.4 0.6 0.8
0 2 4 6 8
76
0.0 0.5 1.0 1.5 2.0 2.5
0 2 4 6 8
77
0.0 0.1 0.2 0.3 0.4 0.5 0.6 0.7
0 1 2 3 4 5
78
0.0 0.2 0.4 0.6 0.8
0 1 2 3 4 5
79
0.0 0.1 0.2 0.3 0.4
0 1 2 3 4 5
80
0.0 0.1 0.2 0.3 0.4
0 1 2 3
81
0.0 0.5 1.0 1.5 2.0
0 1 2 3 4
82
0.0 0.1 0.2 0.3 0.4 0.5 0.6
0 2 4 6 8 10
83
0.0 0.1 0.2 0.3 0.4
0 2 4 6 8 10
84
0.0 0.1 0.2 0.3 0.4
0 1 2 3 4
85
0.0 0.1 0.2 0.3 0.4
0 1 2 3 4 5 6 7
86
0.0 0.2 0.4 0.6 0.8 1.0 1.2
0 1 2 3 4
87
0.0 0.5 1.0 1.5 2.0 2.5 3.0 3.5
0.0 0.5 1.0 1.5 2.0 2.5 3.0
88
0.0 0.2 0.4 0.6 0.8 1.0
0 2 4 6 8
89
0.0 0.2 0.4 0.6 0.8
0.0 0.5 1.0 1.5 2.0 2.5 3.0
90

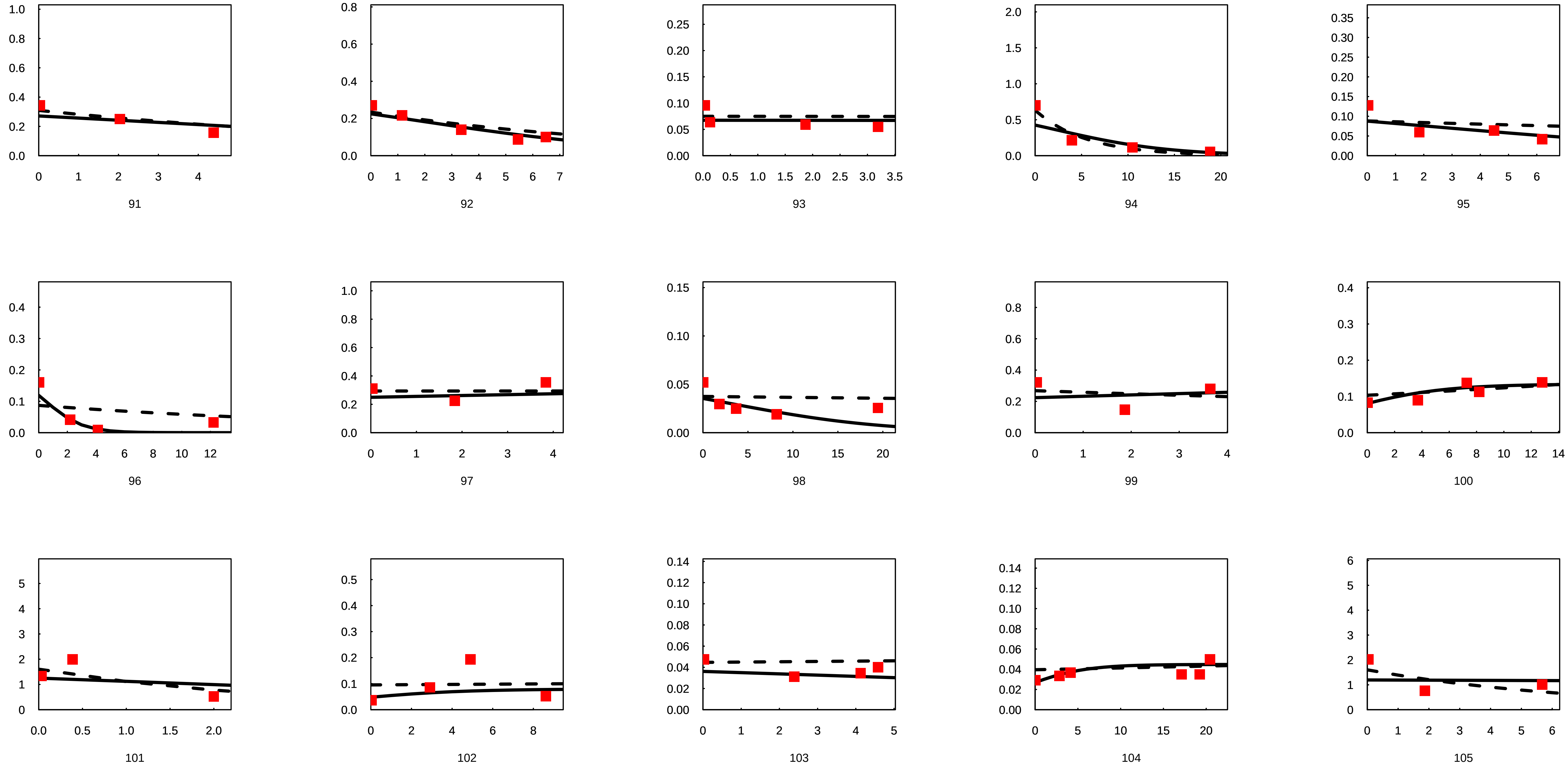
91
92
93
94
95
96
97
98
99
100
101
102
103
104
105

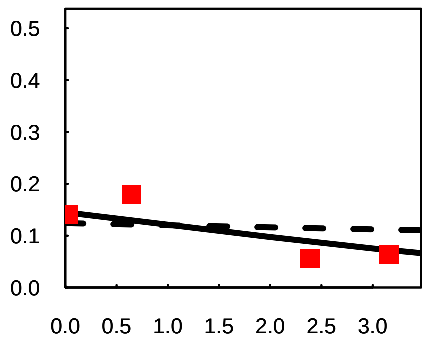

106

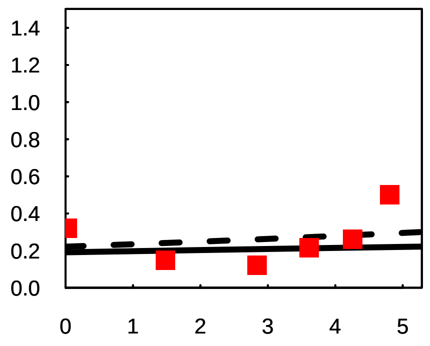

107

Metastasis FOLFIRINOX

Y axis: Tumor volume/10^2 (cm^3)

X axis: Months

line: logistic model

dotted line: exponential model

red square: data

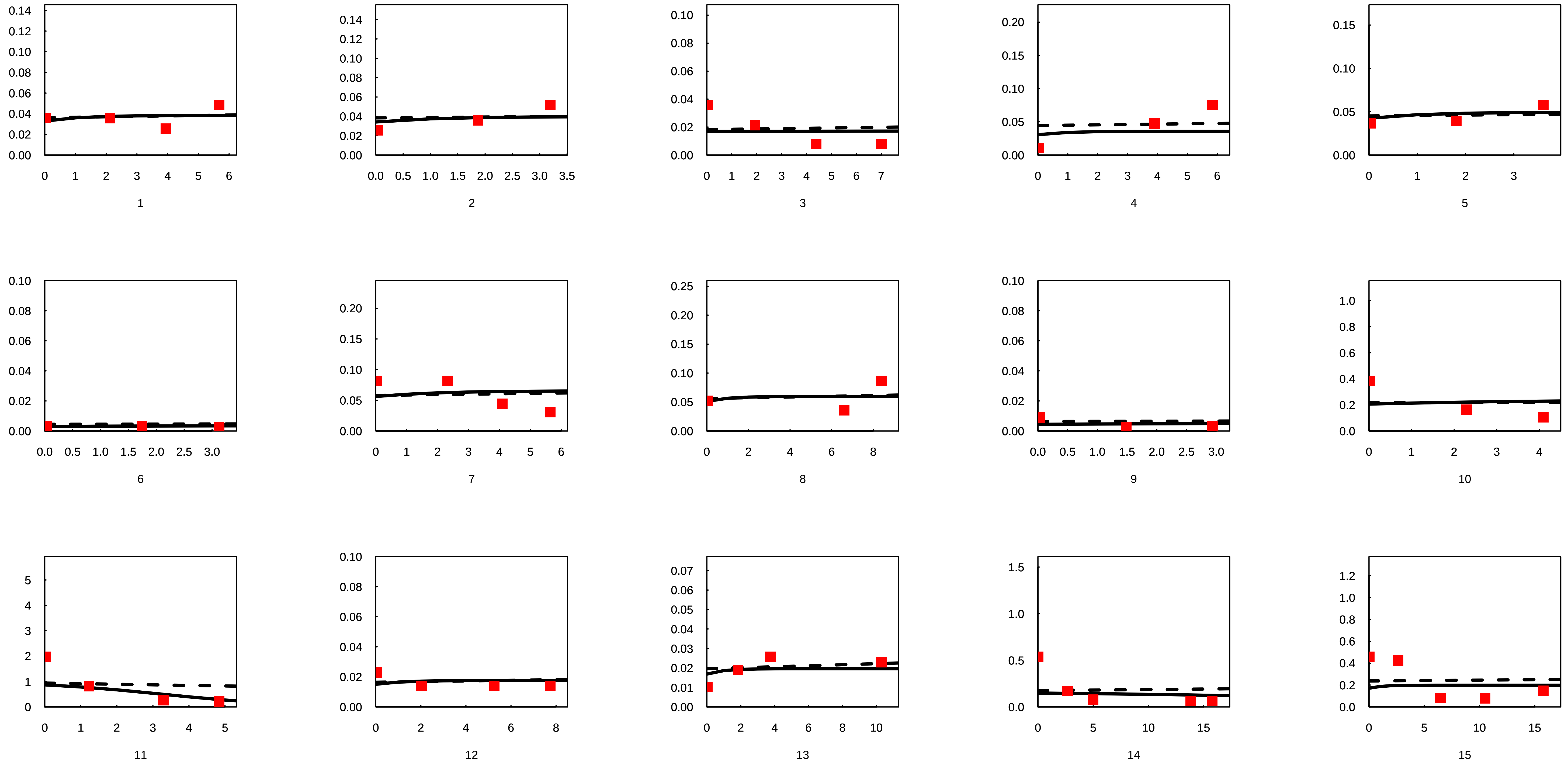
1
0.00 0.02 0.04 0.06 0.08 0.10 0.12 0.14
0 1 2 3 4 5 6
2
0.00 0.02 0.04 0.06 0.08 0.10 0.12 0.14
0.0 0.5 1.0 1.5 2.0 2.5 3.0 3.5
3
0.00 0.02 0.04 0.06 0.08 0.10
0 1 2 3 4 5 6 7
4
0.00 0.05 0.10 0.15 0.20
0 1 2 3 4 5 6
5
0.00 0.05 0.10 0.15
0 1 2 3
6
0.00 0.02 0.04 0.06 0.08 0.10
0.0 0.5 1.0 1.5 2.0 2.5 3.0
7
0.00 0.05 0.10 0.15 0.20
0 1 2 3 4 5 6
8
0.00 0.05 0.10 0.15 0.20 0.25
0 2 4 6 8
9
0.00 0.02 0.04 0.06 0.08 0.10
0.0 0.5 1.0 1.5 2.0 2.5 3.0
10
0.0 0.2 0.4 0.6 0.8 1.0
0 1 2 3 4
11
0 1 2 3 4 5
0 1 2 3 4 5
12
0.00 0.02 0.04 0.06 0.08 0.10
0 2 4 6 8
13
0.00 0.01 0.02 0.03 0.04 0.05 0.06 0.07
0 2 4 6 8 10
14
0.0 0.5 1.0 1.5
0 5 10 15
15
0.0 0.2 0.4 0.6 0.8 1.0 1.2
0 5 10 15

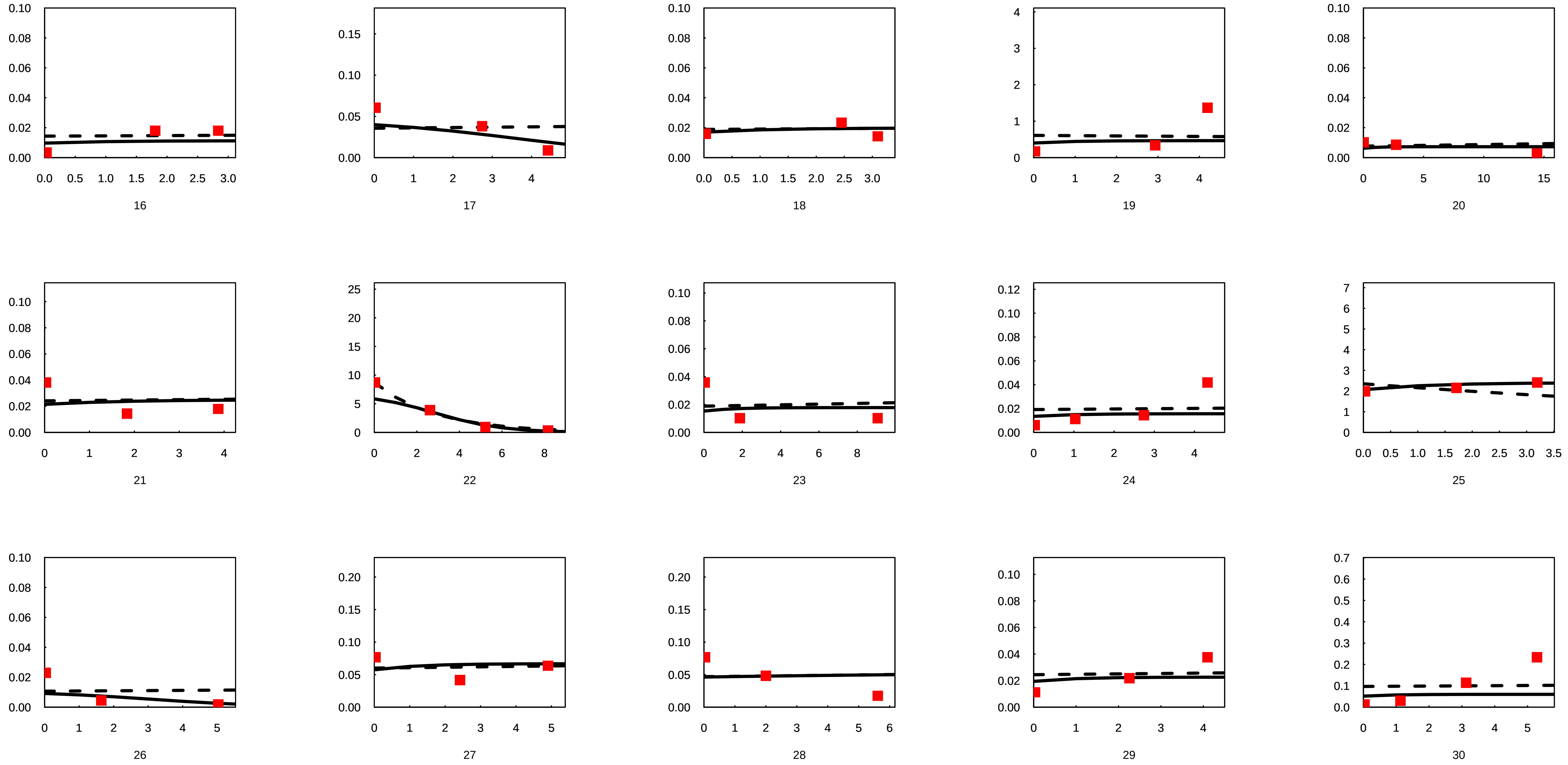
16
17
18
19
20
21
22
23
24
25
26
27
28
29
30

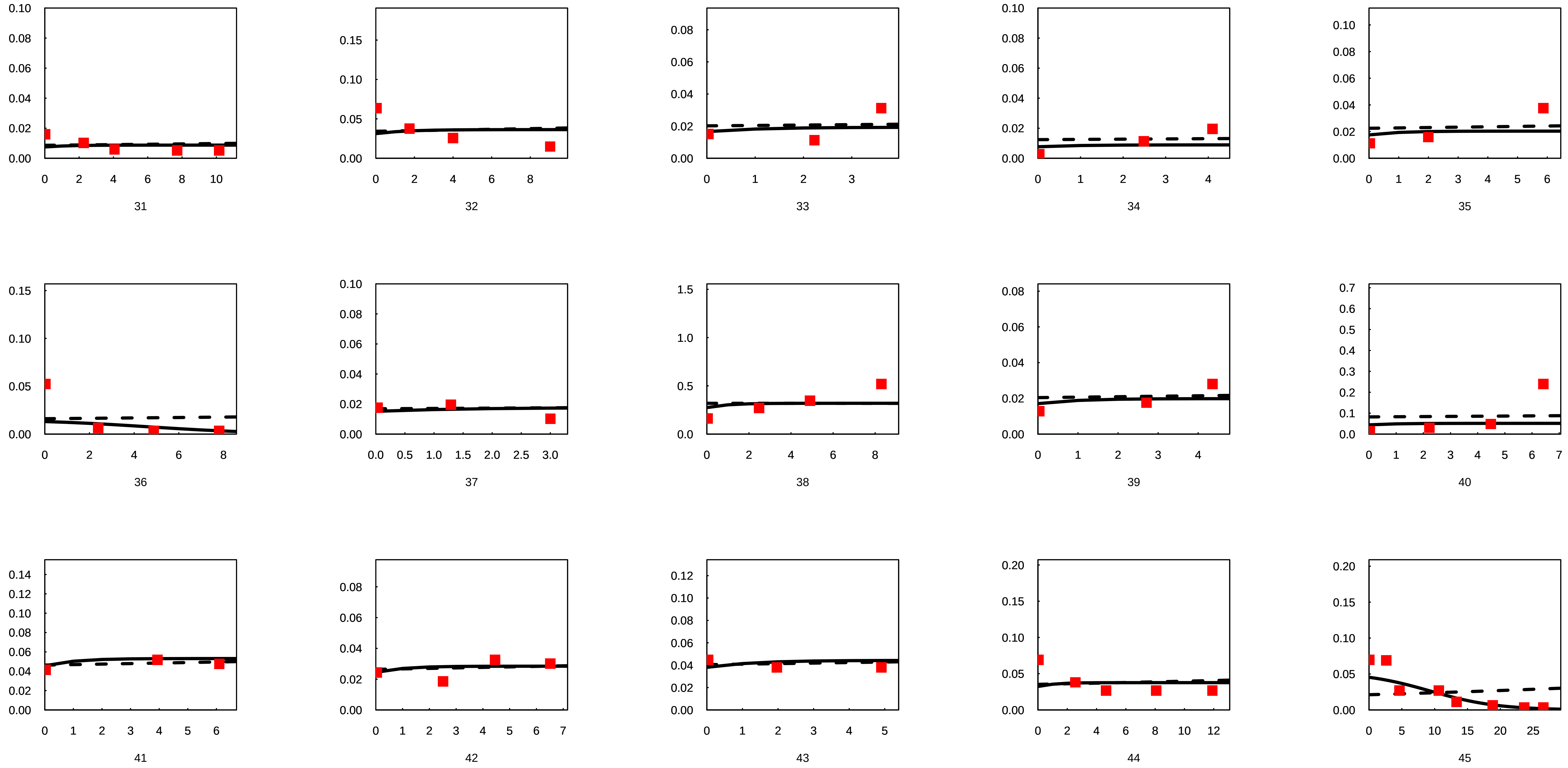
31
32
33
34
35
36
37
38
39
40
41
42
43
44
45

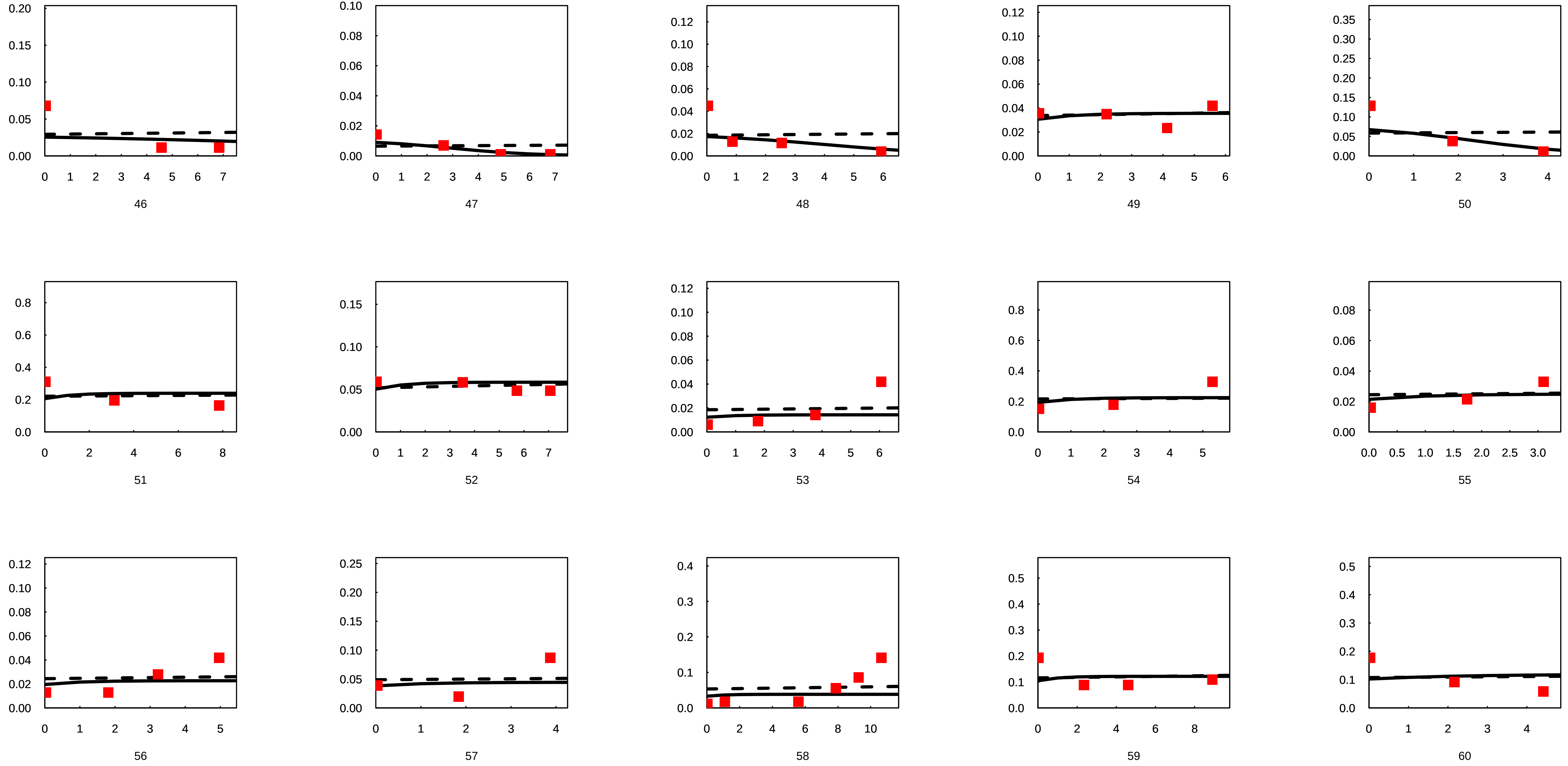
0.20
0.15
0.10
0.05
0.00
0 1 2 3 4 5 6 7
46
0.10
0.08
0.06
0.04
0.02
0.00
0 1 2 3 4 5 6 7
47
0.12
0.10
0.08
0.06
0.04
0.02
0.00
0 1 2 3 4 5 6
48
0.12
0.10
0.08
0.06
0.04
0.02
0.00
0 1 2 3 4 5 6
49
0.35
0.30
0.25
0.20
0.15
0.10
0.05
0.00
0 1 2 3 4
50
0.8
0.6
0.4
0.2
0.0
0 2 4 6 8
51
0.15
0.10
0.05
0.00
0 1 2 3 4 5 6 7
52
0.12
0.10
0.08
0.06
0.04
0.02
0.00
0 1 2 3 4 5 6
53
0.8
0.6
0.4
0.2
0.0
0 1 2 3 4 5
54
0.08
0.06
0.04
0.02
0.00
0.0 0.5 1.0 1.5 2.0 2.5 3.0
55
0.12
0.10
0.08
0.06
0.04
0.02
0.00
0 1 2 3 4 5
56
0.25
0.20
0.15
0.10
0.05
0.00
0 1 2 3 4
57
0.4
0.3
0.2
0.1
0.0
0 2 4 6 8 10
58
0.5
0.4
0.3
0.2
0.1
0.0
0 2 4 6 8
59
0.5
0.4
0.3
0.2
0.1
0.0
0 1 2 3 4
60

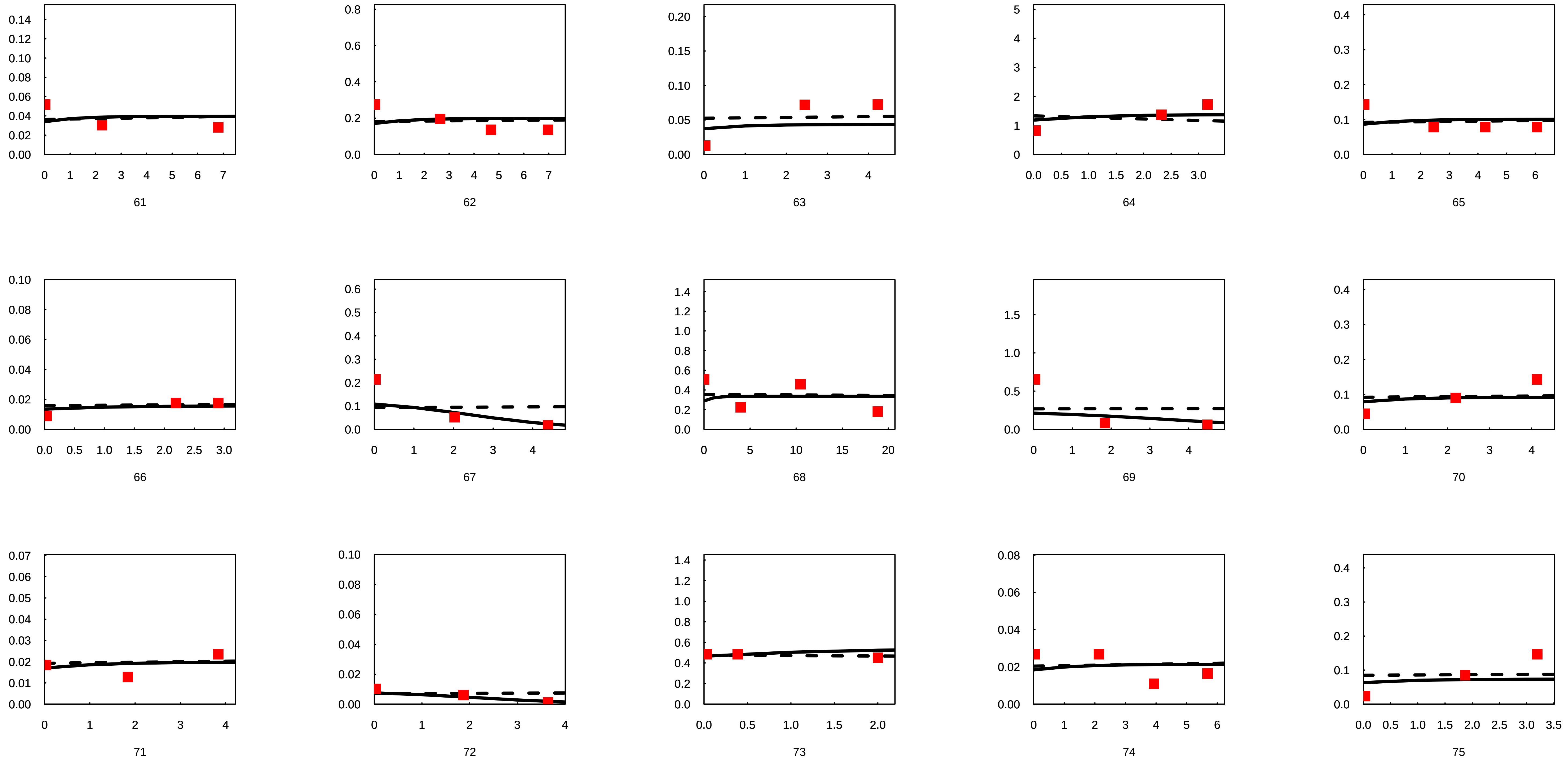
0.14 0.12 0.10 0.08 0.06 0.04 0.02 0.00
0 1 2 3 4 5 6 7
61
0.8 0.6 0.4 0.2 0.0
0 1 2 3 4 5 6 7
62
0.20 0.15 0.10 0.05 0.00
0 1 2 3 4
63
5 4 3 2 1 0
0.0 0.5 1.0 1.5 2.0 2.5 3.0
64
0.4 0.3 0.2 0.1 0.0
0 1 2 3 4 5 6
65
0.10 0.08 0.06 0.04 0.02 0.00
0.0 0.5 1.0 1.5 2.0 2.5 3.0
66
0.6 0.5 0.4 0.3 0.2 0.1 0.0
0 1 2 3 4
67
1.4 1.2 1.0 0.8 0.6 0.4 0.2 0.0
0 5 10 15 20
68
1.5 1.0 0.5 0.0
0 1 2 3 4
69
0.4 0.3 0.2 0.1 0.0
0 1 2 3 4
70
0.07 0.06 0.05 0.04 0.03 0.02 0.01 0.00
0 1 2 3 4
71
0.10 0.08 0.06 0.04 0.02 0.00
0 1 2 3 4
72
1.4 1.2 1.0 0.8 0.6 0.4 0.2 0.0
0.0 0.5 1.0 1.5 2.0
73
0.08 0.06 0.04 0.02 0.00
0 1 2 3 4 5 6
74
0.4 0.3 0.2 0.1 0.0
0.0 0.5 1.0 1.5 2.0 2.5 3.0 3.5
75

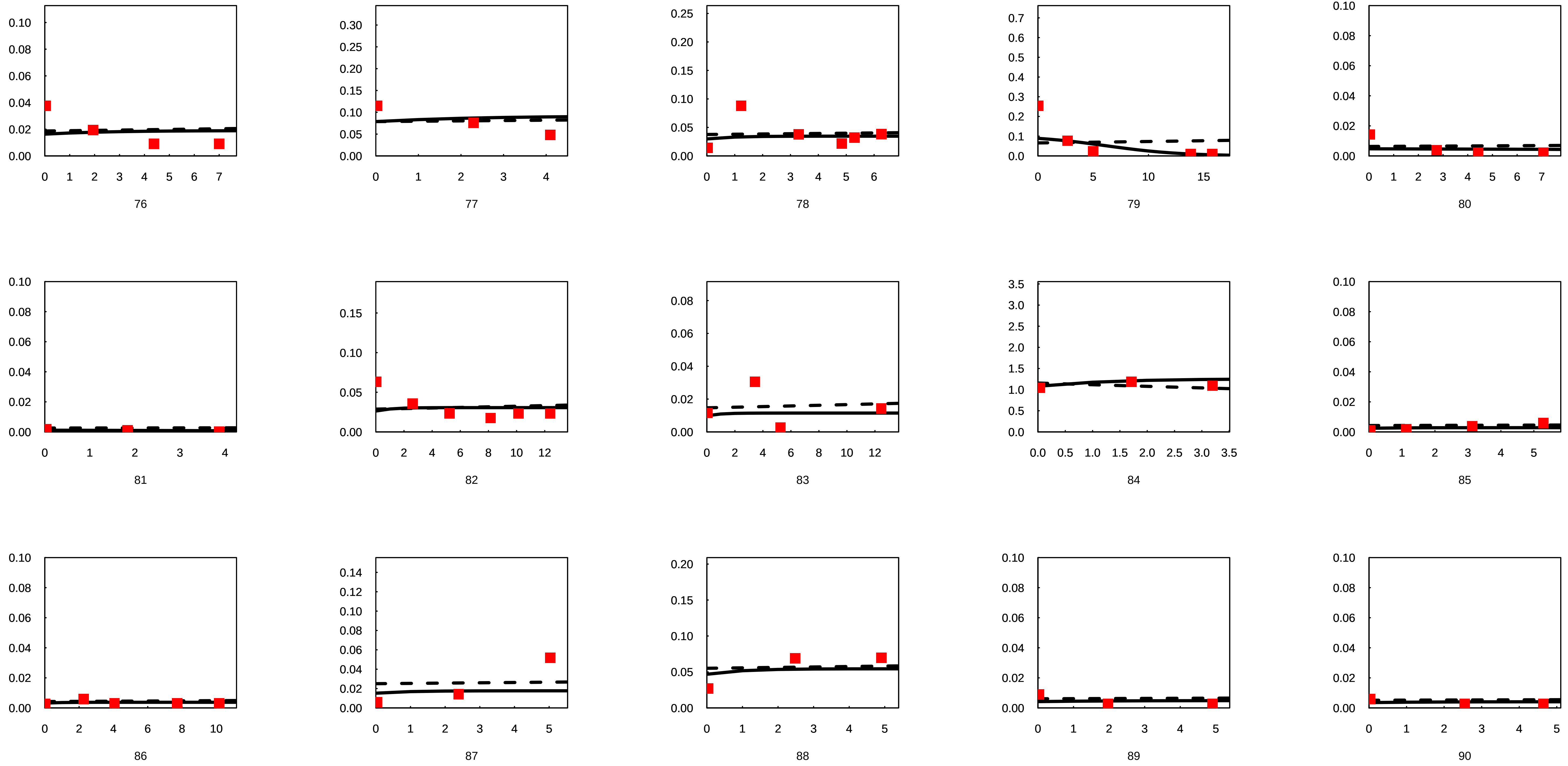
76
77
78
79
80
81
82
83
84
85
86
87
88
89
90

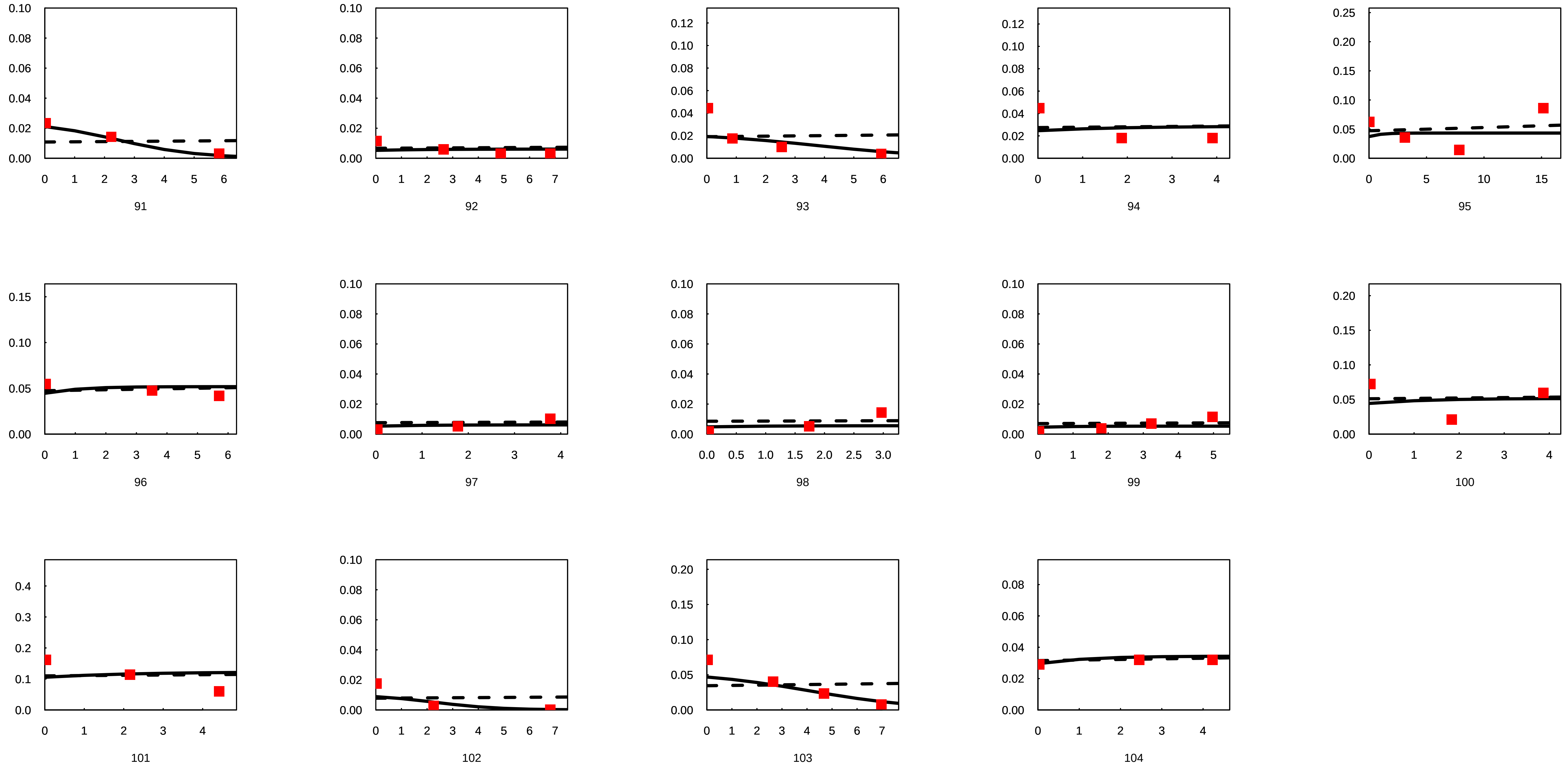
91
92
93
94
95
96
97
98
99
100
101
102
103
104
