# Supplementary material for: Computational modeling of pancreatic cancer patients receiving FOLFIRINOX and gemcitabine-based therapies identifies optimum intervention strategies
Source: PLoS One. 2019 Apr 26;14(4):e0215409. doi: 10.1371/journal.pone.0215409 (PMC6485645; doi:10.1371/journal.pone.0215409)
Supplement: S3 Data — (i)-(iv) Assessments of growth rates, LAI and B by Bayesian estimation using volume measurements of primary tumors in different treatment regimens. (v)-(viii) Assessments of growth rates, MAI, and B by Bayesian estimation using volume measurements of metastatic tumors in different treatment regimens. (DOCX) [file pone.0215409.s003.docx]

**SUPPORTING INFORMATION, DATA 3**

**Table of Contents**

**Estimated parameters for a mixed effects logistic model using volume measurements of**

**i. primary tumors in the absence of treatment Page 2**

**ii. primary tumors during GEM therapy Page 3**

**iii. primary tumors during FFX therapy Page 4**

**iv. primary tumors during GEM+abraxane therapy Page 5**

**v. metastatic tumors in the absence of treatment Page 6**

**vi. metastatic tumors during GEM therapy Page 7**

**vii. metastatic tumors during FFX therapy Page 8**

**viii. metastatic tumor during GEM+abraxane therapy Page 9**

**(i) Estimated parameters for a mixed effects logistic model using volume measurements of primary tumors in the absence of treatment**

**
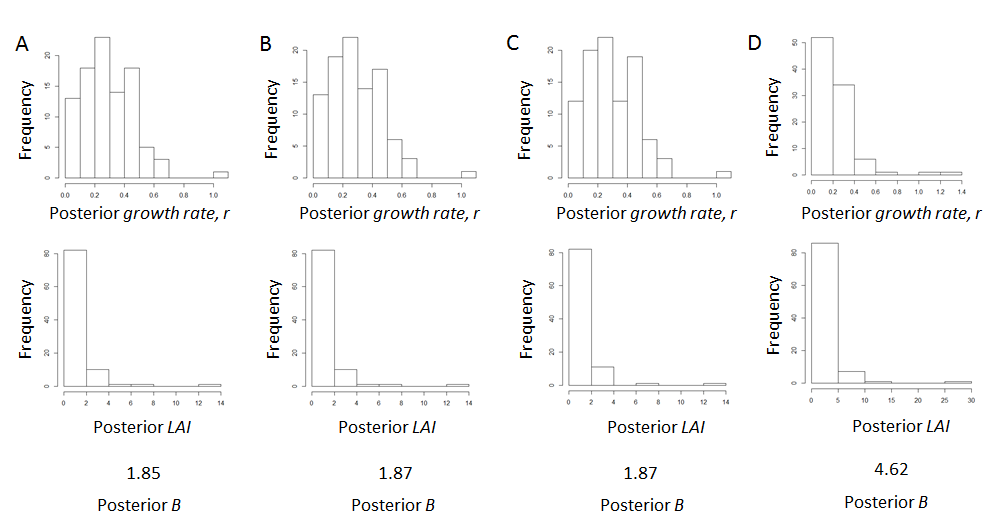
**

(A–D) Posterior distributions of growth rates, *LAI*, and *B* using Bayesian estimation. We conducted sensitivity analyses using different priors. Prior distributions for growth rates were obtained from *N*(0.16, 0.14) based on a previous study (*24*). Prior distributions for *LAI* and *B* were obtained from (A) *LN*(0, 10) and *LN*(0, 10), respectively; (B) *LN*(0, 10) and *LN*(0, 1.0), respectively; and (C) *LN*(0, 100) and *LN*(0, 10), respectively. Here *N*(*µ*, *σ*) represents the normal distribution with mean *µ* and variance *σ*, and *LN*(*µ*, *σ*) represents the lognormal distribution with parameters *µ* and *σ*. (D) Prior distributions for growth rates, *LAI*, and *B* are *N*(0.16, *σ*) with *σ*~gamma(2, 2/0.14^1/2^), *LN*(0, 10) and *LN*(0, 10), respectively.

**(ii) Estimated parameters for a mixed effects logistic model using volume measurements of primary tumors during GEM therapy**

**
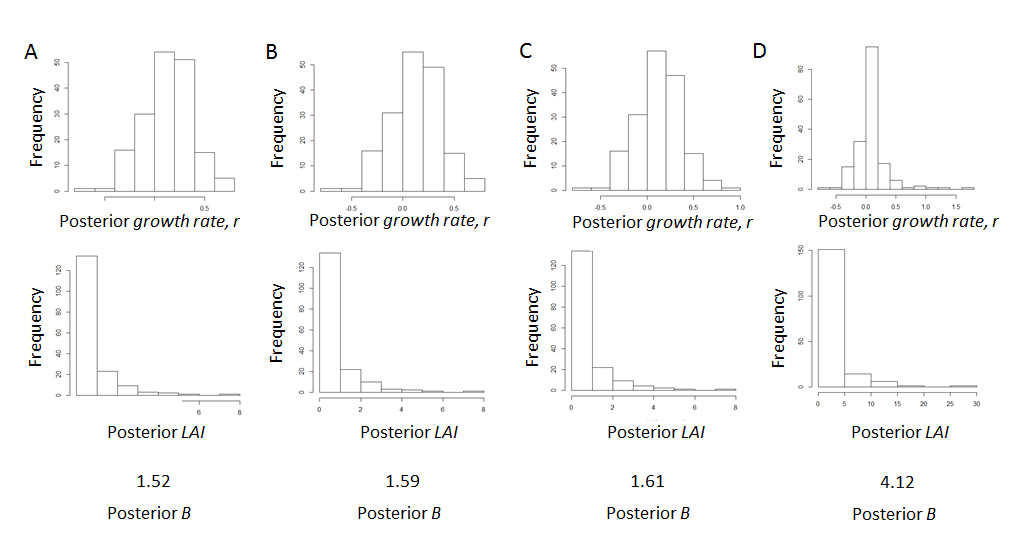
**

(A–D) Posterior distributions of growth rates, *LAI*, and *B* using Bayesian estimation. We conducted sensitivity analyses using different priors. Prior distributions for growth rates, *LAI*, and *B* are the same as those described in **Data (i)**.

**(iii) Estimated parameters for a mixed effects logistic model using volume measurements of primary tumors during FFX therapy**

**
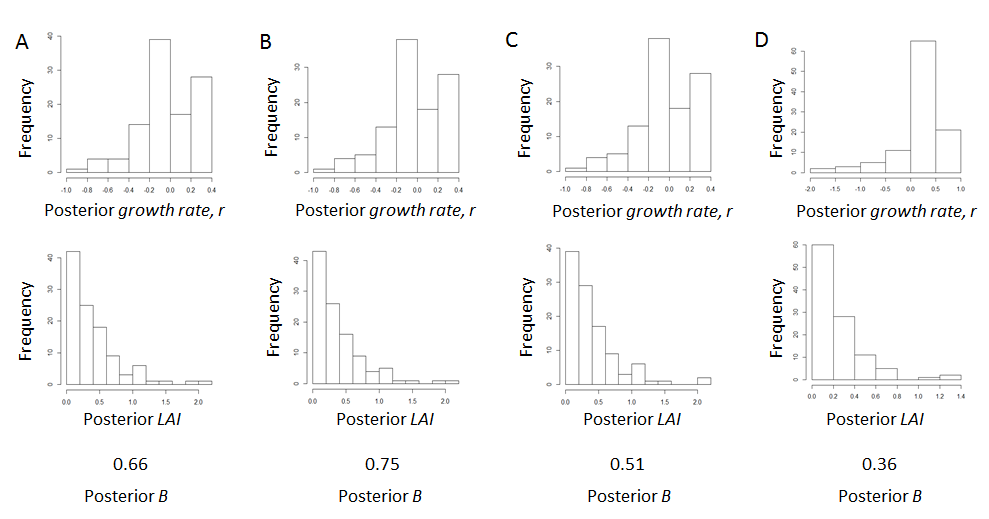
**

(A–D) Posterior distributions of growth rates, *LAI*, and *B* using Bayesian estimation. We conducted sensitivity analyses using different priors. Prior distributions for growth rates, *LAI*, and *B* are the same as those described in **Data (i).**

**(iv) Estimated parameters for a mixed effects logistic model using volume measurements of primary tumors during GEM+abraxane therapy**

**
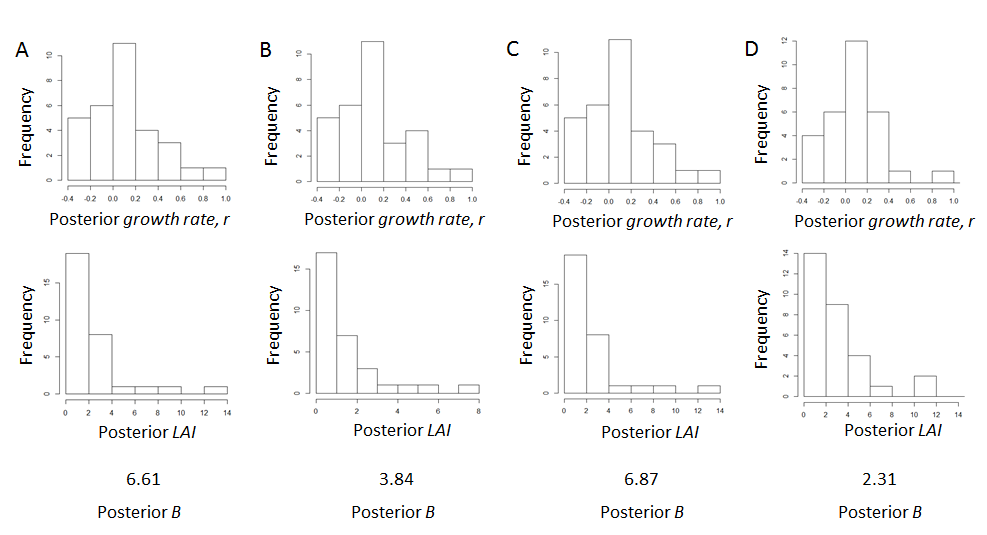
**

(A–C) Posterior distributions of growth rates, *LAI*, and *B* using Bayesian estimation. We conducted sensitivity analyses using different priors. Prior distributions for growth rates, *LAI*, and *B* are the same as those described in **Data (i)**.

**(v) Estimated parameters for a mixed effects logistic model using volume measurements of metastatic tumors in the absence of treatment**

**
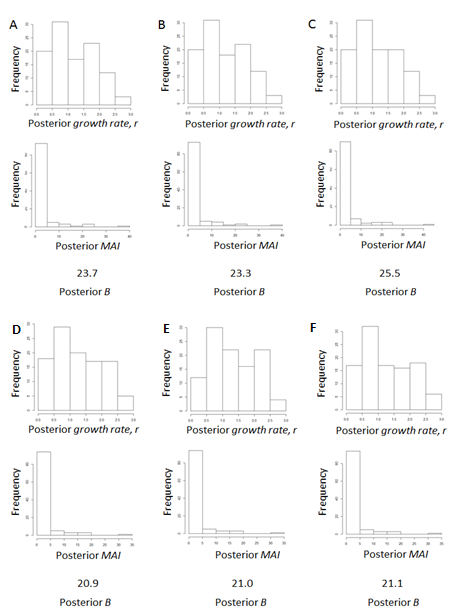
**

(A–C) Posterior distributions of growth rates, *MAI*, and *B* using Bayesian estimation. We conducted sensitivity analyses using different priors. Prior distributions for growth rates were obtained from *N*(0.58, 2.72) that is based on a previous study (*24*). Prior distributions for *MAI* and *B* were obtained from (A) *LN*(0, 10) and *LN*(0, 10), respectively; (B) *LN*(0, 10) and *LN*(0, 1.0), respectively; and (C) *LN*(0, 100) and *LN*(0, 10), respectively. (D–F) Posterior distributions of growth rates, *MAI*, and *B* using Bayesian estimation. Prior distributions for growth rates were *N*(*µ*, *σ*) with *µ*~ *N*(0.58, 1.0) and *σ*~gamma(2, 2/2.52^1/2^), *LN*(0, 10) and *LN*(0, 10). Prior distributions for *MAI* and *B* were obtained from (A) *LN*(0, 10) and *LN*(0, 1.0), respectively; (B) *LN*(0, 10) and *LN*(0, 1.0), respectively; and (C) *LN*(0, 100) and *LN*(0, 10), respectively.

**(vi) Estimated parameters for a mixed effects logistic model using volume measurements of metastatic tumors during GEM therapy**

**
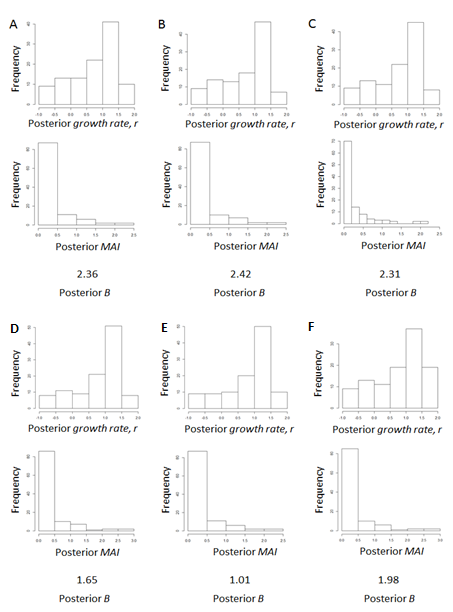
**

(A–F) Posterior distributions of growth rates, *MAI*, and *B* using Bayesian estimation. We conducted sensitivity analyses using different priors. Prior distributions for growth rates, *MAI* and *B* are the same as those in **Data (v)**.

**(vii) Estimated parameters for a mixed effects logistic model using volume measurements of metastatic tumors during FFX therapy**

**
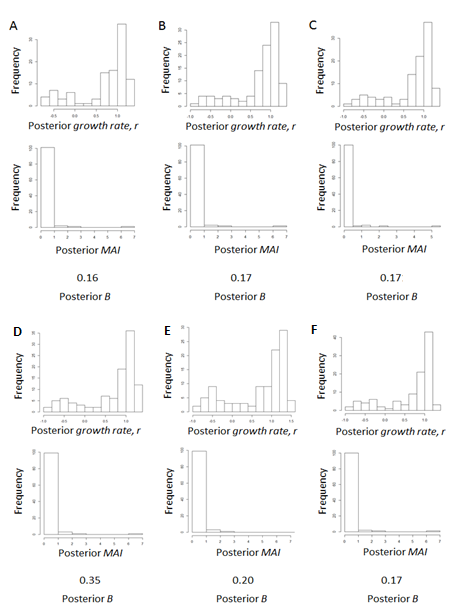
**

(A–F) Posterior distributions of growth rates, *MAI*, and *B* using Bayesian estimation. We conducted sensitivity analyses using different priors. Prior distributions for growth rates, *MAI*, and *B* are the same as those described in **Data (v)**.

**(viii) Estimated parameters for a mixed effects logistic model using volume measurements of metastatic tumors during GEM+abraxane therapy.**

**
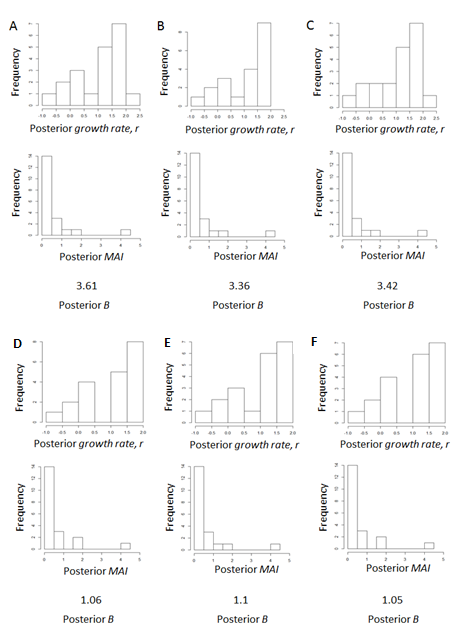
**

(A–F) Posterior distributions of growth rates, *MAI*, and *B* using Bayesian estimation. We conducted sensitivity analyses using different priors. Prior distributions for growth rates, *MAI*, and *B* are the same as those in **Data (v).**
