# Supplementary material for: Computational modeling of pancreatic cancer patients receiving FOLFIRINOX and gemcitabine-based therapies identifies optimum intervention strategies
Source: PLoS One. 2019 Apr 26;14(4):e0215409. doi: 10.1371/journal.pone.0215409 (PMC6485645; doi:10.1371/journal.pone.0215409)
Supplement: S1 Doc — The descriptions include an explanation or discussion regarding (a) Clinical cohorts, (b) Computational modeling of PDAC progression, (c) Computer simulations before diagnosis, (d) Computer simulations after diagnosis, (e) Three-step branching process with different growth models, (f) Statistical analysis, (g) Computational studies for optimal treatment, (h) Supplementary discussion, (i) Stan code for Bayesian inference, and (j) Evaluation of the accuracy of LAI. (DOCX) [file pone.0215409.s004.docx]

**S1 Doc (a). Clinical cohorts**

We analyzed data from a total of 1,089 patients (599 men, 490 women) who were treated for PDAC at Massachusetts General Hospital (MGH) between October 2002 and September 2015. Baseline characteristics of the study population are provided in **Table 1**. Among our patient cohort, three or more time points of sequential volume measurements were available for 693 patients with 742 tumors under homogeneous treatment conditions. We analyzed data on the sizes of primary and metastatic tumors from 93 and 95 patients, respectively, who did not receive any treatment during the time period when volume measurements were performed; 173 and 101 patients, respectively, who received GEM; 31 and 20 patients, respectively, who received GEM+nab-paclitaxel; and 107 and 73 patients, respectively, who received FFX (**Fig 1C** and **Table 1**). A radiologist specializing in treating pancreatic cancer measured longitudinal primary and metastatic tumor sizes and metastatic burden. Note that our clinical data are unique – longitudinal imaging data without any treatment can rarely be obtained in PDAC because most patients receive treatment soon after diagnosis. Furthermore, longitudinal imaging data of patients who received homogeneous treatment is usually limited because patients’ follow up durations are generally short due to the severity of the disease (Haeno et al., 2012). The second cohort contained information on 73 PDAC patients with surgically unresectable disease who were treated between April 2008 and March 2016 at Osaka Medical College (OMC) in Japan (**Fig 1D** and **Table 1**). Data collection and analysis were approved by the Ethics Committees on Clinical Investigation of both MGH and OMC. Methods were carried out in accordance with the approved guidelines. Chemotherapeutic drug exposure was determined according to body surface area-based dosing strategies, which are 1000mg/m2 for gemcitabine and 400mg/m2, 200mg/m2, 85mg/m2, 180mg/m2, for fluorouracil, leucovorin, oxaliplatin, and irinotecan in FFX, respectively. In the clinic, standard administration strategies for these drugs do not account for area under curve (AUC) and plasma/serum concentrations.

**S1 Doc (b). Computational modeling of PDAC progression**

We designed a novel computational model of PDAC progression using a three cell type logistic branching process starting from a single cell localized in the primary site (**Fig 1E and S1 Fig**). Our model is based on findings that metastatic efficiency is determined by (epi)genetic alterations that arise during the clonal expansion of PDAC. In the model, two types of cells exist within the primary site: there is the tumor-initiating population, which has not yet evolved the potential to disseminate and consists entirely of so-called type-0 cells. Type-0 cells can give rise to type-1 cells, which also reside in the primary site but have evolved the ability to metastasize, which in turn can produce type-2 cells by leaving the primary site and establishing a new metastatic colony elsewhere. The growth rates of all cell types decrease with increasing tumor size such that the number of cells is given by an increasing curve that converges to a maximum size (**S1 Fig**). We define this maximum tumor size, i.e. the carrying capacity of the model, as Local Advancement Index (LAI) at the primary site and as Metastatic Advancement Index (MAI) at a metastatic site (**Fig 1E**). By incorporating LAI and MAI into the model, we capture the divergence in sizes of the eventual tumor mass among patients, so that both LAPC and widespread metastatic PDAC disease can be described by one integrated framework. We consider situations in which metastatic ability is a consequence of a single (epi)genetic change, such as genetic inactivation of *SMAD4*, high expression levels of *RUNX3*, or epigenetic reprogramming (Iacobuzio-Donahue et al., 2009; McDonald et al., 2017; Whittle et al., 2015; Yachida et al., 2010). The metastasis-enabling event arises with probability *u* per type-0 cell division and creates a type-1 cell. In the context of the model, type-0 cells divide at rate *r_0_*(1-(*w+x*)*/LAI*) and die at rate *d_0_* per unit time, where *w*, *x* denote the number of type-0 and type-1 cells, respectively. Type-1 cells divide and die at rates *r_1_*(1-(*w+x*)*/LAI*) and *d*_1_ per unit time, respectively. Type-0 and -1 cells share the same LAI because both cell types reside in the primary tumor. During each elementary time step of the stochastic process, cells may divide with a possibility of an alteration occurring (which enables them to metastasize), die, or leave the primary site to initiate a colony elsewhere. Cells with metastatic ability can establish a metastatic site at another location; these sites start from a single metastasizing cell, and each export event results in a new metastatic colony. The integrated rate of leaving the primary site and founding a new metastasis at a distant site is denoted by *q* per unit time. Once a type-1 cell has migrated to a new distant site, it is referred to as a type-2 cell. Again, the growth rate of type-2 cells decreases with increasing metastasis size. At the *i*-th metastatic site, type-2 cells may go extinct or divide with a division and death rate of *r_2_*(1-*y_i_/MAI_i_*) and *d*_2_ per unit time, respectively. Here, *y_i_* and *MAI_i_* denote the number of cells and the MAI of the *i*-th metastatic site, respectively. The total number of sites where tumor cells have founded metastases until a particular time *t* is denoted by *I(t)*. Based on its stochastic nature, the model can account for the situation in which PDAC cells acquire the ability to metastasize very early on, with the majority of cells within a primary tumor having the ability to metastasize, and the situation in which this ability arises late during tumor progression.

**S1 Doc (c). Computer simulations before diagnosis**

We performed exact computer simulations of the stochastic process outlined above. The numbers of the three types of cells – type-0, type-1, and type-2 cells in the *i*-th metastatic site – are denoted by *w*, *x*, and *y_i_*, respectively. A change in *w*, *x*, and *y_i_* can occur by cell division with the possibility of accumulating the metastasis-enabling alteration, cell death, or departure of a cell from the primary site. Initially, there is one type-0 cell, *w* = 1, and no type-1 or -2 cells. Our model considers all possible events— production and death of a type-0, -1, or -2 cell, and metastasis of a type-1 cell to a new site. The probability of each event is given by its rate normalized by the sum of the rates of all possible events, given by

*Γ*= [*r_0_*{1-(*w+x*)*/LAI*}+*d_0_*]*w*+ [*r_1_*{1-(*w+x*)*/LAI*}*+d_1_*+*q*]*x* +Σ*_i_*{*r_2_*(1-*y_i_/MAI_i_*)+*d_2_*}*y_i_*.

The timing of one event is given by a distribution with mean 1/*Γ*. Each metastatic site has a distinct MAI, even within the same patient (Iacobuzio-Donahue et al., 2009). The transition probabilities between states in the stochastic simulation are determined as follows: the probability that the number of each cell type increases by one is given by

Pr[(*w*,*x*,*y_1_*,*y_2_*,…,*y_I_*)→ (*w*+1,*x*,*y_1_*,*y_2_*,…,*y_I_*)] = *r_0_w*{1-(*w+x*)*/LAI*}(1-*u*)/*Γ*

Pr[(*w*,*x*,*y_1_*,*y_2_*,…,*y_I_*)→(*w*,*x*+1,*y_1_*,*y_2_*,…,*y_I_*)] = (*r_0_wu* + *r_1_x*){1-(*w+x*)*/LAI*}/*Γ*

Pr[(*w*,*x*,*y_1_*,*y_2_*,..,*y_i_*,..,*y_I_*)→(*w*,*x*,*y_1_*,*y_2_*,.. *y_i_*+1,..,*y_I_*)] = *r_2_y_i_*(1-*y_i_/MAI_i_*)/*Γ* (4)

Export of a type-1 cell to a new metastatic site increases the number of metastatic cells by one at a new site *I+1* and decreases the number of type-1 cells by one; the probability of this event is given by

Pr[(*w*,*x*,*y_1_*,*y_2_*,…,*y_I_*)→(*w*,*x*-1,*y_1_*,*y_2_*,…,*y_I_*,1)] = *xq*/*Γ* (5)

The probabilities that the numbers of type-0, -1, and -2 cells in the *i*-th metastatic site decrease by one are given by

Pr[(*w*,*x*,*y_1_*,*y_2_*,…, *y_I_*)→(*w-*1,*x*,*y_1_*,*y_2_*,…,*y_I_*)]=*d_0_w*/*Γ*

Pr[(*w*,*x*,*y_1_*,*y_2_*,…, *y_I_*)→(*w*,*x-*1,*y_1_*,*y_2_*,…,*y_I_*)]=*d_1_x*/*Γ*

Pr[(*w*,*x*,*y_1_*,*y_2_*,..,*y_i_*,..,*y_I_*)→(*w*,*x*,*y_1_*,*y_2_*,..,*y_i_-*1,..,*y_I_*)]=*d_2_y_i_*/*Γ* (6)

Based on the framework described in equations (4)–(6), we performed independent runs of the stochastic process either until all cells go extinct, *w* = *x* = Σ*_i_y_i_* = 0, or until the total cell number reaches the final size, *w*+*x*+Σ*_i_y_i_*=*M_diag_*, leading to diagnosis of the disease. All three cell types contribute to the size at diagnosis based on the evidence that metastatic disease with unknown primary is diagnosed in rare cases (Ayoub et al., 1998). We converted the tumor volume determined in patients to cell numbers with the assumption of a spherical shape (10^9^ cells occupy a volume of 1 cm^3^). On the basis of the presence of extensive fibrosis as a defining feature of PDAC, we assumed that there are 80% stromal cells in a pancreatic tumor (Kadaba et al., 2013). The tumor size distribution at diagnosis was 10^N(9.47,0.29)^, which was estimated from the MGH patient cohort. The (epi)genetic alteration rate and metastatic rate were obtained from a previous study (Haeno et al., 2012). In the context of our model, the growth and death rates of type-0 and type-1 cells are the same based on the fact that there is no evidence that (epi)genetic alterations in metastasis-related genes increase a cell’s fitness, and given our inability to estimate these rates separately.

**S1 Doc (d). Computer simulations after diagnosis**

Once a tumor has been diagnosed with a population size of *M_diag_*, we consider therapeutic options. We implemented different treatment strategies based on the guidelines for PDAC treatment provided by the National Comprehensive Cancer Network (NCCN) in the United States (National Comprehensive Cancer Network, 2016). If no metastases are detected at diagnosis, a simulated case is regarded as non-metastatic and the patient becomes a candidate for surgery with or without adjuvant CTx, or CRTx. The effect of resection in the model is to remove a fraction (1-*ε*) (0 ≤*ε*≤ 1) of the primary tumor – i.e. both type-0 and type-1 cells (Haeno et al., 2012). Thus the remnant tumor volume in a patient becomes *ε* of the primary tumor. The parameter *ε* is randomly chosen from [10^-5^, 10^-1^] based on clinical evidence that the resection margin is classified as R0 (microscopic tumor clearance), R1 (microscopic residual tumor), or R2 (macroscopic tumor tissue on the margin) in surgical and pathological oncology (Butturini et al., 2008). Surgical resection is performed at the time of diagnosis. If any metastases are detected, a simulated patient receives CTx without surgical resection. Here a metastasis is detected if its diameter is larger than 1mm (Haeno et al., 2012; Moffitt et al., 2015). The growth rates of cells during CTx (GEM-based therapies or FFX) were estimated from the corresponding clinical data of patients during treatment with the respective drug. Specifically, we utilized three or more time points of sequential volume measurements of primary and metastatic sites during each treatment. We fitted a logistic model, which was selected as the best model, to the data and then estimated growth rates during each CTx (**S1 Data)**. In this study, we assumed that drugs reversibly modify growth rates at the time of treatment discontinuation. For radiotherapy, the conventional long course 3D-CRT was considered. The effect of conventionally fractioned RTx was determined using the Linear-Quadratic model (Halperin et al., 2008). In this framework, the surviving fraction of radiated cells is given by *e^-(ωD+ξD2)^*, where *ω* and *ξ* are constants and *D* is the dose. We considered *ω*= 10*ξ*, which is a well-accepted quantity for cancer cells (Halperin et al., 2008), that RTx with a total dose of 54 Gy was delivered in 30 fractions, and that D is 1.8 Gy per fraction. In our computational framework, we defined death of a patient to occur when the total number of tumor cells reaches a certain size (*M_death_)*, based on previously analyzed rapid autopsy data of pancreatic cancer patients (Iacobuzio-Donahue et al., 2009). Survival duration was defined as overall survival (OS) after diagnosis.

**S1 Doc (e). Three-step branching process with different growth models**

Using computational simulations, we investigated the three cell type branching process with different growth models: (i) logistic, (ii) linear, and (iii) exponential models. Computer simulations and analyses were conducted in the same manner as explained in **S1 Doc (c), (d), and (f)**. With the use of mixed effects models (see **S1 Doc(f)**), we first estimated growth rates using each growth model by fitting our time series tumor volume data in the absence of treatment to equations (1)–(3) shown in the **Materials and Methods,** respectively. Using the estimated growth rates, we then performed exact stochastic computer simulations as follows:

(i) The logistic growth model. The simulation algorithm for this model is described in **S1 Doc (c).**

(ii) The linear growth model. For this simulation algorithm, the numbers type-0, type-1, and type-2 cells in the *i*-th metastatic site are denoted by *w_lin_*, *x_lin_*, and *y_lin_i_*, respectively. These cells divide at rate *r_0_lin_*, *r_1_lin_*, and *r_2_lin_*, and die at rate *d_0_lin_*, *d_1_lin_*, and *d_2_lin_* per time unit. Initially there is one type-0 cell, *w_lin_* =1, and *x_lin_*=*y_lin_i_*=0 for all *i*. The probability of each event is proportional to its rate normalized by the sum of the rates of all possible events, given by *Γ_lin_*=(*r_0_lin_*+*d_0_lin_*)+(*r_1_lin_*+*d_1_lin_*+*q*)+ Σ*_i_*(*r_2_lin_*+*d_2_lin_*). The timing of each event is given by a distribution with mean 1/*Γ_lin_*. The transition probabilities between states of the stochastic process are determined as follows. The probability that the number of type-0 cells increases by one is given by

Pr[(*w_lin_*,*x_lin_*,*y_lin_1_*,…,*y_lin_I_*)→ (*w_lin_*+1,*x_lin_*, *y_lin_1_*,…,*y_lin_I_*)] = *r_0_lin_*(1-*u*)/*Γ_lin_*

Pr[(*w_lin_*,*x_lin_*,*y_lin_1_*,…,*y_lin_I_*)→(*w_lin_*,*x_lin_*+1,*y_lin_1_*,…,*y_lin_I_*)] = (*r_1_lin_*+ *r_0_lin_u*)/*Γ_lin_*

Pr[(*w_lin_*,*x_lin_*,*y_lin_1_*,..,*y_lin_i_*,..,*y_lin_I_*)→(*w_lin_*,*x_lin_*,*y_lin_1_*,.. *y_lin_i_*+1,..,*y_lin_I_*)] = *r_2_lin_*/*Γ_lin_* (7)

Export of a type-1 cell to a new metastatic site increases the number of metastatic cells by one at a new site *I+1* and decreases the number of type-1 cells by one; the probability of this event is given by

Pr[(*w_lin_*,*x_lin_*,*y_lin_1_*,…,*y_lin_I_*)→(*w_lin_*,*x_lin_*-1,*y_lin_1_*,…,*y_lin_I_*,1)] = *q*/*Γ_lin_* (8)

The probabilities that the numbers of type-0, -1, and -2 cells in the *i*-th metastatic site decrease by one are given by

Pr[(*w_lin_*,*x_lin_*,*y_lin_1_*,…,*y_lin_I_*)→(*w_lin_-*1,*x_lin_*,*y_lin_1,_*…,*y_lin_I_*)]= *d_0_lin_*/*Γ_lin_*

Pr[(*w_lin_*,*x_lin_*,*y_lin_1_*,…,*y_lin_I_*)→(*w_lin_*,*x_lin_-*1,*y_lin_1,_*…,*y_lin_I_*)]=*d_1_lin_*/*Γ_lin_*

Pr[(*w_lin_*,*x_lin_*,*y_lin_1_*,..,*y_lin_i_*,..,*y_lin_I_*)→(*w_lin_*,*x_lin_*,*y_lin_1_*,..,*y_lin_i_*-1,..,*y_lin_I_*)]= *d_2_lin_*/*Γ_lin_* (9)

(iii) The exponential growth model. For this simulation algorithm, the numbers of type-0, type-1, and type-2 cellsin the *i*-th metastatic site are denoted by *w_exp_*, *x_exp_*, and *y_exp_i_*, respectively. These cells divide at rate *r_0_exp_*, *r_1_exp_*, and *r_2_exp_*, and die at rate *d_0_exp_*, *d_1_exp_*, and *d_2_exp_* per time unit. Initially there is one type-0 cell, *w_exp_*=1, and *x_exp_* =*y_exp_i_*=0 for all *i*. The probability of each event is proportional to its rate normalized by the sum of the rates of all possible events, given by *Γ_exp_*=(*r_0_exp_*+*d_0_exp_*)*w_exp_*+(*r_1_exp_*+*d_1_exp_*+*q*)*x_exp_*+Σ*_i_*(*r_2_exp_*+*d_2_exp_*)*y_exp_i_*. The timing of each event is given by a distribution with mean 1/*Γ_exp_*. The transition probabilities between states of the stochastic process are determined as follows. The probability that the number of type-0 cells increases by one is given by

Pr[(*w_exp_*,*x_exp_*,*y_exp_1_*,…,*y_exp_I_*)→ (*w_exp_*+1,*x_exp_*,*y_exp_1_*,…,*y_exp_I_*)] = *r_0_exp_w_exp_*(1-*u*)/*Γ_exp_*

Pr[(*w_exp_*,*x_exp_*,*y_exp_1_*,…,*y_exp_I_*)→(*w_exp_*,*x_exp_*+1,*y_exp_1_*,…,*y_exp_I_*)] = (*r_1_exp_x_exp_*+ *r_0_exp_w_exp_u*)/*Γ_exp_*

Pr[(*w_exp_*,*x_exp_*,*y_exp_1_*,..,*y_exp_i_*,..,*y_exp_I_*)→(*w_exp_*,*x_exp_*,*y _exp_1_*,.. *y_exp_i_*+1,..,*y_exp_I_*)] = *r_2_exp_y_exp_i_*/*Γ_exp_* (10)

Export of a type-1 cell to a new metastatic site increases the number of metastatic cells by one at a new site *I+1* and decreases the number of type-1 cells by one; the probability of this event is given by

Pr[(*w_exp_*,*x_exp_*,*y_exp_1_*,…,*y_exp_I_*)→(*w_exp_*,*x_exp_*-1,*y_exp_1_*,…,*y_exp_I_*,1)] = *x_exp_q*/*Γ_exp_* (11)

The probabilities that the numbers of type-0, -1, and -2 cells in the *i*-th metastatic site decrease by one are given by

Pr[(*w_exp_*,*x_exp_*,*y_exp_1_*,…,*y_exp_I_*)→(*w_exp_*-1,*x_exp_*,*y_exp_1_*,…,*y_exp_I_*)]= *d_0_exp_w_exp_*/*Γ_exp_*

Pr[(*w_exp_*,*x_exp_*,*y_exp_1_*,…,*y_exp_I_*)→(*w_exp_*,*x_exp_*-1,*y_exp_1_*,…,*y_exp_I_*)]=*d_1_exp_x_exp_*/*Γ_exp_*

Pr[(*w_exp_*,*x_exp_*,*y_exp_1_*,..,*y_exp_i_*,..,*y_exp_I_*)→(*w_exp_*,*x_exp_*,*y _exp_1_*,.. *y_exp_i_*-1,..,*y_exp_I_*)]= *d_2_exp_y_exp_i_*/*Γ_exp_* (12)

For each growth model, we performed independent runs of the stochastic process either until all cells go extinct, or until the total cell number reaches the final size, *w_exp_*+*x_exp_*+Σ*_i_y_exp_i_*=*M_diag_*, leading to diagnosis. Treatment was administrated in the same manner as described in **S1 Doc(d)** and simulations were continued until death**.** We performed 100 simulations for each model until death.

**S1 Doc (f). Statistical analysis**

In this study, we explored the use of mixed effects models for the datasets for which repeated tumor volume measurements over time were available. A mixed model is a statistical model containing both fixed and random effects: the fixed effect represents the average effect in the entire patient population, while the random effect represents individual patient effects. In equation (2) in the main text, *β_Carry_* and *b_Carry_i_* denote the fixed and random effects of LAI/MAI, and *β_r_* and *b_ri_* the fixed and random effects of growth rates, respectively. There is no relationship between fixed and random effects; for instance, *β_Carry_* is not necessarily negative in cases with a large *b_Carry_i_*. The use of non-linear mixed effects models for the fitting of the logistic growth model (equation (2) in the main text) to patient data resulted in numerical instabilities; thus the parameters in the model were non-identifiable. This observation might arise because parameters of LAI/MAI and *B* in equation (2) should have distributions consisting of positive values only, but not normal distributions. For this reason, we explored Bayesian inference allowing us to consider more flexible distributions of parameters in the models. We used the rstan package in R and parallelized eight chains for Markov Chain Monte Carlo (MCMC) sampling. To obtain posterior distributions, we used the informative prior distributions for growth rates *β_r_* that were obtained previously: the prior distribution for primary growth rates was *N*(0.16, 0.14), and that for metastasis was*N*(0.58, 2.72) (Haeno et al., 2012). We assumed lognormal priors for predicted tumor volumes, LAI/MAI, and *B* in equation (2) so that these parameters become positive. See Supplementary Figure legends for details of priors. To evaluate the accuracy of LAI, we performed a simulation study using the patient cohort (**S1 Doc(j)**). The model fit was assessed by AIC (Akaike Information Criterion) (Fang, 2011; Hurvich and Tsai, 1989). Overall survival (OS) was defined as the time from diagnosis to death and was analyzed using the Kaplan-Meier method. The Mann-Whitney U test was used to test whether two samples stemmed from the same distribution. A polyserial correlation coefficient was used to measure the correlation between a continuous variable and an ordered categorical variable. In any analyses, all values of *P* < 0.05 were considered indicative of statistical significance. All statistical analyses were performed using R version 3.3.0 software (R Foundation for Statistical Computation, Vienna, Austria). Libraries rstan, lmer, and nlme in R were used.

**S1 Doc (g). Computational studies for optimal treatment**

To investigate different treatment strategies using *in silico* clinical trials, we explored three scenarios: (i) CRTx administered to patients with LAPC; (ii) neoadjuvant CTx followed by standard adjuvant care; and (iii) administration of CTx according to different schedules in unresectable patients. The parameters for each patient are determined randomly according to distributions estimated from the MGH patient cohort. Note that an advantage of our *in silico* approach is that each case can receive both the standard and the experimental arms, so that we can evaluate several experimental regimens for each case.

In scenario (i), simulated cases were stratified into two groups at diagnosis (**Fig 4A**): cases receiving three months of CTx followed by RTx and three months of adjuvant CTx (regimen 1); and cases receiving six months of CTx (regimen 2). In scenario (ii), simulated cases were stratified into two groups at diagnosis (**Fig 4C**): cases receiving four months neoadjuvant CTx followed by resection and six months adjuvant CTx (regimen 3); and cases receiving six months CTx following resection (regimen 4). In scenario (ii), each case was restaged at the time of resection, and those with metastases did not undergo subsequent surgical resection in the simulations. In scenario (iii), simulated cases were randomized into different treatment groups after diagnosis (**Fig 4E**): cases without any CTx (regimen 5); cases receiving four months CTx and two months cessation (regimens 6 and 9); cases receiving four months CTx (regimens 7); and cases receiving six months CTx (regimens 8). Side effects for FFX include neutropenia, thrombocytopenia, anemia, sensory neuropathy, diarrhea, and transaminitis, and the standard medical care for these side effects are discontinuation of FFX (Conroy et al., 2011). Our regimens with (i) temporary cessations followed by restart of FFX (regimen 6) and (ii) GEM/GEM+nab-paclitaxel as the second-line setting after FFX discontinuation (regimen 9) both incorporate FFX discontinuation consistent with this guideline. Note that the volume change of tumors does not reflect side effects of FFX but simply the effectiveness of FFX. In our simulation studies, a CTx regimen is continued until disease progression. Based on the largest clinical trial testing the superiority of FFX over GEM (Conroy et al., 2011), it is recommended to evaluate therapy response every 2 months using a CT scan. Consistent with this approach, we made observations of tumor volume in our simulated cases every 2 months to test the efficacy of various regimens. In all scenarios, simulations were performed until death. For each simulated case, we recorded OS, the number of tumor cells at the primary site, the number of metastases, and the number of metastatic cells per site. Parameters used in the simulations are described in each figure legend, and 100 cases were performed for each regimen. For these *in silico* clinical trials, sample sizes were determined as follows:

(i) CRTx for LAPC (**Fig 4A**): we included 96 cases without metastases in these simulations. The sample size of 96 in each arm (Regimen 1 and 2) was determined based on the following assumptions: an overall 5-year survival rate of 1% in the chemotherapy arm (Regimen 2) and an absolute increase in 5-year survival of 10% in the experimental arms (Rahib et al., 2014; Siegel et al., 2017). Assuming an accrual and follow-up periods of 0.001 and 60 months, respectively, testing with the log-rank test with a two-sided type I error of 0.05 and a power of 90% yields a sample size of 192 cases (96 cases per treatment group) (Lakatos, 1988). An advantage of the computational trials is their capability of enrolling cases within a zero period, which is impossible in clinical practice. A shorter accrual period is desirable to reduce the number of participants treated with a regimen that may be ineffective. Similarly, in a computational trial we can set the follow-up period as long as desired, which is not pragmatic in a clinical trial despite the fact that a longer follow-up period is beneficial for observing long-term outcomes.

(ii) Neoadjuvant CTx followed by standard adjuvant care (**Fig 4C**): we included 95 cases with resectable disease in these simulations. The sample size of 95 in each arm (Regimens 3 and 4) was determined based on the following assumptions: an overall 5-year survival rate of 20% in the standard arm (upfront surgery with adjuvant CTx) and an absolute increase of 20% in 5-year survival in the experimental arm (Gillen et al., 2010). Assuming an accrual and follow-up period of 0.001 and 60 months, respectively, testing with the log-rank test using a two-sided type I error of 0.05 and a power of 90% yields a sample size of 190 cases (95 cases per treatment group).

(iii) CTx at different time points in patients with metastatic disease (**Fig 4E**): to assess the non-inferiority of temporary cessation of CTx (regimens 6 or 9 vs. regimen 7), the non-inferiority margin of the temporary cessation of CTx was set at 1.33: the median OS in patients with metastatic disease receiving FFX was assumed to be 11.4 while that in those receiving FFX with the reduced cycles was 8.5. Non-inferiority was demonstrated within a margin of 33% at a one-sided significance level of 2.5% (alpha) and a power of 80%, with a sample size of 195 per arm (390 in total).

**S1 Doc (h). Supplementary discussion**

We explored a mixed effects model for datasets for which repeated tumor volume measurements were made over time for each patient, which allowed us to estimate parameters of tumor growth and LAI/MAI in each individual (**S2 and S3 Data**). This approach enabled us to distinguish different disease progression phenotypes and thus describe interpatient heterogeneity in PDAC. Considering higher frequency of tumor volume measurement is rarely available because sequential imaging data in the absence of treatment and during homogeneous therapy is rarely obtained in PDAC (Haeno et al., 2012). Our detailed estimations of these parameters offer a unique opportunity of understanding the dynamics of PDAC progression. Note however that untreated patients in our cohort refer to both those before the initiation of treatment, those in terminal stages, and potentially those for whom no information was available regarding prior treatment; this patient group might thus represent a heterogeneous cohort.

In this study, we predicted that smaller primary sites at death translate to a smaller number of metastases with larger sizes at death in simulated cases with CRTx (**Fig 4A** and **S4 Fig**). This phenomenon might arise because of (i) a reduction in the size of the primary site due to CRTx treatment and (ii) larger estimated growth rates for metastasis than for primary sites. When investigating the transition probabilities between states in the stochastic simulation (**S1 Doc(c)**, equations (4)), we observe that the number of primary cells *w* decreases during CRTx, leading to larger relative number of cells at metastatic site i (=*y_i_*). Along with larger estimated growth rates of metastasis (*r_2_*) than those of the primary site (*r_0_*) (**S2 Table**), the probability that the number of cells increases by one becomes larger in metastases than the primary site, which leads to larger metastases (**S1 Doc(c)**, equations (3)). Meanwhile, the smaller number of cells at the primary site due to CRTx leads to a smaller opportunity of new metastatic events arising after CRTx, which results in a smaller number of metastases. The existing small number of metastases then has a larger chance to increase in number, eventually resulting in the phenomenon of fewer but larger metastases at death. Even though larger metastases are expected at death, local control by CRTx remains important for patients who have local symptoms, as long as OS is superior in the CRTx arm compared to the CTx arm or comparable between them (**Figs 4A and 4B**).

In this paper, we only focused on investigating the dynamics of PDAC from an evolutionary perspective; there are also other types of quantitative analyses commonly utilized in drug development research or industry (Chakraborty and Murphy, 2014; EFPIA MID3 Workgroup et al., 2016). For instance, in drug development (EFPIA MID3 Workgroup et al., 2016), multiple types of information are collected to identify the optimal dose through pharmacokinetics/pharmacodynamics (PK/PD) analysis. Once such data becomes available, our model can incorporate these ideas; in fact, we have previously developed modeling platforms to address this need (Chakrabarti and Michor, 2017; Foo et al., 2012). In this approach we predicted the growth rates as a function of the dose/PK/PD information and then used the predicted growth rates as input into an evolutionary model. In another direction, if the collected data had included more information for each patient, one could have used statistical or machine learning approaches to predict the number of metastases for a patient given his/her treatment history and other clinical test results. Similar analyses can also be applied to predict survival. These analyses can also be incorporated into one single approach through hierarchical models. Along this line, a bolder goal could be set to prescribe an optimized regimen for each patient by tailoring collected information (Chakraborty and Murphy, 2014). Despite the fact that PK/PD data was not available in this study, we believe that our approach represents a clinically relevant method for inferring the macroscopic dynamics of PDAC progression along with interpatient viability. This observation arises because (i) the time horizon for patient management in the clinic is on a different scale than PK/PD measurements; and (ii) PDAC patients often have comorbidities of ascites, severe jaundice, diabetes mellitus, poor performance status etc, which perturb tumor dynamics/drug distributions. Another limitation is that our approach does not account for genetic heterogeneity of PDAC patients such as (epi)genetic alterations of BRCA1/2 or major proteins related to homologous recombination; in order to incorporate such information into our computational approach, we can adapt a previously published model of BRCA-associated cancer progression once genetic information becomes available for large cohorts with longitudinal volume measurements together with treatment information using DNA cross-linking agents such as platinum-based CTx or poly (ADP-ribose) polymerase inhibitors (Yamamoto et al., 2011, 2014). PDAC patients present with various symptoms such as pain, weight loss, jaundice, diabetes mellitus, poor performance status etc. In the model, however, we did not incorporate any parameters on symptoms, as they are considered to be the consequence of tumor invasion into other organs but not a causative factor of tumor progression.

It is possible that there are measurement errors in the tumor volume data, for example due to low resolution of tumor CT images and human error. However, the use of priors with large variances provided robust and sensible results, suggesting that the measurement errors did not impact our inference of growth rates or LAI/MAI. The posterior distributions for *LAI* and *B* (see Eq. (2)) could be estimated using non-informative priors, so we used *LN*(0, 10) and *LN*(0, 10) as prior distributions, respectively. The posterior distribution for growth rate parameter *β_r_* could not be estimated when a non-informative prior was used, however. This finding indicates that the logistic growth model with patient-specific effects on all the parameters is difficult to estimate given the relatively sparsely measured tumor volume data, and that either a higher frequency of tumor volume measurements or strong priors from the literature are necessary. Similar observations were made under different treatment modalities in **S3 Data**. We did not have access to additional tumor volume measurements, and therefore used informative priors based on previous studies (Haeno et al., 2012; Iacobuzio-Donahue et al., 2009). Since it is possible for an informative prior to have a large impact on the posterior distribution, we performed sensitivity analyses to examine the effect of different priors on the posteriors (**S3 Data**) (Gelman et al., 1995). In **Data (i)**–**(iv)** and **Data (v)**–**(viii)** of **S3 Data**, we investigated how the prior distributions impact the posterior distribution for primary and metastatic growth rates and LAI and MAI without treatment in **Data (i)** and **(v)** of **S3 Data** and with different treatment modalities in **Data (ii)**–**(iv)** and **Data (vi)**–**(viii)** of **S3 Data**, respectively. For instance, in the absence of treatment, the posterior distribution for the primary growth rate does not change if we fix the prior distribution N(0.16, 0.14) of the growth rates and increase the flatness of the prior distribution of the other parameters in the logistic growth model (**Data (i)A-(i)C** of **S3 Data**); however, if we relax the variance parameter in the prior distribution for growth rates, the posterior distributions for the parameters in logistic growth model (*r*, *B*, and LAI) are indeed sensitive to such relaxation (**Data (i)D** of **S3 Data**). In this study, prior distributions for primary growth rates were obtained from *N*(0.16, 0.14) and those for metastasis were obtained from *N*(0.58, 2.72) based on a previous study (Haeno et al., 2012). In addition, we observed a flat region in the beginning of the survival curves in **S3 Fig**, which might be due to our assumption that death depends only on the total number of cells but not unexpected events such as biliary sepsis or gastrointestinal obstruction.

**S1 Doc (i). Stan code for Bayesian inference**

data {

int<lower = 0> Nobs; int<lower = 0> Nids; int<lower = 1, upper = Nids> id[Nobs]; vector[Nobs] Volume; vector[Nobs] Time;

}

parameters {

real<lower = 0.01> sigmaeps; vector<lower = 0>[Nids] Asym; vector[Nids] r; real<lower = 0> B;

}

transformed parameters {

vector[Nobs] yhat; for (i in 1:Nobs) yhat[i] <- Asym[id[i]] / (1 + B * exp(- r[id[i]] * Time[i]));

}

model {

Asym ~ lognormal(0, 10); r ~ normal(0.16, 0.374); B ~ lognormal(0, 10); Volume ~ lognormal(log(yhat), sigmaeps);

}

**S1 Doc (j). Evaluation of the accuracy of LAI**

The LAI of a patient is defined as the tumor size at which growth levels off so that the tumor does not increase in size beyond the LAI. The true LAI of a patient will become apparent as time goes on; however, not all patients have tumor size measurements available at that time. To evaluate the accuracy of our LAI estimates, we performed a simulation study using the patient cohort. In brief, we constructed a simulated dataset containing multiple time point tumor volume measurements that were predicted using equation (2). To obtain tumor volume measurements at time *t*, LAI and corresponding growth parameters were randomly chosen from the distributions estimated from the patient cohorts. To mimic clinical situations, we considered a certain extent of volume measurement error (x%) and also variability in the time points at which measurements were made relative to a pre-set schedule (y%). Then, using this simulated dataset, we estimated LAI_simulate_ by Bayesian inference and identified the correlation of this measure with the true (known) LAI. One simulated dataset contains 100 cases; we repeated this simulation study 10 times for each condition of (x,y) and investigated the average correlation coefficient between estimated and true LAI. We found that correlation coefficients were 0.99 when (x,y)=(10,10); 0.97 when (x,y)=(30,30); and 0.90 when (x,y)=(50,50). Based on these results, we concluded that we are able to very accurately classify a patient with regard to his/her LAI.
