# Supplementary figures and images for: Computational modeling of pancreatic cancer patients receiving FOLFIRINOX and gemcitabine-based therapies identifies optimum intervention strategies
Source: PLoS One. 2019 Apr 26;14(4):e0215409. doi: 10.1371/journal.pone.0215409 (PMC6485645; doi:10.1371/journal.pone.0215409)

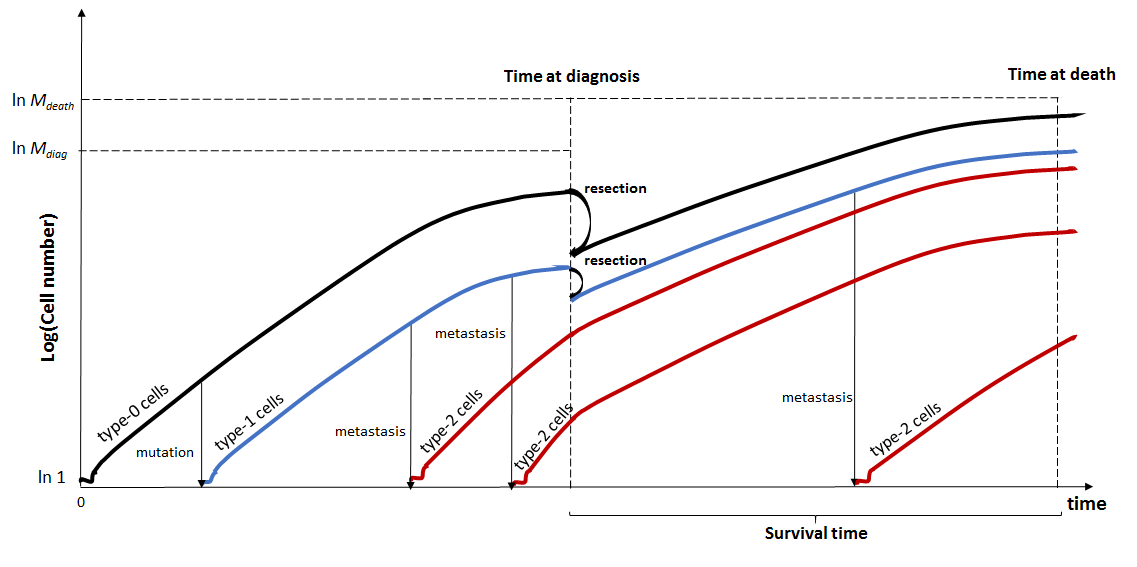

Supplement: S1 Fig — We considered a model of logistic expansion of the number of cancer cells starting from a single cell in the primary site. Cancer cells follow a stochastic process: during each elementary time step, cells may divide with a possibility of accumulating an alteration that allows it to divide, die, or metastasize elsewhere. We considered the situation in which the growth rate of the tumor decreases with increasing tumor size. Cells that have not yet evolved the ability to metastasize, type-0 cells, divide at rate r0(1-(w+x)/LAI) and die at rate d0 per unit time. Type-0 cells give rise to type-1 cells through accumulating an alteration in a metastatic-related gene with probability u per type-0 cell division. Type-1 cells divide and die at rates of r1(1-(w+x)/LAI) and d1 per unit time. Type-1 cells can establish a metastatic colony, consisting of type-2 cells, at another location with probability q; these sites start from a single metastatic cell in each metastatic site. Type-2 cells grow with a division and death rate of r2(1-yi/MAIi) and d2 per unit time, respectively. When the total number of all tumor cells reaches Mdiag, the tumor is detected and treatment in the form of chemotherapy, radiation, and/or surgery initiates. When the total number of cells reaches Mdeath, the patient dies. See S1(B)–S1(D) Doc for a description of the model. (TIF) [file pone.0215409.s005.tif]

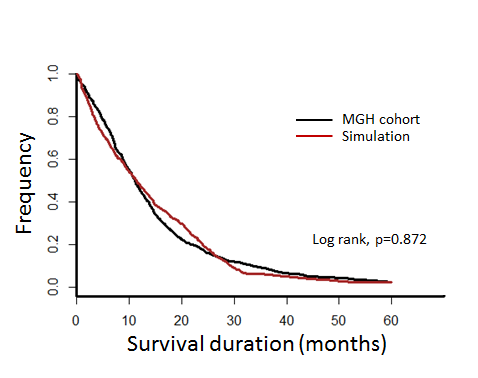

Supplement: S2 Fig — The panel shows overall survival of patients in the MGH cohort (black line) and simulated cases (red line). Parameter values are u = 6.31×10^−5^, q = 6.31×10^−7^, r0 and r1 = 0.28, r2 = 1.16, death rate of each type = 1/100×growth rate, Mdiag = 10N(9.47,0.29), and Mdeath = 1010.6. LAI/MAI and growth rates during CTx for primary and metastatic sites are based on the estimated distributions (S2 and S3 Data), and ε was randomly chosen from [10−5, 10−1]. The number of simulated cases was the same as the number of patients in the clinical cohort (n = 1,089). (TIF) [file pone.0215409.s006.tif]

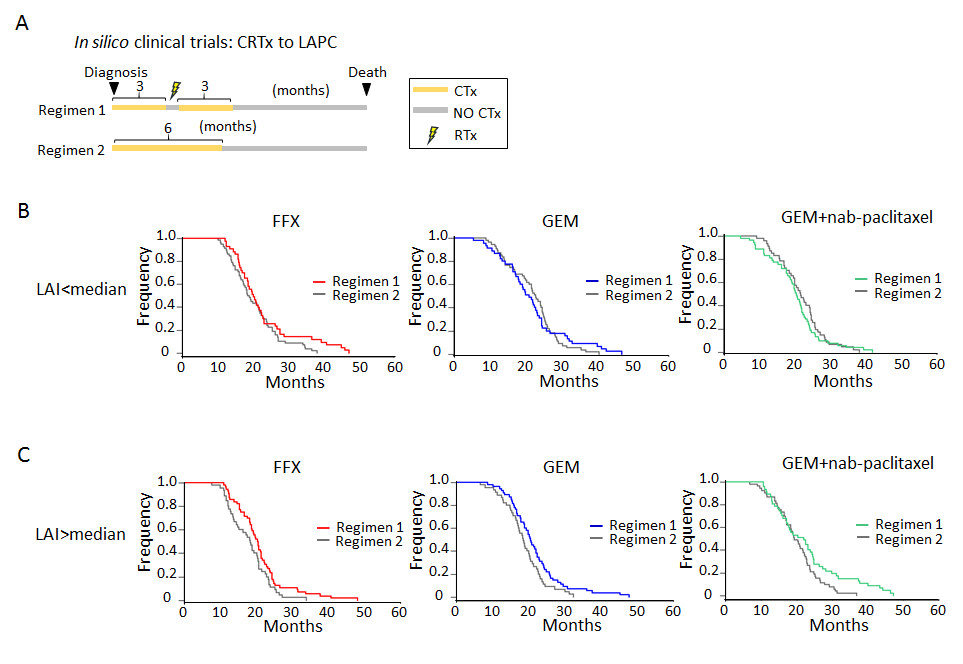

Supplement: S3 Fig — (A) Description of CRTx schedules for LAPC cases. (B and C) Assessment of survival of regimens 1 and 2 in simulated cases with LAPC (B) whose LAI is less than the median, and (C) whose LAI is larger than the median. The median LAI = 1.2×10^10^, 1.4×10^10^, and 1.4×10^10^ for FFX, GEM, and GEM+nab-paclitaxel, respectively. Number of simulated cases was 50 per group; and P-values by log-rank test were 0.117, 0.809, and 0.466 for FFX, GEM, and GEM+nab-paclitaxel in (B); and 0.0547, 0.0429, and 0.0379 for FFX, GEM, and GEM+nab-paclitaxel in (C). Parameter values were the same as those in S2 Fig. (TIF) [file pone.0215409.s007.tif]

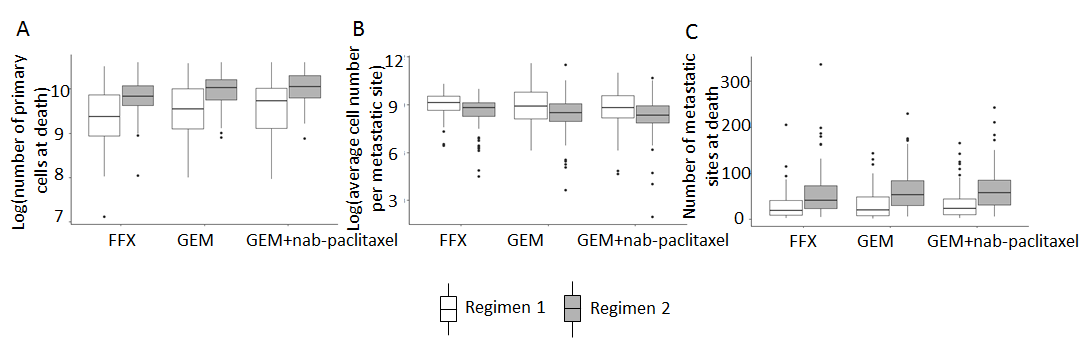

Supplement: S4 Fig — (A–C) Assessment of (A) the primary tumor size at death; (B) the average size of metastatic sites at death; and (C) the number of metastases at death with different chemotherapies in each regimen. Number of simulated cases was 100 per category, and P<0.001 for each pair of regimens using any drug in (A)–(C). Parameter values used for the panels were the same as those described in S2 Fig. (TIF) [file pone.0215409.s008.tif]

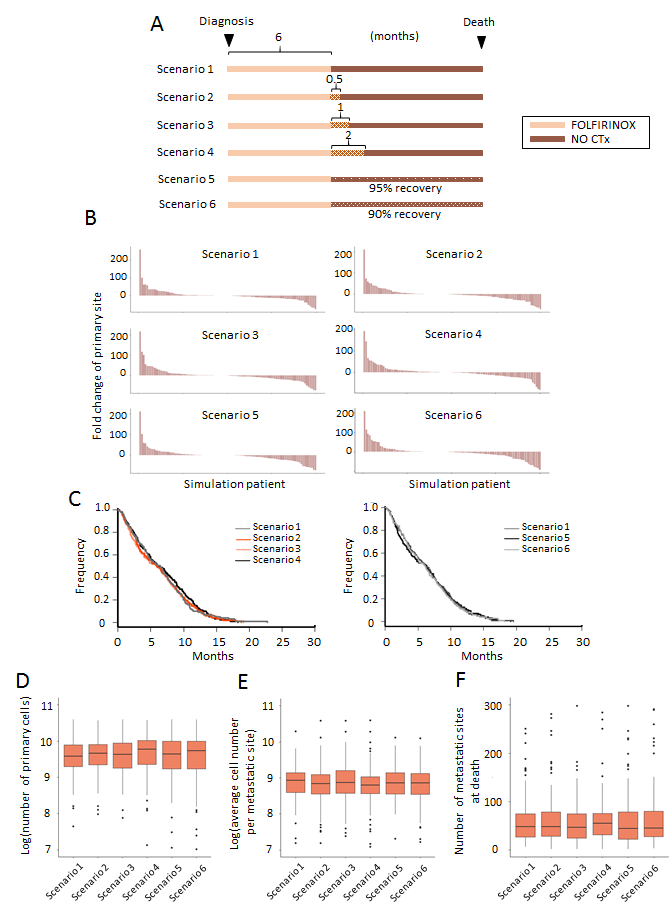

Supplement: S5 Fig — (A) Description of tested CTx schedules with regard to our sensitivity analyses of the assumption of growth rate reversibility after treatment. In scenarios 2–4, the time until the growth rates recover to pre-treatment levels after treatment discontinuation is 2 weeks, 1 month, and 2 months, respectively. In scenarios 5 and 6, the levels the growth rate recovers to after treatment discontinuation are 95% and 90% of the original growth rate levels, respectively. (B) Waterfall plot of relative changes in diameters of simulated primary tumors at death compared with those at diagnosis in each scenario. P>0.05 for comparisons of all pairs of scenarios. (C) Assessment of survival duration. P>0.05 for comparisons of all pairs of scenarios 1–4; P>0.05 for scenarios 1 vs 5 and 1 vs 6. (D-F) Assessment of (D) the primary tumor size at death; (E) the average size of metastatic sites at death; and (F) the number of metastases at death in each scenario. P>0.05 for each pair of scenarios. Number of simulation cases are 100 per each scenario. Parameter values used are the same as those described in S2 Fig. (TIF) [file pone.0215409.s009.tif]
