# Supplementary material for: Computational modeling of pancreatic cancer patients receiving FOLFIRINOX and gemcitabine-based therapies identifies optimum intervention strategies
Source: PLoS One. 2019 Apr 26;14(4):e0215409. doi: 10.1371/journal.pone.0215409 (PMC6485645; doi:10.1371/journal.pone.0215409)
Supplement: S1 Table — The Akaike Information Criterion is shown to assess model fits to the data. The models include the logistic model, the linear model, and the exponential model. (DOCX) [file pone.0215409.s010.docx]

**S1 Table.** Comparison of model fitting according to AIC

|  | Primary site | | | Metastasis | | |
| --- | --- | --- | --- | --- | --- | --- |
| sample # | 93 | | | 95 | | |
|  | Logistic | Linear | Exponential | Logistic | Linear | Exponential |
| AIC | -107 | -101 | 583 | 270 | 587 | 1032 |

AIC = Akaike Information Criterion
