# Supplementary material for: Computational modeling of pancreatic cancer patients receiving FOLFIRINOX and gemcitabine-based therapies identifies optimum intervention strategies
Source: PLoS One. 2019 Apr 26;14(4):e0215409. doi: 10.1371/journal.pone.0215409 (PMC6485645; doi:10.1371/journal.pone.0215409)
Supplement: S2 Table — Estimated growth rates during CTx and in the absence of treatment are summarized and shown in the values of median, first and third quartiles. Also, the P-values by Mann-Whitney test are shown for the comparison of metastatic growth rates among the treatments. (DOCX) [file pone.0215409.s011.docx]

**S2 Table.** Median, 1^st^ and 3^rd^ quartiles for estimated growth rates and LAI/MAI using the logistic growth model.

|  | Median | 1st quartile | 3rd quartile |
| --- | --- | --- | --- |
| Primary growth rate | 0.280 | 0.160 | 0.417 |
| Metastatic growth rate | 1.159 | 0.610 | 1.767 |
| Primary growth rate during GEM | 0.157 | -0.024 | 0.280 |
| Metastatic growth rate during GEM | 0.915 | 0.119 | 1.322 |
| Primary growth rate during FFX | -0.022 | -0.181 | 0.203 |
| Metastatic growth rate during FFX | 0.934 | 0.474 | 1.116 |
| Primary growth rate during GEM+nab-paclitaxel | 0.052 | -0.081 | 0.300 |
| Metastatic growth rate during GEM+nab-paclitaxel | 1.420 | 0.479 | 1.732 |
| LAI | 0.586 | 0.299 | 1.075 |
| MAI | 0.452 | 0.138 | 1.675 |

*The time unit for growth rates is 1/month.

P-values using the Mann-Whitney test for comparing primary growth rates.

| Regimen | No-treatment | GEM | GEM+nab-paclitaxel | FFX |
| --- | --- | --- | --- | --- |
| No-treatment |  |  |  |  |
| GEM | p<0.001 |  |  |  |
| GEM+nab-paclitaxel | p<0.001 | 0.261 |  |  |
| FFX | p<0.001 | p<0.001 | 0.0472 |  |

P-value using the Mann-Whitney test for comparing metastatic growth rates.

| Regimen | No-treatment | GEM | GEM+nab-paclitaxel | FFX |
| --- | --- | --- | --- | --- |
| No-treatment |  |  |  |  |
| GEM | 0.00108 |  |  |  |
| GEM+nab-paclitaxel | 0.098 | 0.00981 |  |  |
| FFX | p<0.001 | 0.0482 | 0.00172 |  |

*P-values were adjusted using the Benjamini-Hochberg procedure to account for multiple testing.
