# Supplementary material for: Computational modeling of pancreatic cancer patients receiving FOLFIRINOX and gemcitabine-based therapies identifies optimum intervention strategies
Source: PLoS One. 2019 Apr 26;14(4):e0215409. doi: 10.1371/journal.pone.0215409 (PMC6485645; doi:10.1371/journal.pone.0215409)
Supplement: S3 Table — The log-rank test was performed for the group comparisons regarding the Kaplan-Meier analyses shown in Fig 4F. (DOCX) [file pone.0215409.s012.docx]

**S3 Table.** P-values using the log-rank test for comparisons of the four chemotherapies regimens in the Kaplan-Meier analysis shown in **Figure 4F**.

| Regimen | 5 | 6 | 7 | 8 |
| --- | --- | --- | --- | --- |
| 5 |  |  |  |  |
| 6 | p<0.001 |  |  |  |
| 7 | p<0.001 | 0.889 |  |  |
| 8 | p<0.001 | p<0.001 | p<0.001 |  |

**FFX**

**GEM**

| Regimen | 5 | 6 | 7 | 8 |
| --- | --- | --- | --- | --- |
| 5 |  |  |  |  |
| 6 | 0.0228 |  |  |  |
| 7 | 0.0341 | 0.722 |  |  |
| 8 | p<0.001 | 0.0186 | 0.0214 |  |

**GEM+nab-paclitaxel**

| Regimen | 5 | 6 | 7 | 8 |
| --- | --- | --- | --- | --- |
| 5 |  |  |  |  |
| 6 | 0.0478 |  |  |  |
| 7 | 0.0327 | 0.695 |  |  |
| 8 | 0.00139 | 0.0951 | 0.140 |  |

FFX was significantly superior to GEM (P-value = 0.0038 by log-rank test) or GEM+nab-paclitaxel (P value = 0.011 by log-rank test) in regimen 8.

*P-values were adjusted using the Benjamini-Hochberg procedure to account for multiple testing.
