# Supplementary material for: Computational modeling of pancreatic cancer patients receiving FOLFIRINOX and gemcitabine-based therapies identifies optimum intervention strategies
Source: PLoS One. 2019 Apr 26;14(4):e0215409. doi: 10.1371/journal.pone.0215409 (PMC6485645; doi:10.1371/journal.pone.0215409)
Supplement: S4 Table — The log-rank test was performed for the group comparison regarding the Kaplan-Meier analyses shown in Fig 4G. (DOCX) [file pone.0215409.s013.docx]

**Table S4.** P-values using the log-rank test for comparisons of the three regimens with cessations in the Kaplan-Meier analysis shown in **Figure 4G**.

| Regimen | 6 | 9(Second-line=GEM) | 9(Second-line= GEM+nab-paclitaxel ) |
| --- | --- | --- | --- |
| 6 |  |  |  |
| 9(Second-line=GEM) | 0.142 |  |  |
| 9(Second-line=GEM+nab-paclitaxel) | 0.213 | 0.35 |  |

*P-values were adjusted using the Benjamini-Hochberg procedure to account for multiple testing.
